# Time-dependent solid-state molecular motion and colour tuning of host-guest systems by organic solvents

**DOI:** 10.1038/s41467-019-13844-5

**Published:** 2020-01-07

**Authors:** Yu-Dong Yang, Xiaofan Ji, Zhi-Hao Lu, Jian Yang, Chao Gao, Haoke Zhang, Ben Zhong Tang, Jonathan L. Sessler, Han-Yuan Gong

**Affiliations:** 1 College of Chemistry, Beijing Normal University, Xinjiekouwaidajie 19, Beijing, 100875 P. R. China; 2 Department of Chemistry, HKUST Jockey Club Institute for Advanced Study, Institute of Molecular Functional Materials, Division of Biomedical Engineering, State Key Laboratory of Molecular Neuroscience, Division of Life Science, The Hong Kong University of Science and Technology, Clear Water Bay, Kowloon, Hong Kong China; 3 Department of Chemistry, The University of Texas at Austin, 105 East 24th Street, Stop A5300, Austin, TX 78712-1224 USA; 4 Department of Chemistry and Center for Supramolecular Chemistry and Catalysis, Shanghai University, 99 Shangda Road, Shanghai, 200444 China

**Keywords:** Optical materials, Crystal engineering, Self-assembly

## Abstract

Host-guest complex solid state molecular motion is a critical but underexplored phenomenon. In principle, it can be used to control molecular machines that function in the solid state. Here we describe a solid state system that operates on the basis of complexation between an all-hydrocarbon macrocycle, ***D***_**4d**_**-CDMB-8**, and perylene. Molecular motion in this solid state machine is induced by exposure to organic solvents or grinding and gives rise to different co-crystalline, mixed crystalline, or amorphous forms. Distinct time-dependent emissive responses are seen for different organic solvents as their respective vapours or when the solid forms are subject to grinding. This temporal feature allows the present ***D***_**4d**_**-CDMB-8**⊃perylene-based system to be used as a time-dependent, colour-based 4th dimension response element in pattern-based information codes. This work highlights how dynamic control over solid-state host-guest molecular motion may be used to induce a tuneable temporal response and provide materials with information storage capability.

## Abstract (web summary)

Host-guest solid state molecular motion is a critical but underexplored phenomenon which can be used to control molecular machines that function in the solid state. Here, the authors describe a solid state machine that shows solvent vapour- and mechanically-induced molecular motion that allows access to different crystalline and amorphous forms.

## Introduction

Solid-state molecular motion is a critical but underexplored phenomenon in nature. However, it is becoming appreciated as a powerful tool in crystal engineering that can be exploited to produce inter alia highly efficient catalysts^1–4^, gas storage systems^5–8^, and molecular machines (e.g., molecular switches^9–14^, rotors^15–20^, and shuttles^21^). The discovery of unusual solid-state molecular motion phenomena is of interest; along with accompanying dynamic regulation strategies such findings could allow for further advances in this area and might lead to the preparation of functional materials^22–29^.

Molecular motion involving host-guest complexes^30–32^ (e.g., inclusion complexes, pseudo rotaxanes) and mechanically interlocked molecules (MIMs)^33,34^ (e.g., rotaxanes and catenanes) has been extensively explored to create molecular machines that operate under solution phase conditions^35^. In contrast to what is seen in solution, where molecular motion is typically facile, the close packing characteristic of most solid-state forms makes molecular movement and its controlled induction difficult. This has made it challenging to develop systems displaying motion in the solid state. Finding ways to regulate this motion has proved even more difficult. One approach could involve the use of liquid additives (e.g., solvents) that could serve as effective mobilizers to promote motion within the solid state. The viability of this strategy has been documented in the case of liquid crystals^36–38^, classic industrial plastic processes^39–41^, and several crystal-to-crystal transformations^42–45^. However, to our knowledge, solid-state molecular machines that rely on the solvent-based modification of host-guest complexes to achieve molecular motion have not been reported. Nor have time-dependent, motion-induced changes in the emissive features of solid materials been observed. To the extent such effects are demonstrated it could lead to an ostensibly new type of molecular machine where solvents provide the fuel to drive the system and motion is reflected in easy-to-discern changes in the solid-state properties. In the limit, such machines could provide dynamic constructs that allow information to be stored, manipulated, and read out in an input-specific, time-dependent manner.

**Fig. 1:**
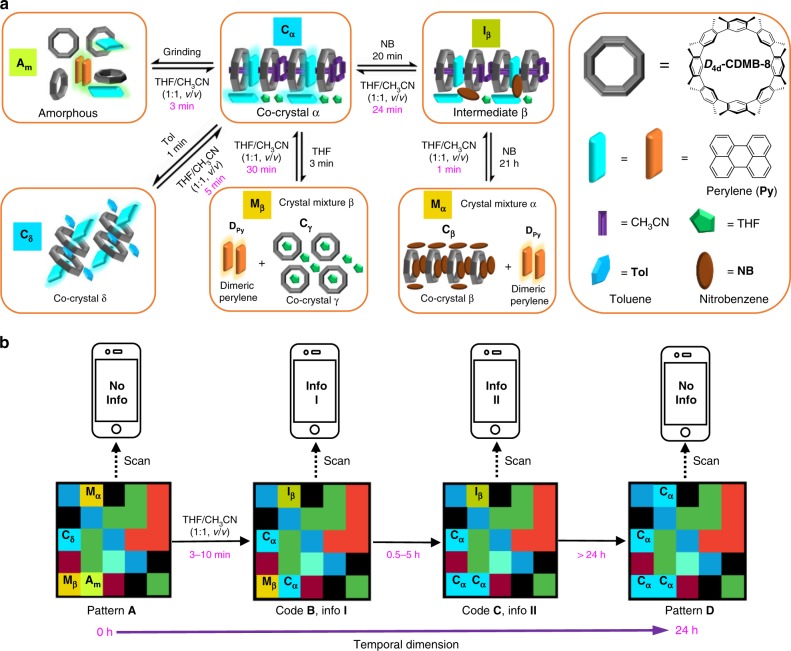
Schematic representations of solvent promoted solid-state molecular motion involving host-guest complexes and the 4D information coding the accompanying changes permit. **a** An organic solvent, applied to the solid forms as either a liquid or vapour, promotes solid-state molecular motion and leads to transformation between co-crystalline phases, as well as production of mixed crystalline states and an amorphous form. Also shown by use of highlighting (light blue vs orange) is the time resolved colour response produced under both room light and UV illumination (365 nm). See text for further details. **b** 4D information coding via a solvent-induced time-dependent vapoluminescent response.

Here we report a solid-state molecular machine based on an all-hydrocarbon host-guest construct derived from ***D***_**4d**_**-CDMB-8** and perylene (**Py**). As-prepared crystalline forms of ***D***_**4d**_**-CDMB-8** and **Py** react with a variety of solvent molecules (e.g., acetonitrile, THF, nitrobenzene, toluene, etc.) to produce several solid-state forms. These forms contain (1) a core host-guest complex stabilized by non-covalent π–π and CH–π interactions between the ***D***_**4d**_**-CDMB-8** host and the **Py** guest and (2) a liquid domain arising from solvent capture that promotes molecular movement in the solid state. As detailed below, different solid-state forms, including, (i) co-crystalline phase, (ii) mixed crystalline, and (iii) amorphous state materials are obtained in the presence or absence of different solvents. These forms are characterized by different emissive features. This allows the various solvents to be distinguished. Other stimuli, such as mechanical impact (e.g., grinding), also lead to changes in the structural and optical properties. Moreover, a time-dependent response is seen. Systems derived from ***D***_**4d**_**-CDMB-8** and **Py** thus act as rudimentary molecular machines where the inputs are solvents and the result of motion is a readily discernible structural and optical response (Fig. 1a). The time-dependent nature of the transformations, as well as its origins in the choice of solvent and solid-state forms, has allowed the ***D***_**4d**_**-CDMB-8** and **Py** system to be elaborated to produce a set of 4D codes wherein time-dependent changes provide one information coding dimension and colours and patterns the other three (Fig. 1b).

## Results

### Host-guest system solid-state molecular motion determinants

**Fig. 2:**
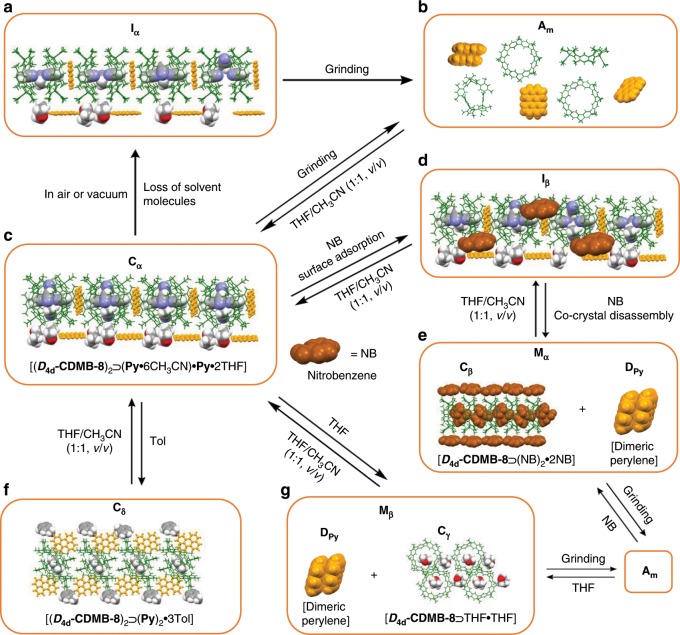
Complexation and decomposition of host-guest complexes involving solid-state molecular motion. Structures of, and transformations between, the co-crystalline material **C**_**α**_, mixed crystalline species (e.g., **M**_**α**_ and **M**_**β**_), co-crystalline materials (i.e., **I**_**α**_, **I**_**β**_, and **C**_**δ**_), and the amorphous form (**A**_**m**_) produced by exposure to organic solvents (vapour or liquid forms) or other treatments (e.g., grinding). **a**, **b**, **d**, **e**, **g** Suggested structures of **I**_**α**_, **A**_**m**_, **I**_**β**_, **M**_**α**_, and **M**_**β**_ base on PXRD analysis, ^1^H NMR, and fluorescence spectroscopic studies. **c**, **f** Single crystal structure of co-crystals **C**_**α**_ and **C**_**δ**_.

**CDMB-8** is an all-hydrocarbon macrocycle that exists in the form of two isomers, which are not easily interconverted^46^. In our initial study, we found that one isomer, namely ***D***_**4d**_**-CDMB-8**, could act as a good receptor for curved aromatic molecules (e.g., C_60_ and C_70_) both in solution and the solid state^46^. This finding led us to explore whether the isomeric species, ***C***_**s**_**-** and ***D***_**4d**_**-CDMB-8**, would interact with planar aromatic molecules. Perylene (**Py**) was chosen for initial studies. It was found that little if any discernible change in either the UV–Vis absorption, fluorescence emission or ^1^H NMR spectra was seen when **Py** was mixed with either ***C***_**s**_**-** or ***D***_**4d**_**-CDMB** (Supplementary Figs. 2–5). On the other hand allowing a mixture of ***D***_**4d**_**-CDMB-8** containing 1 molar equiv. of **Py** in THF/CH_3_CN (1/1, *v*/*v*) to undergo slow evaporation yielded green-yellow prismatic single crystals of [(***D***_**4d**_**-CDMB-8**)_2_⊃(**Py**•6CH_3_CN)•**Py**•2THF] (a solid-state construct referred to as **C**_**α**_, Fig. 2c). Evidence for the formation of a ***D***_**4d**_**-CDMB-8**⊃**Py** co-crystalline complex in **C**_**α**_ came from a single crystal X-ray diffraction analysis (Supplementary Figs. 6–8). Each periodic repeat unit contains two macrocycles and two perylenes, as well as six CH_3_CN and two THF molecules.

Two interaction modes were observed for the perylene guests. One guest is seen to reside outside of ***D***_**4d**_**-CDMB-8** being held there through presumed CH–π interactions. Another perylene molecule is located between two neighbouring macrocyclic cavities, being stabilized through possible CH-π and π-π interactions with ***D***_**4d**_**-CDMB-8**. An extended 1D linear packing structure is seen. An unusual cluster of six CH_3_CN molecules serves as bridge between two inserted perylene moieties. Further study revealed that **C**_**α**_ could be easily prepared on a gram scale using the above approach (Supplementary Movie 1). A powder X-ray diffraction (PXRD) analysis of the resulting bulk sample proved in good agreement with the simulated PXRD pattern calculated using the single crystal data for **C**_**α**_ (Supplementary Fig. 9).

When **C**_**α**_ was allowed to stand in the air for six days or subject to vacuum for 5 h at ambient temperature, partial loss of solvent was seen. The resulting solid form, referred to as **I**_**α**_, is characterized by lattice features that are similar to those seen in **C**_**α**_ (Fig. 2a and Supplementary Figs. 16–19). In order to remove all the organic solvent molecules present in the complex between ***D***_**4d**_**-CDMB-8** and **Py**, **C**_**α**_ was subject to grinding using a mortar and pestle. This action produced an amorphous material (**A**_**m**_, Fig. 2b), as inferred from a PXRD analysis (Supplementary Fig. 16b). A ^1^H NMR spectral study revealed that **A**_**m**_ contains only macrocycle and perylene and is free of residual organic species (Supplementary Fig. 20). The same **A**_**m**_ product can also be generated easily by simply grinding a 1:1 molar ratio mixture of ***D***_**4d**_**-CDMB-8** and **Py** in the absence of a solvent. Treating **A**_**m**_ with THF/CH_3_CN (1/1, *v*/*v*), either by mixing with the solvents directly or exposing the solid material to their vapours (assumed to be saturated in air at 298 K), served to regenerate **C**_**α**_ (Supplementary Fig. 16c).

The weak interactions within **C**_**α**_ as inferred from the initial single crystal X-ray diffraction studies led us to hypothesize that the above liquid and organic vapour-induced phenomenological changes were structural in origin. In an initial effort to test this hypothesis, studies were carried out using nitrobenzene (NB) vapour. In fact, a particularly complex response was seen. A combination of ^1^H NMR spectral, in situ single crystal X-ray diffraction, and PXRD analyses (Supplementary Figs. 23 and 26) leads us to suggest that a stable intermediate material (**I**_**β**_) is produced as the result of nitrobenzene vapour covering the surface of **C**_**α**_ in 20 min (Fig. 2d). In this case, little apparent nitrobenzene-induced molecular motion is observed, and the lattice parameters associated with ***D***_**4d**_**-CDMB-8** and **Py** are retained.

Over longer time scales (i.e., from 3 to 21 h post exposure), a crystalline transformation is seen. Structural analyses provided support for the notion that all the components within **I**_**β**_ undergo nitrobenzene vapour-induced motion to produce **M**_**α**_, a mixed crystalline solid material containing separate crystals of both dimeric perylene (**D**_**Py**_) and [***D***_**4d**_**-CDMB-8**⊃(NB)_2_•2NB] (**C**_**β**_) (Fig. 2e, Supplementary Figs. 23 and 27). Based on this observation, we infer that nitrobenzene promotes molecular motion in the solid state by providing liquid domains within the crystals of **C**_**α**_.

Support for the above suggestion came from the finding that when crystals of **C**_**α**_ were recrystallized from nitrobenzene, product **M**_**α**_ was also obtained. The mixed crystalline material, **M**_**α**_, could be transformed back to **I**_**β**_ and **C**_**α**_, albeit with different dynamics (1 min vs. 24 h, respectively), via exposure to THF/CH_3_CN (1/1, *v*/*v*) vapour (Supplementary Fig. 25). Dissolution and recrystallization of **M**_**α**_ in THF/CH_3_CN (1/1, *v*/*v*) also allowed for recovery of **C**_**α**_. To our knowledge, this solvent-induced transformation between a co-crystalline species containing a host-guest complex and a corresponding crystalline mixture containing separated host and guest species is without precedent in the literature. It represents what to our knowledge is a unique type of solid-state molecular motion. Thus, efforts were made to explore it further.

As a first step, we sought to explore the effect of different organic solvents. When **C**_**α**_ was exposed to THF vapour, a combination of ^1^H NMR spectral, PXRD, and X-ray diffraction analyses (Supplementary Figs. 31–33) provided support for the conclusion that a new crystalline mixture, referred to as **M**_**β**_, containing crystals of both [***D***_**4d**_**-CDMB-8**⊃THF•THF] (**C**_**γ**_) and **D**_**Py**_, was produced (Fig. 2g). When **C**_**α**_ was dissolved in THF and subject to slow evaporation, only blocks of colourless **C**_**γ**_ and yellow **D**_**Py**_ crystals were obtained as deduced from a single crystal diffraction analysis. On this basis, we propose that the co-crystalline species **C**_**α**_ undergoes disassembly upon exposure to THF, which then allows formation of the mixed crystalline material, **M**_**β**_. Under identical exposure conditions THF vapour promotes this conversion much faster (3 min) than nitrobenzene vapour (21 h). Again, apparent molecular motion underlies the observed solid-state structural switching (Supplementary Fig. 32).

Exposure of the co-crystalline form **C**_**α**_ to toluene (Tol), either in liquid or vapour form, led to the formation of another co-crystalline solid (referred to as **C**_**δ**_; Fig. 2f). Based on a qualitative comparison of the structures involved, considerable molecular motion is associated with this transformation. The PXRD pattern of **C**_**δ**_ proved to be a good match with the simulated pattern for a new co-crystalline material [(***D***_**4d**_**-CDMB**-8)_2_⊃(**Py**)_2_•3Tol] (Supplementary Fig. 36b). ^1^H NMR spectral studies of **C**_**δ**_ in CDCl_3_ exposed to toluene vapour provided support for the notion that 3 molar equiv. of toluene replace all the THF and CH_3_CN molecules originally present in **C**_**α**_ thus producing **C**_**δ**_ (Supplementary Fig. 36c). When **C**_**α**_ was dissolved in toluene and subject to slow evaporation, only green-yellow blocks of [(***D***_**4d**_**-CDMB-8**)_2_⊃(**Py**)_2_•3Tol] crystals were obtained as deduced from single crystal diffraction analyses. A single crystal X-ray diffraction structure of this material (i.e., [(***D***_**4d**_**-CDMB-8**)_2_⊃(**Py**)_2_•3Tol]) revealed that the perylene guest is inserted head on into the cavity of the ***D***_**4d**_**-CDMB-8** host and that one toluene molecule is located between two ***D***_**4d**_**-CDMB-8** macrocycles (Supplementary Figs. 12, 13). On this basis, we propose that the **C**_**α**_ co-crystalline material undergoes solid-state molecular motion upon exposure to toluene vapour and then forms a new co-crystalline material, **C**_**δ**_. This process is reversible. Exposing **C**_**δ**_ to saturated THF/CH_3_CN (1/1, *v*/*v*) vapour served to regenerate **C**_**α**_ (Supplementary Fig. 37). Under conditions identical to those used in the case of nitrobenzene and THF, the toluene vapour-induced conversion between **C**_**α**_ and **C**_**δ**_ proved relatively fast (1–5 min).

### Emission-based colour response

**Fig. 3:**
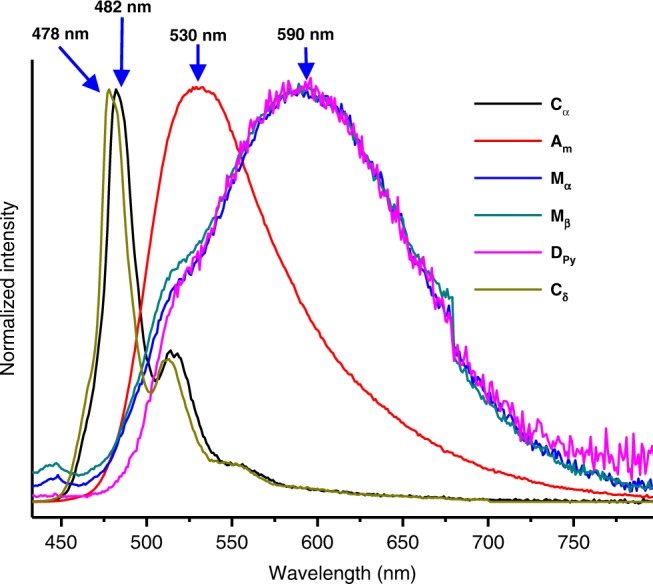
Normalized emission spectra of various solid forms considered in this study. The spectra of **C**_**α**_, **A**_**m**_, **M**_**α**_, **M**_**β**_, **D**_**Py**_, and **C**_**δ**_ are shown (*λ*_em_ = 365 nm).

A change in the fluorescence colour from green to orange was seen when **C**_**α**_ was converted to a mixed crystalline form (e.g., **M**_**α**_ or **M**_**β**_) by exposure to various organic solvents (either as vapours or used as for recrystallization) (cf. Supplementary Movie 2, and Supplementary Figs. 28, 34, and 46). The corresponding emission band appears at around 590 nm under conditions of UV illumination (*λ*_ex_ *=* 365 nm). This matches the emission produced by the presumably aggregated **Py** in crystalline **D**_**Py**_. This correspondence was taken as evidence that mixed crystalline solids, such as **M**_**α**_ or **M**_**β**_, contain **D**_**Py**_ crystalline domains and that these are responsible for the observed emission features. **I**_**α**_ and amorphous material (**A**_**m**_) was characterized by a green-yellow emission around 500 nm (Fig. 3, Supplementary Fig. 22). Crystals **C**_**α**_ and **C**_**δ**_ gave rise to a green luminescence (*λ*_em_ = ca. 480 nm) when subject to excitation at 365 nm using a handheld ultraviolet lamp. This emission is similar to that produced by perylene in solution at high concentrations (e.g., 10 mM in THF/CH_3_CN (1/1, *v*/*v*)) (Supplementary Fig. 21). This similarity leads us to propose that in the co-crystalline solid states, ***D***_**4d**_**-CDMB-8** serves to disperse the perylene molecules thus maintaining them in monomeric form. The fluorescence emission peak (*λ*_em_), quantum yields (Φ_f_), and fluorescent lifetimes (*τ*_f_) of **C**_**α**_, **A**_**m**_, **M**_**α**_, **M**_**β**_, **D**_**Py**_, and **C**_**δ**_, are summarized (see Table 1, and Supplementary Figs. 44, 45).

**Table 1:** Photophysical properties of various solid materials.

Given the clear distinction in the luminescence features of the different solid forms, the emission colours and spectra could be used to follow the structural changes associated with the conversion between the co-crystalline and mixed crystalline forms. Thus, these spectral changes could be used to monitor exposure to organic solvents. For instance, in a reflection of the complex time-dependent structural changes produced when **C**_**α**_ was exposed to nitrobenzene vapour, the emission of **C**_**α**_ at 482 nm was found to be quenched initially (a finding correlated with the proposed initial formation of **I**_**β**_), followed by the production of an aggregation emission of **Py** ascribed to **D**_**Py**_ as complete conversion from **C**_**α**_ to **M**_**α**_ occurs (Supplementary Figs. 28 and 30).

### Dynamic control over molecular motion and associated colour tuning

**Fig. 4:**
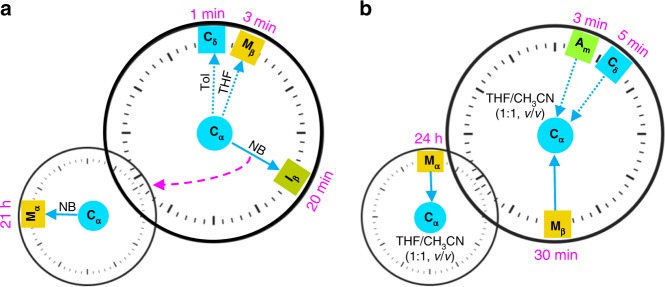
Time-dependent transformation between various solid materials induced by organic solvents. **a** Time-dependent conversion of **C**_**α**_ to **C**_**δ**_, **M**_**β**_, or **I**_**β**_ and then to **M**_**α**_ seen upon exposure to different organic vapours. **b** Time-dependence of the regeneration of **C**_**α**_ from different solid forms, namely **A**_**m**_, **C**_**δ**_, **M**_**β**_, and **M**_**α**_, triggered by exposure to the same THF/CH_3_CN (1/1, *v*/*v*) vapour mixture.

As highlighted by the conversion from **C**_**α**_ to **M**_**α**_ induced by nitrobenzene, the organic vapour-induced changes in the luminescent features of the various solid forms generated from ***D***_**4d**_**-CDMB-8** and **Py** proved time-dependent. The temporal response was found to be a function of both the species in question and the organic solvent employed (Fig. 4). For instance, the conversion of **C**_**α**_ to **C**_**δ**_, **M**_**β**_, or **I**_**β**_ and then to **M**_**α**_ required 1, 3, and 20 min followed by 21 h upon exposure to toluene, THF, and nitrobenzene vapour, respectively (Fig. 4a). Likewise, the regeneration of **C**_**α**_ from **A**_**m**_, **C**_**δ**_, **M**_**β**,_ or **M**_**α**_ via treatment with an identical THF/CH_3_CN (1/1, *v*/*v*) vapour mixture, required 3 min, 5 min, 30 min, and 24 h, respectively (Fig. 4b). Further inter-species transformations are summarized in Supplementary Fig. 41.

The solvent-based differentiation is ascribed to relative affinities for the ***D***_**4d**_**-CDMB-8** host, as compared to **Py**, in the solid state. When the interaction between ***D***_**4d**_**-CDMB-8** and the organic solvent is strong, as true in the case of, e.g., THF and nitrobenzene, decomposition of the co-crystalline species comprising **Py** and ***D***_**4d**_**-CDMB-8** occurs. The net result is formation of mixed crystalline materials containing ***D***_**4d**_**-CDMB**-8⊃solvent and **D**_**Py**_. Such solid forms are characterized by an orange **D**_**Py**_ emission (*λ*_em_ = 590 nm). In contrast, treatment with an organic species (e.g., toluene) with weaker affinity for ***D***_**4d**_**-CDMB-8** (compared with **Py**) only gives rise to a modified co-crystalline ***D***_**4d**_**-CDMB**-8⊃**Py**•solvent species (solvent = toluene). Such forms maintain the monomeric **Py** blue emission (*λ*_em_ = 480 nm).

### 4D code system

**Fig. 5:**
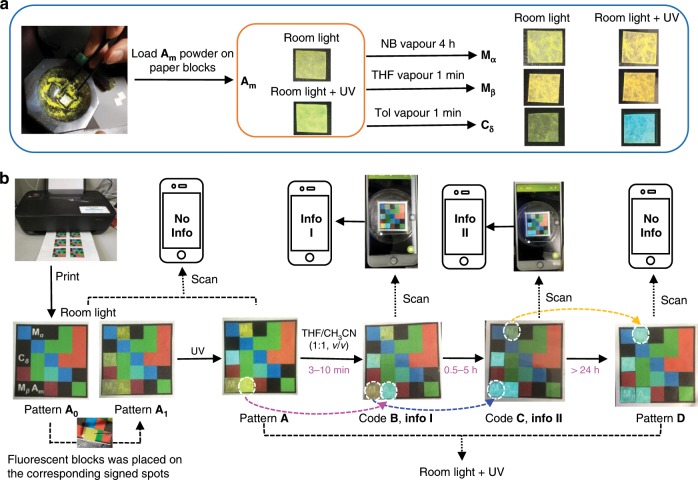
Schematic representation of a 4D code information system based on a time-dependent organic vapour-based response. **a** Preparation of code blocks **A**_**m**_, **M**_**α**_, **M**_**β**_, and **C**_**δ**_. **b** Transformation and masking mad possible by using (i) an original printed colour pattern, (ii) room and/or UV light, and (iii) the time-dependent organic vapour-induced changes in the luminescent features of the constituent code blocks, **A**_**m**_, **M**_**α**_, **M**_**β**_, and **C**_**δ**_. Additional photographs of the various patterns are included in the Supporting Information.

The structural changes and associated diagnostic luminescent features produced via the various solid forms upon exposure to organic solvent vapours were found to proceed on different, but highly reproducible time scales (Supplementary Figs. 41–46). These differences and the temporal fidelity associated with the organic vapour-induced interconversions between **C**_**α**_, **A**_**m**_, **I**_**β**_, **M**_**α**_, **M**_**β**_, and **C**_**δ**_, led us to consider that solid forms generated from ***D***_**4d**_**-CDMB-8** and **Py** could be used to create a time-dependent dynamic 4D code system. As an initial test of this proposition, **A**_**m**_ was loaded onto scraps of paper (0.5 × 0.5 cm) to provide a first fluorescent block. Independent treatment with nitrobenzene, THF, and toluene vapour was then used to generate another three fluorescent blocks, namely **M**_**α**_, **M**_**β**_, and **C**_**δ**_ (Fig. 5a). A printed colour pattern **A**_**0**_ was then generated by means of a commercial colour printer (Fig. 5b). Fluorescent blocks containing **A**_**m**_, **M**_**α**_, **M**_**β**_, and **C**_**δ**_ were then added into the printed pattern to obtain a 3D colour-based pattern (**A**_**1**_). Pattern **A**_**1**_ was found to produce fluorescent pattern **A** when exposed to UV light at 365 nm using an ultraviolet lamp under otherwise normal laboratory conditions (Supplementary Movie 3). Exposing pattern **A** to THF/CH_3_CN (1/1, *v*/*v*) vapour produces patterns **B-D** over different time scales, namely 3–10 min, 0.5–5 h, and greater than 24 h, respectively (cf., Supplementary Movie 4).

To demonstrate the power of this time-dependent approach to coding, we programmed information sets **I** and **II** within the time-dependent patterns **B** and **C**, respectively. The information in question could be accessed directly by scanning these two codes by means of a smart phone. However, different delay times, namely 3–10 min and 0.5–5 h, were required to read out the information (Supplementary Movie 4). As the result of this time-dependent feature, a 3D code system (a planar coloured array) is transformed into a dynamic information coding system characterized by 4D complexity. The net result is a set of code systems with a high degree of inherent confidentiality. This reflects the fact that (i) the different code arrangements (placement of coloured building blocks), (ii) the light source (UV vs Vis), (iii) choice of organic vapour, and (iv) the exposure and monitoring time scales may be used to generate various codes that allow information to be read out in a temporally defined manner. Treating the original pattern **A** with other vapours or set in air was found to give rise to other patterns (**E**–**J**) characterized by different formation lifetimes (see Supplementary Figs. 47–49).

## Discussion

We have shown that molecular motion can be induced by exposure of solid-state host-guest complexes to appropriately chosen organic solvents and that the resulting changes in structure give rise to distinct luminescent features. In the case of the present materials, which are based on the all-hydrocarbon components ***D***_**4d**_**-CDMB-8** and **Py**, the underlying molecular scale motion and the associated changes in the solid-state structures was inferred from a combination of single crystal X-ray diffraction analyses and PXRD studies. A tuneable temporal response, which proved to be a function of both the solid form employed and the chosen organic solvent, was seen. This allowed a dynamic 4D code library to be developed wherein multiple independent keys, including time and a specific organic vapour, are needed in order to read out pre-programmed information.

## Methods

### General considerations

Deuterated solvents were purchased from Cambridge Isotope Laboratory (Andover, MA). All other solvents and reagents were purchased commercially (Aldrich, Acros, or Fisher) and used without further purification. NMR spectra were recorded on Bruker AVANCE III 500WB, AVANCE 400, or 400JNM-ECZ400S spectrometers. The ^1^H NMR chemical shifts are referenced to residual solvent signals (tetrachloroethane-*d*_2_ (TCE-*d*_2_): *δ*_H_ = 5.95 ppm, *δ*_C_ = 74.10 ppm. CDCl_3_: *δ*_H_ *=* 7.26 ppm. THF-*d*_8_: *δ*_H_ *=* 1.75, 3.60 ppm. CH_3_CN-*d*_3_: *δ*_H_ *=* 1.94 ppm). UV–vis spectra were collected on a Shimadzu UV-2450 instrument. Fluorescence emission spectra and lifetimes (*τ*_f_) were collected on an Edinburgh Instruments FS5 spectrometer. Fluorescence quantum yields (Φ_f_) were obtained using a HAMAMATSU Quantaurus-QY instrument. PXRD studies were carried out using a Shimadzu XRD-7000 setup. Pictures and movies were recorded on a smart phone (Vivo X23) or an industrial digital camera (E3ISPM05000KPA) linked to a stereo microscope (Carton SPZT-50PFM). The information codes were registered on www.colorzip.com and read by a smart phones (iPhone 5S) using the COLORCODE^TM^ app, which at the time the work was performed could be downloaded for free from the Apple app store.

### Single crystal X-ray diffractions

Unless otherwise noted, single crystals used to obtain the X-ray diffraction structures reported in the main text grew as yellow prisms, blocks, or colourless prisms. The data crystals used for single crystal analyses were cut from clusters of the corresponding crystals. The data were collected on Saturn724 + (2 × 2 bin mode) or SuperNova, Dual, Cu at Home/Near, AtlasS2 diffractometers. Data reduction was performed using the CrystalClear (Rigaku Inc., 2007) or CrysAlisPro 1.171.39.32a (Rigaku OD, 2017) software packages. The structures were refined by full–matrix least–squares on F^2^ with anisotropic displacement parameters for the non–H atoms using SHELXL-2014^47^. The hydrogen atoms were calculated in idealized positions with isotropic displacement parameters set to 1.2 × Ueq of the attached atom (1.5 × Ueq for methyl hydrogen atoms). Definitions used for calculating R(F), Rw(F2) and the goodness of fit, S, are given in Supplementary Tables 1, 2. Neutral atom scattering factors and values used to calculate the linear absorption coefficient are from the International Tables for X-ray Crystallography (1992)^48^. All ellipsoid figures were generated using SHELXTL/PC^49^.

### Synthesis of CDMB-8

Under an argon atmosphere, a mixture of **1** (600 mg, 0.88 mmol), **2** (500 mg, 0.88 mmol), Pd(dppf)_2_Cl_2_·CH_2_Cl_2_ (148 mg, 0.18 mmol), Cs_2_CO_3_ (14.4 g, 44 mmol), and 1000 mL deaerated toluene were added to a 2 L three-necked round-bottomed flask. The reaction mixture was heated under reflux for 12 h. After allowing to cool to room temperature, the volatiles were removed using a rotary evaporator. The resulting residue was dissolved in 100 mL CH_2_Cl_2_, then filtered through a short neutral alumina column and washed with CH_2_Cl_2_ (50 mL). The volatiles (primarily CH_2_Cl_2_) were removed from the filtrate via rotary evaporation. The resulting residue was dissolved in 50 mL cyclohexane and purified by a neutral alumina column using cyclohexane as the eluent to give analytically pure ***C***_**s**_**-CDMB-8** and ***D***_**4d**_**-CDMB-8** as a white solid 110 mg (15%) and 180 mg (25%), respectively. ***C***_**s**_**-CDMB-8**: ^1^H NMR (500 MHz, TCE-*d*_2_, 278 K) *δ* (ppm): 7.08 (s, 2H), 7.04 (s, 2H), 7.02 (s, 2H), 7.01 (s, 1H), 6.96 (s, 1H), 6.91 (s, 1H), 6.89 (s, 2H), 6.86 (s, 2H), 6.83 (s, 2H), 6.81 (s, 2H), 2.16 (s, 6H), 2.12(s, 6H), 2.11 (s, 6H), 2.09 (s, 6H), 2.08 (s, 6H), 2.07 (s, 12H), 2.02 (s, 6H). ***D***_**4d**_**-CDMB-8**: ^1^H NMR (500 MHz, TCE-*d*_2_, 278 K) *δ* (ppm): 7.03 (s, 8H), 6.75 (s, 8H), 2.01 (s, 48H). Both products were further characterized by single crystal X-ray diffraction analysis (Supplementary Fig. 1).

### Preparation of co-crystal materials

Subjecting mixtures of ***D***_**4d**_**-CDMB-8** (1.00 mM), and 1 molar equiv. of perylene in THF/CH_3_CN (1/1, *v/v*) or toluene (Tol) to slow evaporation resulted in the formation of single crystals of [(***D***_**4d**_**-CDMB-8**)_2_⊃(**Py**•6CH_3_CN)•**Py**•2THF] (**C**_**α**_) or [(***D***_**4d**_**-CDMB-8**)_2_⊃(**Py**)_2_•3Tol] (**C**_**δ**_), respectively. Separately, single crystals of [***D***_**4d**_**-CDMB-8**⊃(NB)_2_•2NB] (**C**_**β**_) were obtained via the slow evaporation of ***D***_**4d**_**-CDMB-8** (1.00 mM) in nitrobenzene (NB), or by dissolving **C**_**α**_ in nitrobenzene and subjecting the resulting solution to slow evaporation. These various single crystals were analyzed by X-ray diffraction methods.

### Organic solvent vapour treatment equipment and conditions

Small borosilicate glass fragments (thickness: 0.3 cm) were placed in a 100 mL petri dish, and a borosilicate glass plate (4 × 4 × 0.3 cm) was stacked up on these borosilicate glass fragments. The test materials were loaded on a small borosilicate glass block (0.5 × 0.5 × 0.1 cm) and set in the centre of the glass plate. Two millilitres of organic solvents liquid were dropwise added to the bottom of the petri dish, which was immediately covered with the lid (Supplementary Fig. 14). Note: These conditions provide the organic solvent vapours in near-saturated form in air at room temperature (298 K).

### In situ time-dependent emission spectra collection

Briefly, the test material is loaded on a paper square (0.5 × 0.5 × 0.1 cm). The paper square is fixed on a quartz plate (3 × 1 × 0.1 cm) by means of a copper wire. The quartz plate is then placed in a 20 mL borosilicate glass bottle and set on the sample stage of an Edinburgh Instruments FS5 instrument. The excitation light (365 nm) is then focused on the sample and the spectra features recorded. At this juncture, 0.5 mL of the organic solvent in question was dropped in liquid form onto the bottom of the glass bottle with an injector (Supplementary Fig. 15). The setup was then covered with the lid immediately (without tightening so as to allow venting to the atmosphere. these conditions provide the organic solvent vapours that are essentially saturated in air at room temperature (298 K)). The emission spectra were recorded as a function of time after the organic solvent in question was added.

### The equipment and conditions for 4D code generated

A small glass fragment (thickness: 0.3 cm) was placed in a 100 mL petri dish, and a borosilicate glass plate (4 × 4 × 0.3 cm) was stacked up on theses glass fragments. Predetermined patterns were set on the glass plate, 2 mL of the organic solvent in question (in liquid form) was are added to the bottom of petri dish, which was immediately covered with the lid (these conditions provide the organic solvent vapours in near saturated form in air and at room temperature (298 K)) (Supplementary Fig. 50). Pictures and movies were recorded under natural with/without UV light (using a commercial ultraviolet lamp (365 nm)). Movies showing the conversion of pattern **A**_**0**_ to pattern **D** upon treatment with THF/CH_3_CN (1/1, *v*/*v*) vapour are given in Supplementary Movies 3, 4.

## Supplementary information

Supplementary Information

Peer Review File

Description of Additional Supplementary Files

Supplementary Movie 1

Supplementary Movie 2

Supplementary Movie 3

Supplementary Movie 4

## Data Availability

The X-ray crystallographic coordinates for structures reported in this study have been deposited at the Cambridge Crystallographic Data Centre (CCDC), under deposition numbers 1859991, 1859999, 1937315, 1937316, and 1937317. These data can be obtained free of charge from The Cambridge Crystallographic Data Centre via www.ccdc.cam.ac.uk/data_request/cif. And all other data supporting the findings of this study are available from the article and its Supplementary Information files or available from the corresponding authors upon reasonable request.

## Code availability

The scan software used in the present study is COLORCODE^®^ (http://colorzip.com). At the time of publication this information reading application was available free of charge for use in code scanning, but not to the authors’ knowledge for any other purpose.

## References

[1] Zhu L, Liu X-Q, Jiang H-L, Sun L-B (2017). Metal-organic frameworks for heterogeneous basic catalysis. Chem. Rev..

[2] Formenti D, Ferretti F, Scharnagl FK, Beller M (2019). Reduction of nitro compounds using 3d-non-noble metal catalysts. Chem. Rev..

[3] Diercks CS, Liu Y, Cordova KE, Yaghi OM (2018). The role of reticular chemistry in the design of CO2 reduction catalysts. Nat. Mater..

[4] Wang X (2018). Sulfone-containing covalent organic frameworks for photocatalytic hydrogen evolution from water. Nat. Chem..

[5] Schoedel A, Ji Z, Yaghi OM (2016). The role of metal–organic frameworks in a carbon-neutral energy cycle. Nat. Energy.

[6] Yu J (2017). CO2 capture and separations using MOFs: computational and experimental Studies. Chem. Rev..

[7] Kumar KV, Preuss K, Titirici M-M, Rodriguez-Reinoso F (2017). Nanoporous materials for the onboard storage of natural gas. Chem. Rev..

[8] Ding M, Flaig RW, Jiang H-L, Yaghi OM (2019). Carbon capture and conversion using metal-organic frameworks and MOF-based materials. Chem. Soc. Rev..

[9] Naumov P, Chizhik S, Panda MK, Nath NK, Boldyreva E (2015). Mechanically responsive molecular crystals. Chem. Rev..

[10] Huang R-W (2017). Hypersensitive dual−function luminescence switching of a silver-chalcogenolate cluster-based metal-organic framework. Nat. Chem..

[11] Wang L, Li Q (2018). Photochromism into nanosystems: towards lighting up the future nanoworld. Chem. Soc. Rev..

[12] Alam P (2019). Spontaneous and fast molecular motion at room temperature in the solid state. Angew. Chem. Int. Ed..

[13] Ryabchun A, Li Q, Lancia F, Aprahamian I, Katsonis N (2019). Shape-persistent actuators from hydrazone photoswitches. J. Am. Chem. Soc..

[14] Deng H, Olson MA, Stoddart JF, Yaghi OM (2010). Robust dynamics. Nat. Chem..

[15] Vogelsberg CS, Garcia-Garibay MA (2012). Crystalline molecular machines: function, phase order, dimensionality, and composition. Chem. Soc. Rev..

[16] Jiang X, Rodríguez-Molina B, Nazarian N, Garcia-Garibay MA (2014). Rotation of a bulky triptycene in the solid state: toward engineered nanoscale artificial molecular machines. J. Am. Chem. Soc..

[17] Vukotic VN, Harris KJ, Zhu K, Schurko RW, Loeb SJ (2012). Metal–organic frameworks with dynamic interlocked components. Nat. Chem..

[18] Vukotic VN (2015). Mechanically interlocked linkers inside metal-organic frameworks: effect of ring size on rotational dynamics. J. Am. Chem. Soc..

[19] Matsuno T, Nakai Y, Sato S, Maniwa Y, Isobe H (2018). Ratchet-free solid-state inertial rotation of a guest ball in a tight tubular host. Nat. Commun..

[20] Matsuno, T., Fukunaga, K., Sato, S. & Isobe, H. Retarded solid-state rotations of an oval-shaped guest in a deformed cylinder with CH–π arrays. *Angew. Chem. Int. Ed.* **58**, 12170–12174 (2019). 10.1002/anie.201907040 31270917

[21] Zhu K, O’Keefe CA, Vukotic VN, Schurko RW, Loeb SJ (2015). A molecular shuttle that operates inside a metal-organic framework. Nat. Chem..

[22] Wang H (2017). Bending, curling, rolling, and salient behavior of molecular crystals driven by [2+2] cycloaddition of a styrylbenzoxazole derivative. Angew. Chem. Int. Ed..

[23] Gelebart AH (2017). Making waves in a photoactive polymer film. Nature.

[24] Chen P-Z (2017). A solid-state fluorescent material based on carbazole-containing difluoroboron β-diketonate: multiple chromisms, the self-assembly behavior, and optical waveguides. Adv. Funct. Mater..

[25] Chang J, Sheng L, Wei T, Fan Z (2018). Molecular diffusion-driven motion in 2D graphene film. Adv. Funct. Mater..

[26] Sun Y, Lei Y, Dong H, Zhen Y, Hu W (2018). Solvatomechanical bending of organic charge transfer cocrystal. J. Am. Chem. Soc..

[27] Athiyarath V, Sureshan KM (2019). Spontaneous single-crystal-to-single-crystal evolution of two cross-laminated polymers. Angew. Chem. Int. Ed..

[28] Peng H-Q (2019). Visualizing the initial step of self-assembly and the phase transition by stereogenic amphiphiles with aggregation-induced emission. ACS Nano.

[29] Zhao Z (2019). Highly efficient photothermal nanoagent achieved by harvesting energy via excited-state intramolecular motion within nanoparticles. Nat. Commun..

[30] Qu D-H, Wang Q-C, Zhang Q-W, Ma X, Tian H (2015). Photoresponsive host−guest functional Systems. Chem. Rev..

[31] Yu G, Jie K, Huang F (2015). Supramolecular amphiphiles based on host−guest molecular recognition motifs. Chem. Rev..

[32] Juríček M (2014). Induced–fit catalysis of corannulene bowl-to-bowl inversion. Nat. Chem..

[33] Sauvage J-P, Dietrich-Buchecker C (1999). Molecular Catenanes, Rotaxanes and Knots..

[34] Erbas-Cakmak S (2017). Rotary and linear molecular motors driven by pulses of a chemical fuel. Science.

[35] Atwood, J., Gokel, G. W. & Barbour, L. *Comprehensive Supramolecular Chemistry Ii* (Elsevier, 2017).

[36] Goodby, J. et al. *Handbook of Liquid Crystals, 2nd edn*. (Wiley-VCH, Weinheim, 2014).

[37] Tschierske C (2013). Development of structural complexity by liquid-crystal self-assembly. Angew. Chem. Int. Ed..

[38] Goossens K, Lava K, Bielawski CW, Binnemans K (2016). Ionic liquid crystals: versatile materials. Chem. Rev..

[39] Mark, H. F. *Encyclopedia of Polymer Science and Technology, 2nd edn*. (John Wiley & Sons, Inc., Hoboken, 2002).

[40] Meesorn W, Calvino C, Natterodt JC, Zoppe JO, Weder C (2019). Bio-inspired, self-toughening polymers enabled by plasticizer-releasing microcapsules. Adv. Mater..

[41] Arias-Zapata J (2016). Ultrafast assembly of PS-PDMS block copolymers on 300 mm wafers by blending with plasticizers. Adv. Funct. Mater..

[42] Kumar V, Kim K-H, Kumar P, Jeon B-H, Kim J-C (2017). Functional hybrid nanostructure materials: Advanced strategies for sensing applications toward volatile organic compounds. Coord. Chem. Rev..

[43] Lustig WP (2017). Metal-organic frameworks: functional luminescent and photonic materials for sensing applications. Chem. Soc. Rev..

[44] Jin M, Sumitani T, Sato H, Seki H, Ito H (2018). Mechanical-stimulation-triggered and solvent-vapor-induced reverse single-crystal-to-single-crystal phase transitions with alterations of the luminescence color. J. Am. Chem. Soc..

[45] Li Y (2017). Supramolecular self-assembly and dual-switch vapochromic, vapoluminescent, and resistive memory behaviors of amphiphilic platinum (II) complexes. J. Am. Chem. Soc..

[46] Yang Y-D, Gong H-Y (2019). Thermally activated isomeric all-hydrocarbon molecular receptors for fullerene separation. Chem. Commun..

[47] Sheldrick GM (2015). SHELXT–Integrated space-group and crystal-structure determination. Acta Cryst. A..

[48] Wilson, A. J. C. *International Tables for X-ray Crystallography. Vol. C, Tables 4.2.6.8 and 6.1.1.4* (Kluwer Academic Press, 1992).

[49] Sheldrick, G. M. *SHELXTL/PC (Version 5.03)* (Siemens Analytical X-ray Instruments, Inc., Wisconsin, 1994).

