# Supplementary material for: Time-dependent solid-state molecular motion and colour tuning of host-guest systems by organic solvents
Source: Nat Commun. 2020 Jan 7;11:77. doi: 10.1038/s41467-019-13844-5 (PMC6946670; doi:10.1038/s41467-019-13844-5)
Supplement: Supplementary file 1 — Supplementary Information [file 41467_2019_13844_MOESM1_ESM.pdf]

Supplementary Information For:

Time-dependent solid-state molecular motion and colour tuning of host-guest systems by organic solvents

Yu-Dong Yang,[1] Xiaofan Ji,[2] Zhi-Hao Lu,[1] Jian Yang,[1] Chao Gao,[1] Haoke Zhang,[2] Ben Zhong Tang,[2]* Jonathan L. Sessler,[3, 4]* Han-Yuan Gong[1]*

[1]College of Chemistry, Beijing Normal University, Beijing, Xinjiekouwaidajie 19, 100875, P. R. China.

[2]Department of Chemistry, HKUST Jockey Club Institute for Advanced Study, Institute of Molecular Functional Materials, Division of Biomedical Engineering, State Key Laboratory of Molecular Neuroscience, Division of Life Science, The Hong Kong University of Science and Technology, Clear Water Bay, Kowloon, Hong Kong, China.

[3]Department of Chemistry, The University of Texas at Austin, 105 East 24th Street, Stop A5300, Austin, TX 78712-1224, USA.

[4]Department of Chemistry and Center for Supramolecular Chemistry and Catalysis, Shanghai University, 99 Shangda Road, Shanghai 200444, China.

Author e-mail address: hanyuangong@bnu.edu.cn, sessler@cm.utexas.edu, tangbenz@ust.hk.

Materials and Methods

Section S1: Synthesis of **CDMB-8**

Section S2: Host/guest interactions between **CDMB-8** and perylene in solution

Section S3: Single crystal X-ray diffraction studies of the complexes formed between ***D_{4d}*-CDMB-8**, perylene, and organic solvents

Section S4: Organic solvent vapour promoted solid state molecular motion and related structural and emission response studies of materials formed between ***D_{4d}*-CDMB-8**, perylene, and organic solvents

Section S5: Four dimension information coding

Section S1: Synthesis of **CDMB-8**

The improved synthesis of isomeric **CDMB-8** referenced in the main test is shown in Supplementary Fig. 1. Briefly, a change in the reaction medium from CH_3CN to toluene (Tol) led to the production of both of ***C*s-CDMB-8** (15%) and ***D*4d-CDMB-8** (25%) rather than only ***C*s-CDMB-8** (originally obtained in 20% yield)[1]. This finding is ascribed to the toluene acting as a synthesis template for both products as inferred from single crystal X-ray diffraction analyses of **CDMB-8⊃**Tol (Note: Single crystals of [***D*4d-CDMB-8⊃**Tol] and [***C*s-CDMB-8⊃**Tol•$(Tol)_{0.5}$] were grown via slow evaporation of either a toluene solution or a toluene/CH_3CN (1.0 mM) solution; Supplementary Table 1, Supplementary Fig.1. **b1** and, **b2**), as well as the higher reaction temperature that favours formation of the thermodynamic product (***D*4d-CDMB-8**). As noted previously, a heat activation process could be used to convert a mixture containing ***C*s-** and ***D*4d-CDMB-8** to pure ***D*4d-CDMB-8** in quantitative yield.

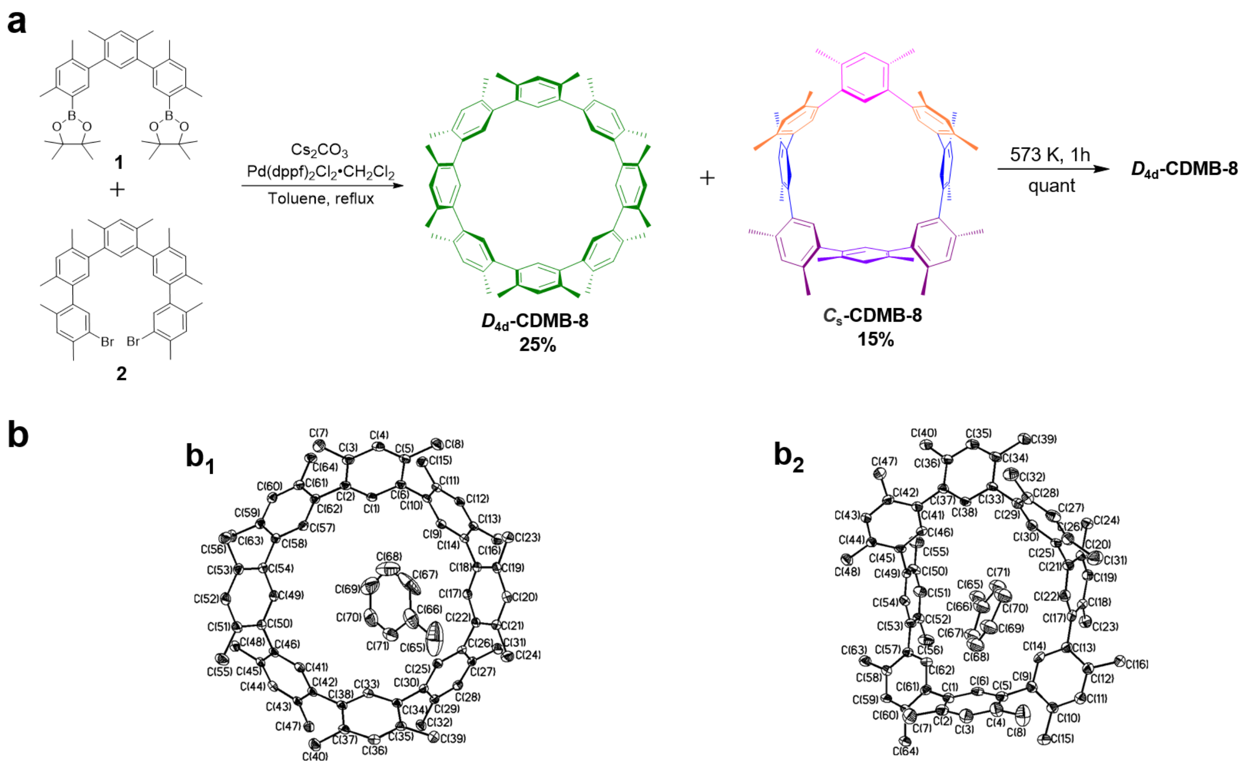

Supplementary Figure 1. **a**, Synthesis of all-hydrocarbon macrocycles ***C*s-** and ***D*4d-CDMB-8**. **b**, Complexes of ***D*4d-** or ***C*s-CDMB-8** containing toluene as seen in the single crystal structures of [***D*4d-CDMB-8⊃**Tol] (**b1**) and [***C*s-CDMB-8⊃**Tol•$(Tol)_{0.5}$] (**b2**), respectively.

Supplementary Table 1. X-ray crystallographic data for [***D*$_{4d}$-CDMB-8**⊐Tol] and [***C*$_s$-CDMB-8**⊐Tol•(Tol)$_{0.5}$]

	[***D*$_{4d}$-CDMB-8**⊐Tol]	[***C*$_s$-CDMB-8**⊐Tol•(Tol)$_{0.5}$]
CCDC No.	1859999	1859991
description	prism	prism
colour	colourless	colourless
from solution	toluene/CH_3CN	toluene/CH_3CN
empirical formula	C_{71} H_{72}	$C_{74.50}$ H_{76}
Mr	925.28	971.35
crystal size (mm^3)	0.10 × 0.06 × 0.04	0.12 × 0.06 × 0.03
crystal system	monoclinic	triclinic
space group	P 21/n	P-1
a [Å]	16.6449(2)	14.209(3)
b [Å]	11.6376(2)	14.451(3)
c [Å]	29.0261(3)	15.052(3)
α [deg]	90	106.88(3)
β [deg]	98.0440(10)	101.52(3)
γ [deg]	90	94.76(3)
V / [Å^3]	5567.23(13)	2865.0(11)
d / [g/cm^3]	1.104	1.126
Z	4	2
T [K]	100.00(10)	173.1500
R1, wR2 $I > 2\sigma(I)$	0.0813, 0.2267	0.1381, 0.2725
R1, wR2 (all data)	0.0896, 0.2354	0.1630, 0.2886
quality of fit	1.009	1.018

Section S2: Host/guest interactions between **CDMB-8** and perylene in solution

It was found that a mixture consisting of ***C*$_s$-** or ***D*$_{4d}$-CDMB-8** and 1 molar equiv. of perylene (**Py**) (final concentrations = 1.00 mM or 0.25 mM in each; THF/CH_3CN = 1/1, *v*/*v*) induced little if any discernible change in the UV-Vis absorption or fluorescence emission spectra (Supplementary Figs. 2 and 3). Moreover, only a small high field chemical shift change (< 2 Hz) in the H(β) resonance of ***D*$_{4d}$-CDMB-8** was observed in the ^{1}H NMR spectrum when perylene was added to a THF-d_8/CH_3CN-d_3 (1/1, *v*/*v*) solution of ***D*$_{4d}$-CDMB** (Supplementary Figs. 4 and 5).

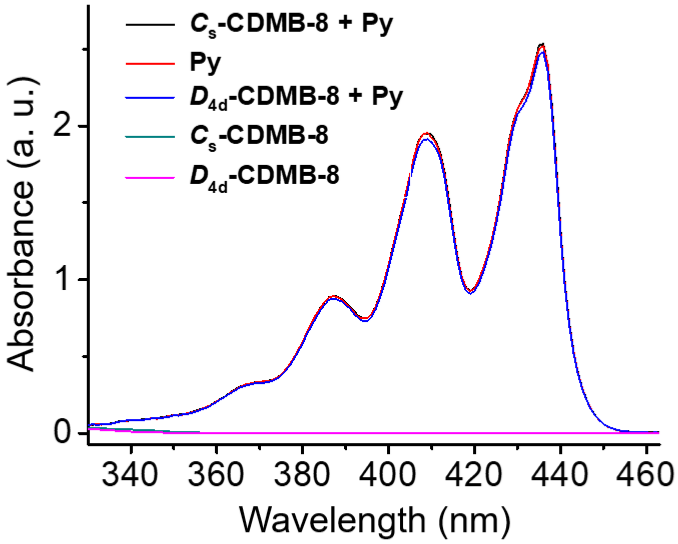

Supplementary Figure 2. UV-vis spectra of ***D***$\boldsymbol{D_{4d}}$**-** or $\boldsymbol{C_s}$**-CDMB-8** (0.25 × 10^{-3} M) in the absence (pink or cyan curves, respectively) and presence (blue or black, respectively) of 1 molar equiv. of **Py**. Also shown is the spectrum of **Py** alone (red curve, 0.25 × 10^{-3} M) recorded in THF/CH_3CN (1/1, *v*/*v*) (1 cm optical path).

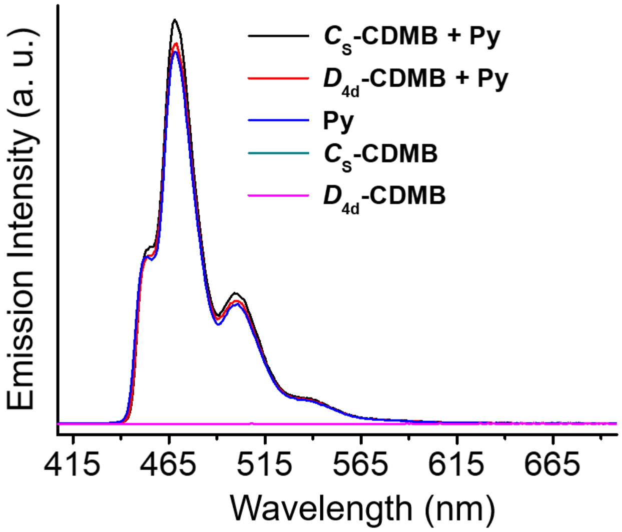

Supplementary Figure 3. Emission spectra of $\boldsymbol{D_{4d}}$**-** or $\boldsymbol{C_s}$**-CDMB-8** (0.25 × 10^{-3} M) recorded in THF/CH_3CN (1/1, *v*/*v*) in the absence (pink or cyan curves, respectively) and presence (red or black, respectively) of 1 molar equiv. of **Py**, also only perylene **Py** (blue curve, 0.25 × 10^{-3} M) (λ_{ex} = 365 nm, voltage = 400 V, entrance slit width = 1 nm, exit slit width = 1 nm).

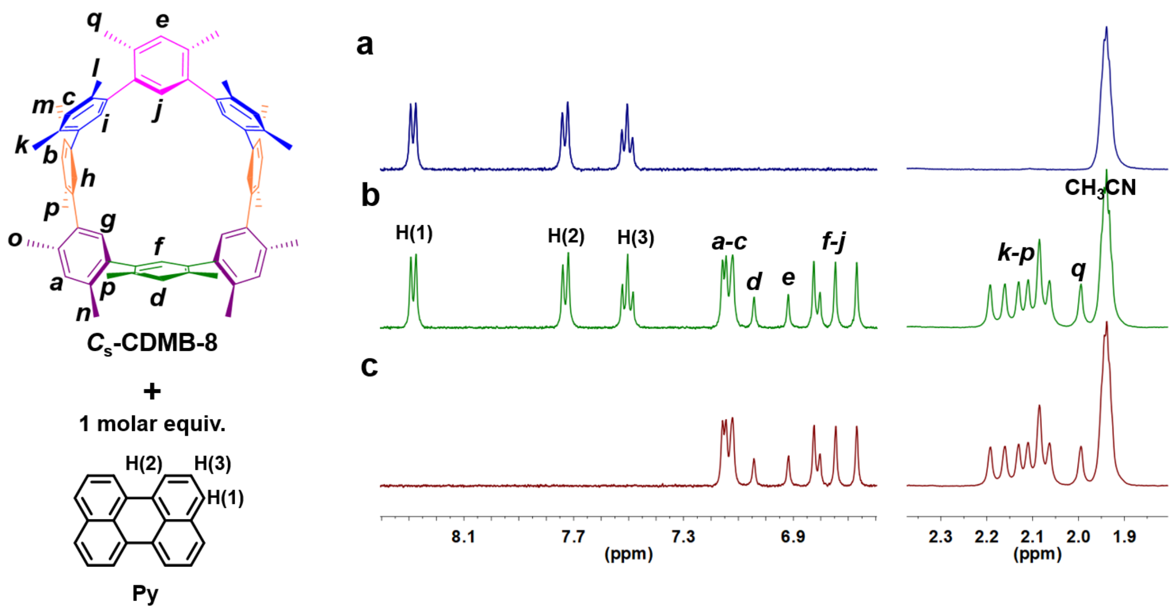

Supplementary Figure 4. Expansion of the ^{1}H NMR spectra of **Py** (1.00×10^{-3} M) (**a**), a mixture of ***C*s-CDMB-8** (1.00×10^{-3} M) and 1 molar equiv. of **Py** (**b**), and ***C*s-CDMB-8** (1.00×10^{-3} M) (**c**) in THF-d_8/CH_3CN-d_3 (1/1, *v*/*v*) at 298 K (400 MHz).

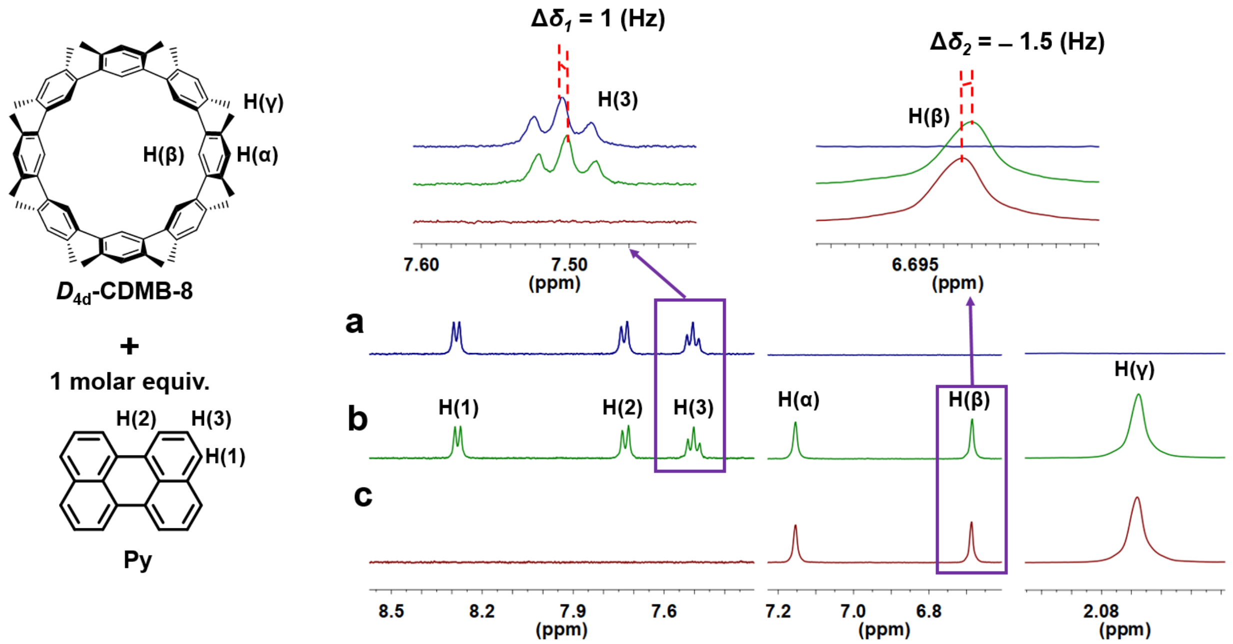

Supplementary Figure 5. Expansion of the ^{1}H NMR spectra of **Py** (1.00×10^{-3} M) (**a**), a mixture of ***D*4d-CDMB-8** (1.00×10^{-3} M) and 1 molar equiv. of **Py** (**b**), and ***D*4d-CDMB-8** (1.00×10^{-3} M) (**c**) in THF-d_8/CH_3CN-d_3 (1/1, *v*/*v*) at 298 K (400 MHz).

Section S3: Single crystal X-ray diffraction studies of the complexes formed from ***D*$_{4d}$-CDMB-8**, perylene, and organic solvents

Supplementary Table 2. X-ray crystallographic data for [(***D*$_{4d}$-CDMB-8**)$_2$⊃(**Py**•6CH$_3$CN)•**Py**•2THF] (**C$_\alpha$**), [***D*$_{4d}$-CDMB-8**⊃(NB)$_2$•2NB] (**C$_\beta$**), and [(***D*$_{4d}$-CDMB-8**)$_2$⊃(**Py**)$_2$•3Tol] (**C$_\delta$**)

	[(***D*$_{4d}$-CDMB-8**)$_2$⊃(**Py**•6CH$_3$CN)•**Py**•2THF]	[***D*$_{4d}$-CDMB-8**⊃(NB)$_2$•2NB]	[(***D*$_{4d}$-CDMB-8**)$_2$⊃(**Py**)$_2$•3Tol]
CCDC No.	1937315	1937317	1937316
description	prism	block	block
colour	yellow	yellow	yellow
from solution	THF/CH$_3$CN	nitrobenzene	toluene
empirical formula	$C_{94}H_{93}N_3O$	$C_{88}H_{84}N_4O_8$	$C_{94.5}H_{88}$
Mr	1280.71	1325.59	1223.64
crystal size (mm^3)	0.18 × 0.06 × 0.04	0.25 × 0.20 × 0.1	0.15 × 0.12 × 0.05
crystal system	monoclinic	triclinic	monoclinic
space group	P 21/n	P -1	P 21/c
a [Å]	15.5489(2)	15.6471(3)	14.7081(3
b [Å]	24.3558(4)	15.7299(3)	19.9521(3)
c [Å]	19.4758(3)	30.2933(6)	24.7253(4)
α [deg]	90.00	89.9310(10)	90.00
β [deg]	100.6790(10)	88.093(2)	105.319(2)
γ [deg]	90.00	82.0980(10)	90.00
V / [Å^3]	7247.86(19)	7381.1(2)	6998.0(2)
d / [g/cm^3]	1.174	1.193	1.161
Z	4	4	4
T [K]	100.01(10)	100.01(10)	100.01(10)
R1, wR2 $I > 2\sigma(I)$	0.1058, 0.2257	0.0719, 0.2024	0.0965, 0.2240
R1, wR2 (all data)	0.1227, 0.2357	0.0795, 0.2107	0.1056, 0.2312
quality of fit	1.027	1.023	1.059

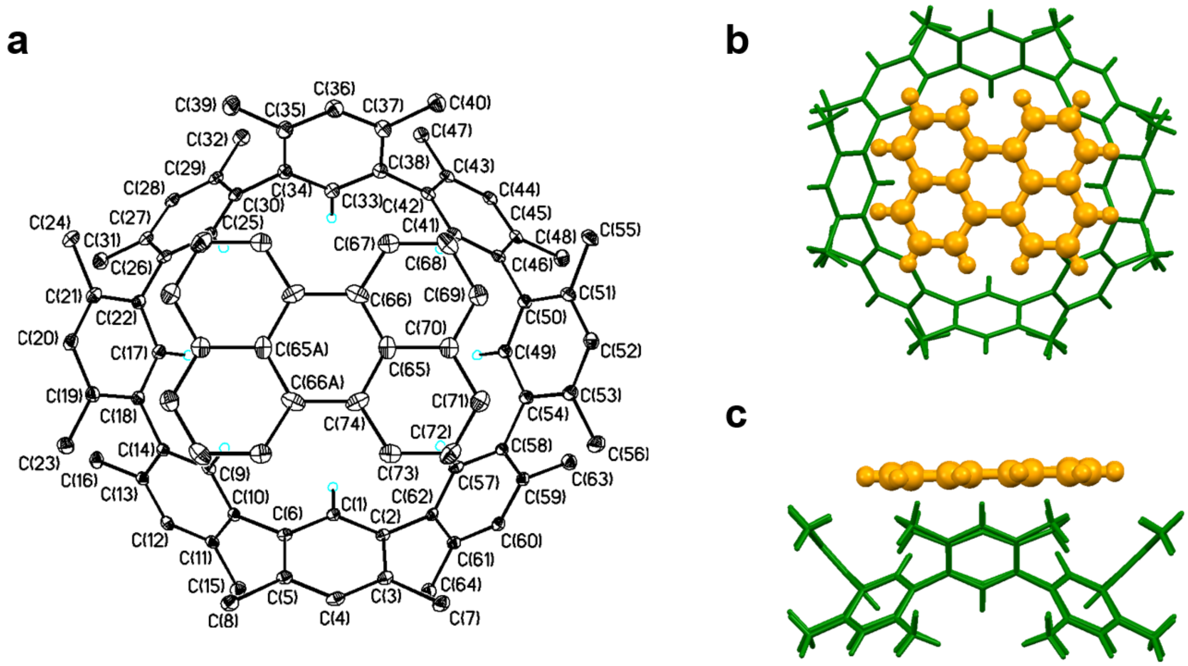

Supplementary Figure 6. **a**, Top view in ellipsoid form showing the binding interactions between ***D*$_{4d}$-CDMB-8** C(1) and **Py** C(65) as observed in the single crystal structure of [(***D*$_{4d}$-CDMB-8**)$_2$⊃(**Py**•6CH_3CN)•**Py**•2THF] (**C**$_\alpha$). **b** and **c**, Structure shown as a top view and side view in stick form. Displacement ellipsoids are scaled to the 25% probability level. All the other molecules and atoms have been omitted for clarity. Possible CH-π interactions are inferred from the following selected distances [Å]: C(9)---C(68A) 3.730(8), C(57)---C(72) 3.639(6), C(41)---C(68) 3.721(8), C(25)---C(72A) 3.807(0). Possible π-π donor-acceptor interactions are inferred from the following selected interatomic distances [Å]: C(69)---C(51) 3.575(3), C(69)---C(52) 3.673(3), C(71)---C(53) 3.569(1), C(69A)---C(19) 3.479(8), C(71A)---C(20) 3.537(9), C(71A)---C(21) 3.474(1).

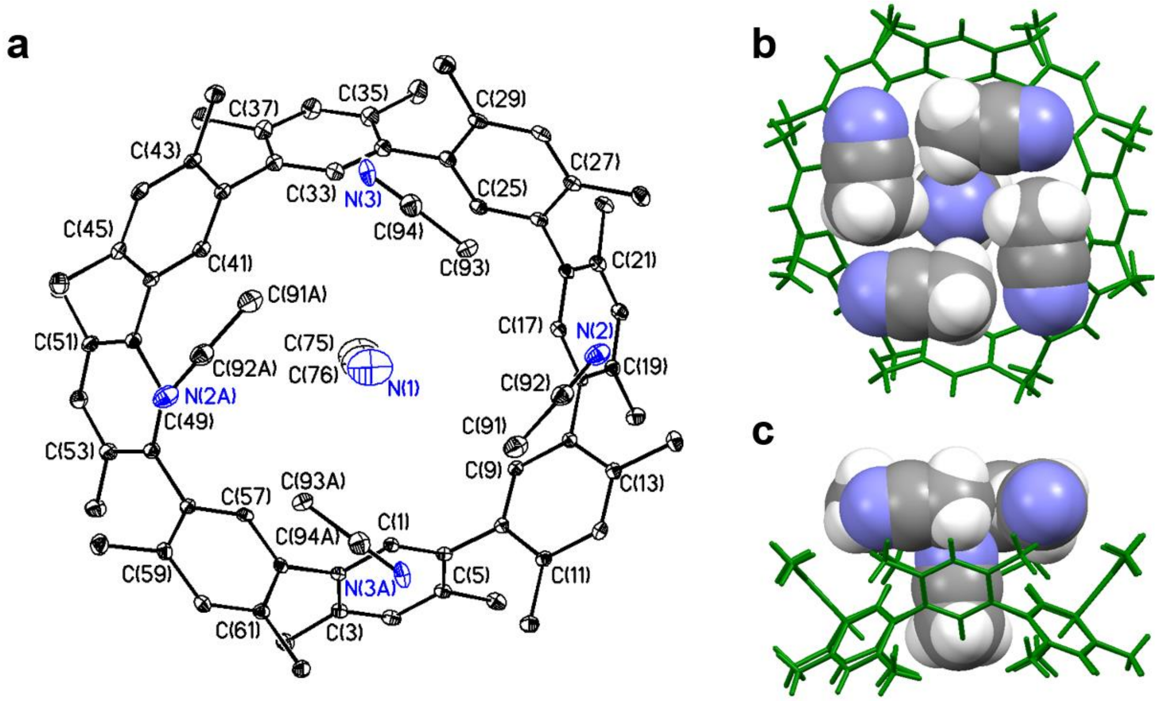

Supplementary Figure 7. **a**, Top view in ellipsoid form showing the binding interactions between ***D*$_{4d}$-CDMB-8** C(1) and N(1), N(2), N(3), N(2A), and N(3A) of the acetonitrile guest as observed in the single crystal structure of [(***D*$_{4d}$-CDMB-8**)$_2$⊃(**Py**•6CH_3CN)•**Py**•2THF] (**C**$_{\alpha}$). **b** and **c**, Structure shown as a top and side views in stick form. Displacement ellipsoids are scaled to the 25% probability level. All the other molecules and atoms have been omitted for clarity. Possible CH-π interactions are inferred from the following selected distances [Å]: C(93)---N(2) 3.466(6), C(91)---N(3) 3.471(5), C(93)---N(2) 3.466(6), C(91)---N(3) 3.471(5), C(91)---N(1) 3.691(5), C(93)---N(1) 3.616(5), C(91---N(1) 3.608(5), C(93)---N(1) 3.558(2), C(93)---C(60) 3.655(3), C(91)---C(44) 3.725(7), C(93)---C(28) 3.786(7), C(91)---C(12) 3.661(9). Possible π-π donor-acceptor interactions are inferred from the following selected interatomic distances [Å]: C(94)---C(29) 3.712(7), C(92)---C(45) 3.622(9), C(94)---C(61) 3.584(0), C(92)---C(13) 3.642(5).

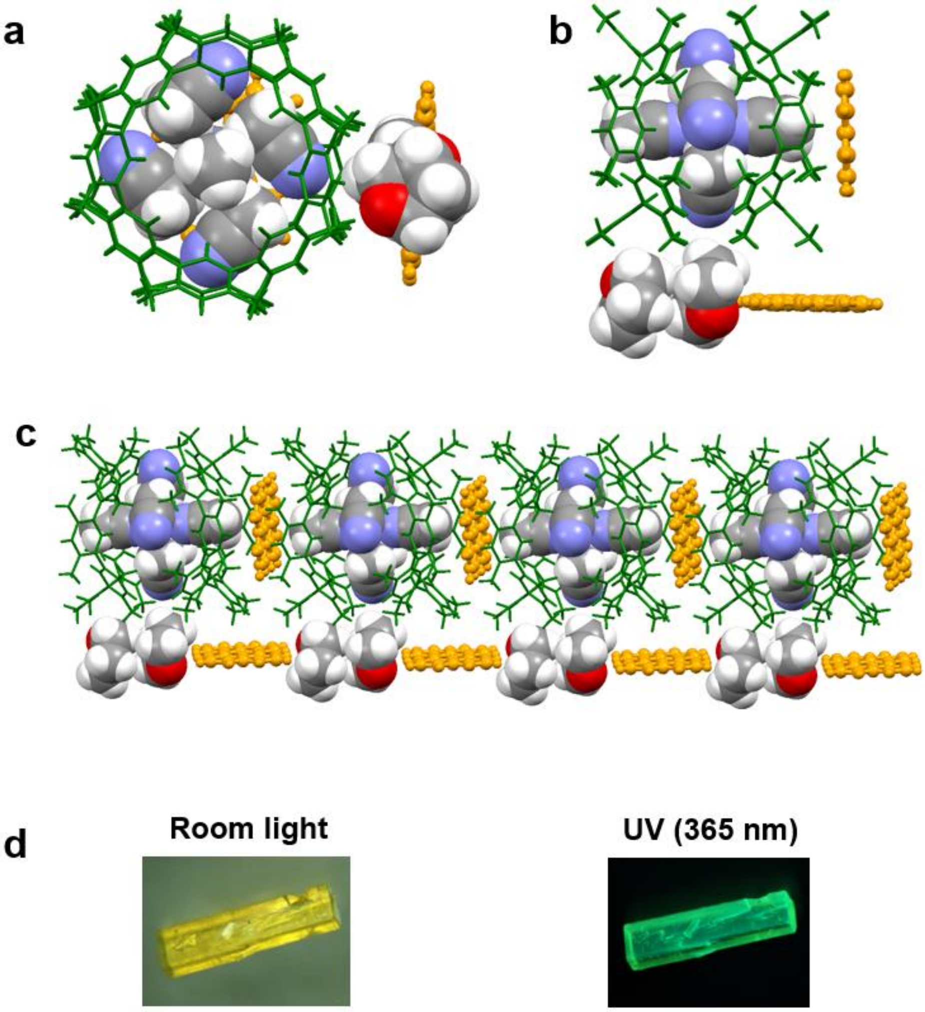

Supplementary Figure 8. **a** and **b**, Top and side views of the periodic repeat unit (***D_{4d}*-CDMB-8**)$_2$⊃(**Py**•6CH_3CN)•**Py**•2THF seen in the solid state. **c**, 1D packing structure seen within single crystals of [(***D_{4d}*-CDMB-8**)$_2$⊃(**Py**•6CH_3CN)•**Py**•2THF] (**$C_α$**). **d**, photographs of single crystals **$C_α$** under normal laboratory light and using a commercial ultraviolet lamp (365 nm).

It is noted that the co-crystalline material **$C_α$** could be easily prepared on gram scale by adding CH_3CN in isochoric fashion into a solution containing ***D_{4d}*-CDMB-8** (5.00 mM) and 1 molar equiv. of perylene in THF (Supplementary Movie 1).

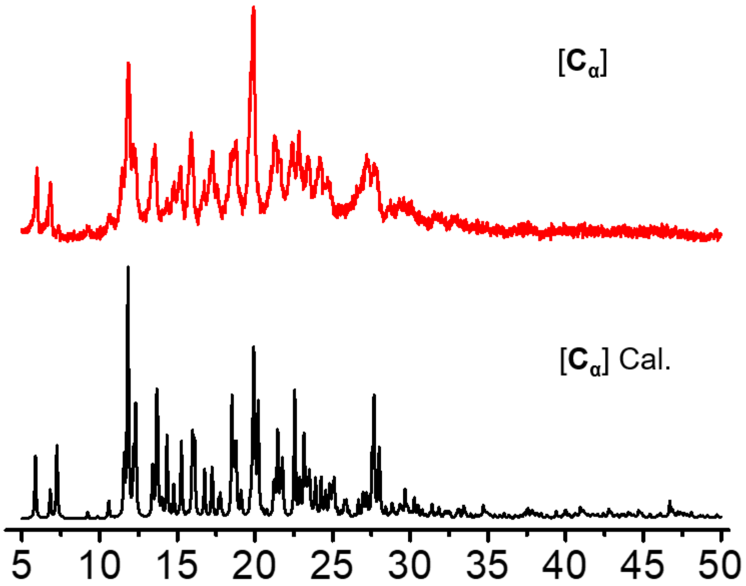

Supplementary Figure 9. Experimental (top) and simulated (bottom) PXRD patterns of $\mathbf{C_\alpha}$.

In the single crystal structure of [$\boldsymbol{D_{4d}}$**-CDMB-8⊃**$(NB)_2$•2NB] ($\mathbf{C_\beta}$), two nitrobenzene (NB) guests are located in the centre between two macrocycles via possible CH-π and π-π donor-acceptor interactions (Supplementary Fig. 10). Two $\boldsymbol{D_{4d}}$**-CDMB-8** and eight nitrobenzene molecules form a repeat unit that presumably favours further 1D packing (Supplementary Fig. 11).

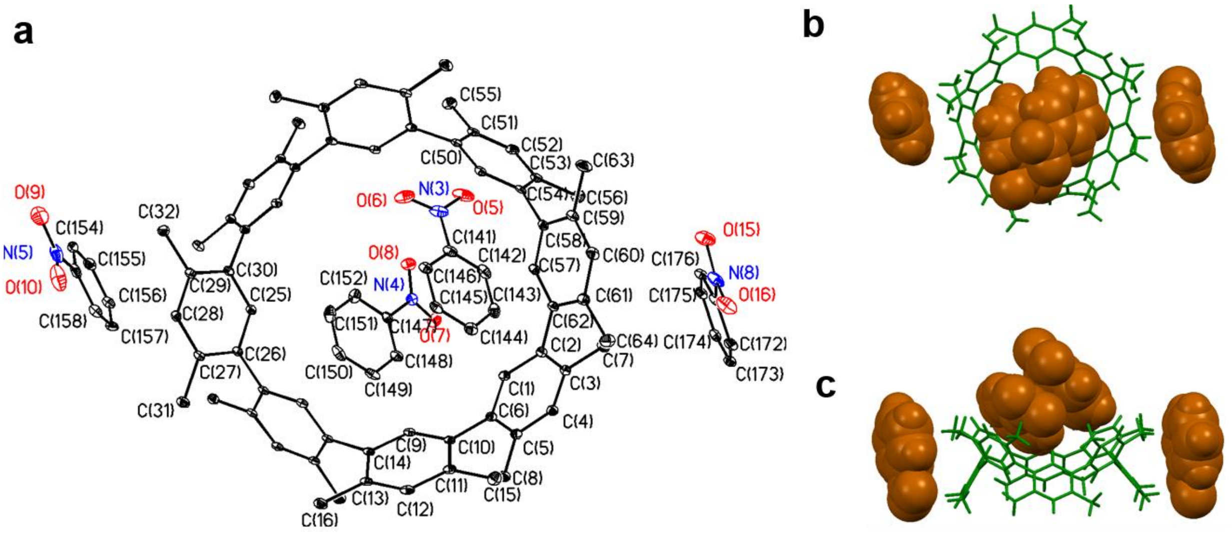

Supplementary Figure 10. **a**, Top view in ellipsoid form showing the interactions between $\boldsymbol{D_{4d}}$**-CDMB-8** and nitrobenzene with N(3), N(4), and N(8) observed in the single crystal structure of [$\boldsymbol{D_{4d}}$**-CDMB-8⊃**$(NB)_2$•2NB] ($\mathbf{C_\beta}$). **b** and **c**, Structure is shown as top and side

views in stick form. Displacement ellipsoids are scaled to the 25% probability level. All the other molecules and atoms have been omitted for clarity. Possible CH-π interactions are inferred from the following selected distances [Å]: C(57)---C(144) 3.744(4), C(144)---C(5) 3.893(4), C(150)---C(21) 3.711(4), C(150)---C(22) 3.851(4). Possible π-π donor-acceptor interactions are inferred from the following selected interatomic distances [Å]: N(3)---C(51) 3.440(4), N(3)---C(52) 3.491(4), C(141)---C(53) 4.027(4), C(142)---C(53) 4.170(4), N(4)---C(142) 3.514(4), N(4)---C(141) 3.570(4), O(8)---C(141) 3.465(4), C(142)---C(147) 3.783(4), C(143)---C(148) 3.792(4), C(144)---C(149) 3.769(4), C(150)---C(145) 3.725(4), C(151)---C(146) 3.703(4), C(152)---C(146) 3.461(4), C(152)---C(141) 3.721(4), C(158)---C(28) 3.730(4), C(148)---C(144) 3.539(4), N(8)---C(60) 3.645(4).

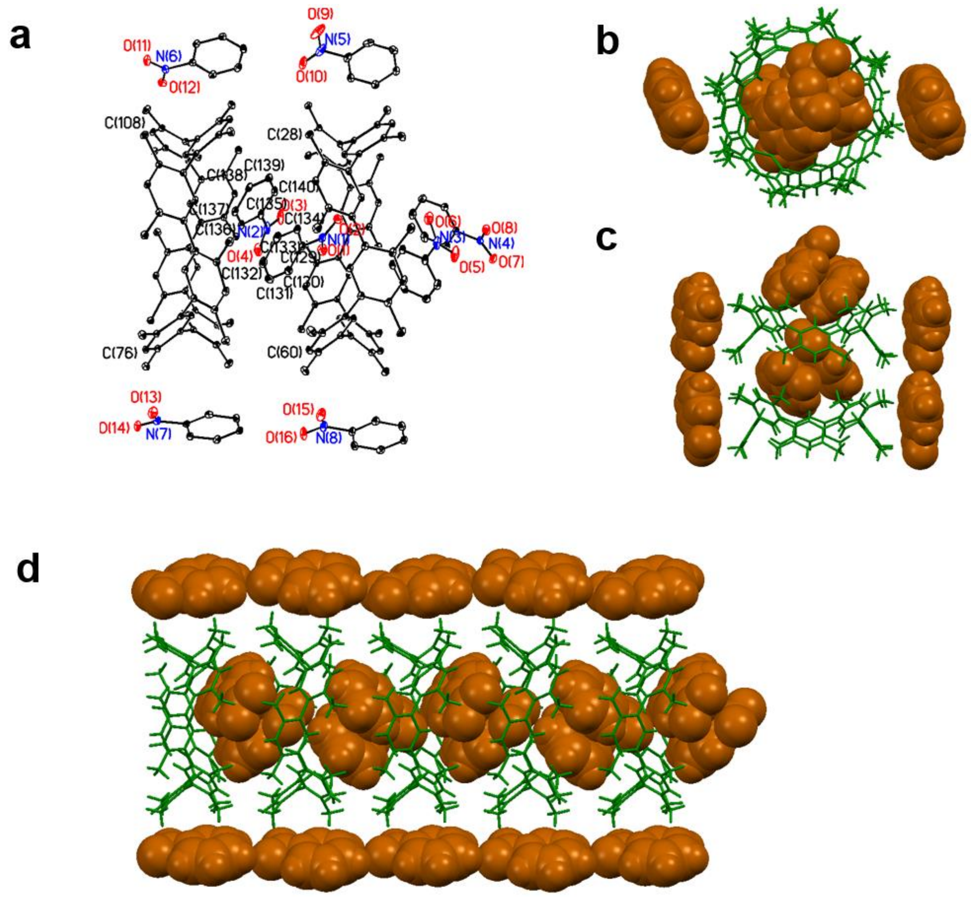

Supplementary Figure 11. **a**, Top view in ellipsoid form showing the dimeric ***D*4d-CDMB-8⊃**(NB)$_2$•2NB seen in the single crystal structure of [***D*4d-CDMB-8⊃**(NB)$_2$•2NB] (**C**$_{\beta}$). Displacement ellipsoids are scaled to the 25% probability level. **b**, top and **c**, side views of the dimer in stick form. **d**, 1D packing structure seen within single crystals of **C**$_{\beta}$.

In the single crystal structure of [(***D_{4d}*-CDMB-8**)$_2$⊃(**Py**)$_2$•3Tol] (**C_{δ}**), the head of the perylene guest is inserted within the macrocycle cavity and appears to be stabilized via CH-π interactions. One toluene solvent molecule is also located in the centre between two macrocycles with apparent stabilization being provided by both CH-π and π-π donor-acceptor interactions (Supplementary Fig. 12). Two ***D_{4d}*-CDMB-8**, two perylene, and three toluene molecules form a repeat unit that allows for further 1D packing (Supplementary Fig. 13).

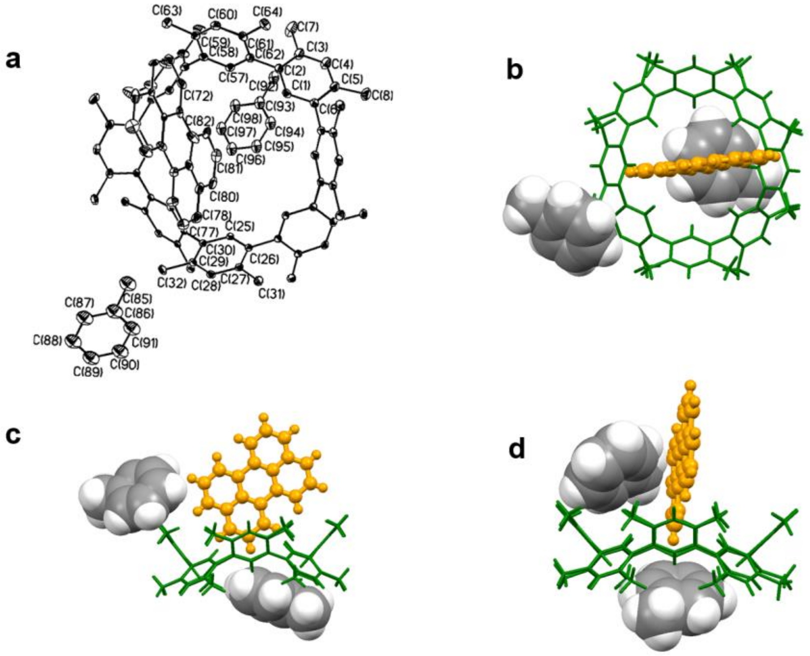

Supplementary Figure 12. **a**, Top view in ellipsoid form showing the binding interactions between ***D_{4d}*-CDMB-8**, **Py**, and a neighbouring toluene guest observed in the single crystal structure of [(***D_{4d}*-CDMB-8**)$_2$⊃(**Py**)$_2$•3Tol] (**C_{δ}**). **b**, Structure shown as a top view. **c** and **d**, Side views in stick form. Displacement ellipsoids are scaled to the 25% probability level. All other molecules and atoms have been omitted for clarity. Possible CH-π interactions are inferred from the following selected distances [Å]: C(81)---C(95) 3.872(5), C(81)---C(96) 3.957(4), C(78)---C(28) 3.644(3), C(80)---C(25) 3.759(1), C(73)---C(60) 3.651(2), C(72)---C(57) 4.337(9), C(31)---C(88) 3.43(5), C(31)---C(87) 3.55(9), C(92)---C(3) 3.63(6), C(57)---C(93) 3.650(0).

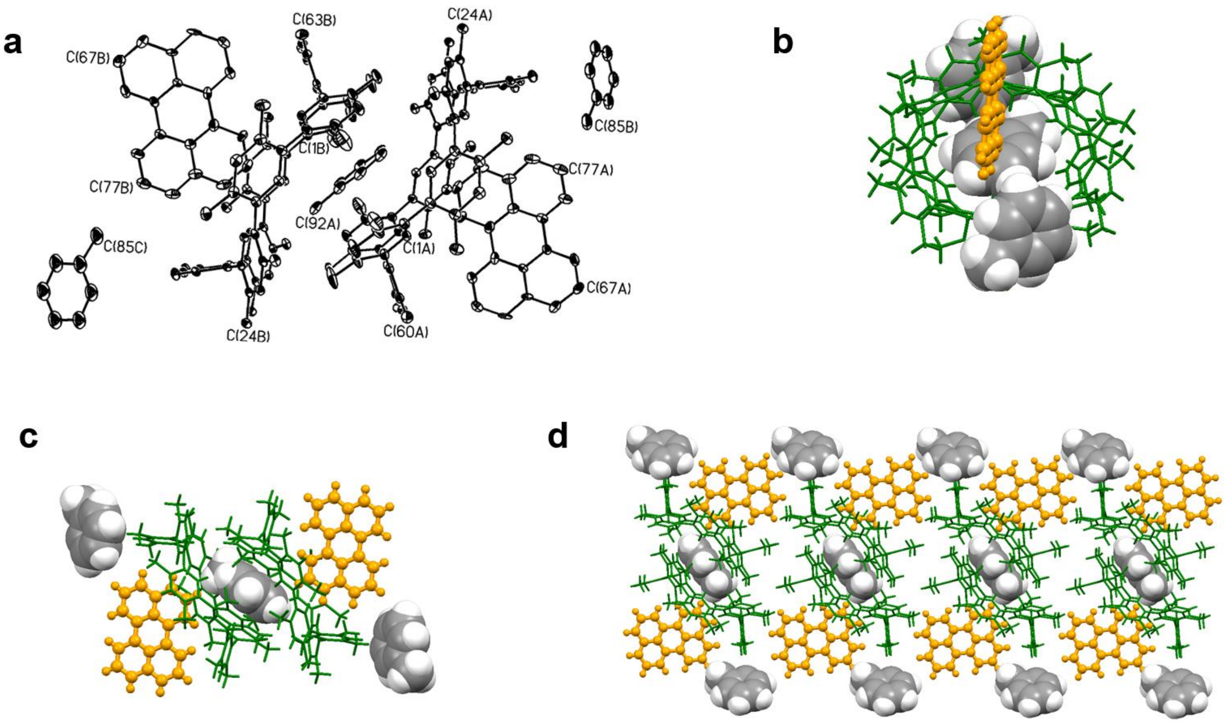

Supplementary Figure 13. **a**, Top view in ellipsoid form showing the dimer (**D_{4d}-CDMB-8**)$_2$⊃(**Py**)$_2$•3Tol seen in the single crystal structure of [(**D_{4d}-CDMB-8**)$_2$⊃(**Py**)$_2$•3Tol] (**$C_δ$**). Displacement ellipsoids are scaled to the 25% probability level. **b**, top and **c**, side views of the dimer in stick form. **d**, 1D packing structure seen within single crystals of **$C_δ$**.

Section S4: Organic solvent vapour promoted solid state molecular motion and related structural and emission response studies of materials formed between ***D*$_{4d}$-CDMB-8**, perylene, and organic solvents

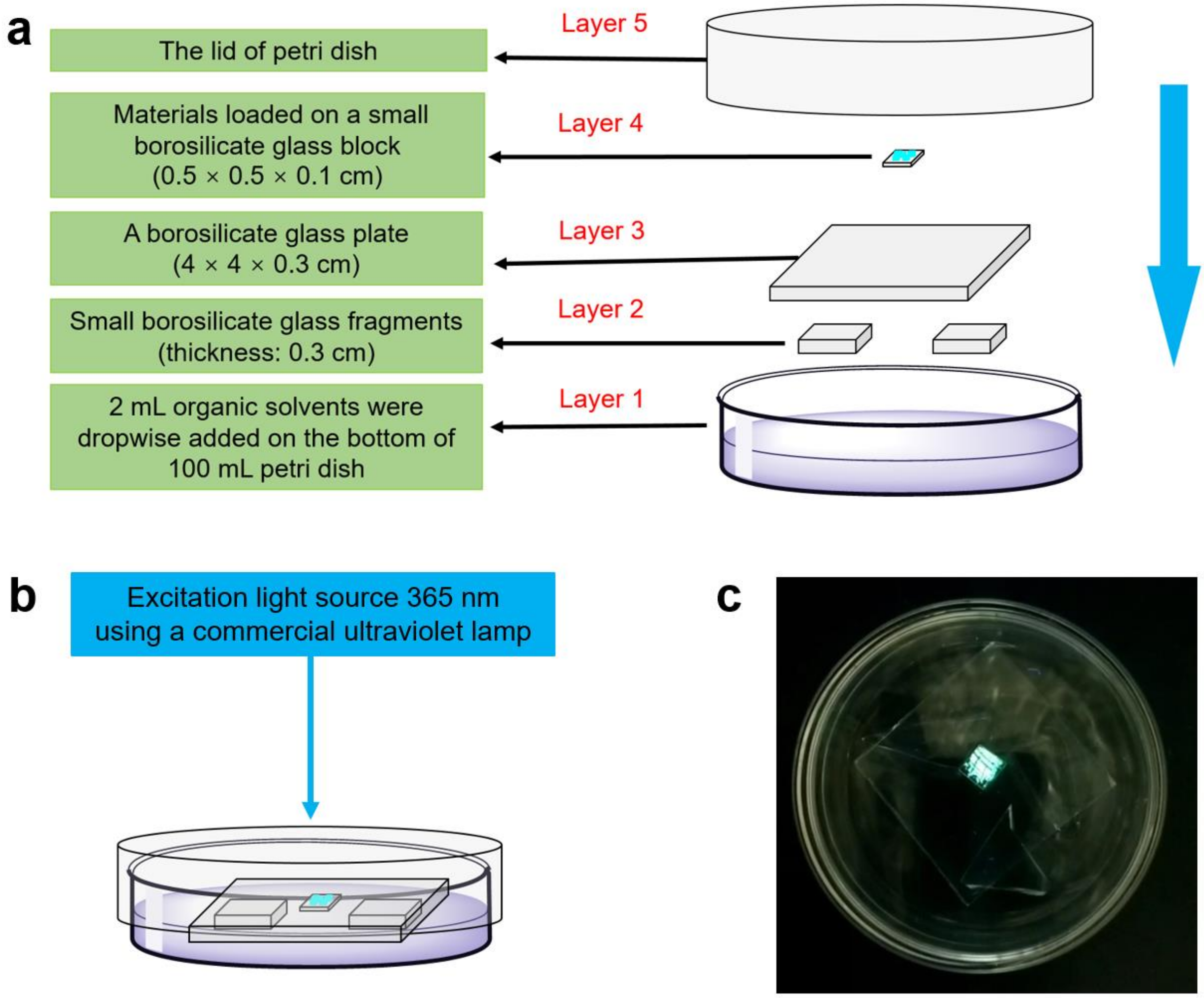

Supplementary Figure 14. **a**, **b**, Schematic representation and **c**, actual picture of organic solvent vapour treatment conditions used to access various materials formed from ***D*$_{4d}$-CDMB-8**, perylene, and organic solvents.

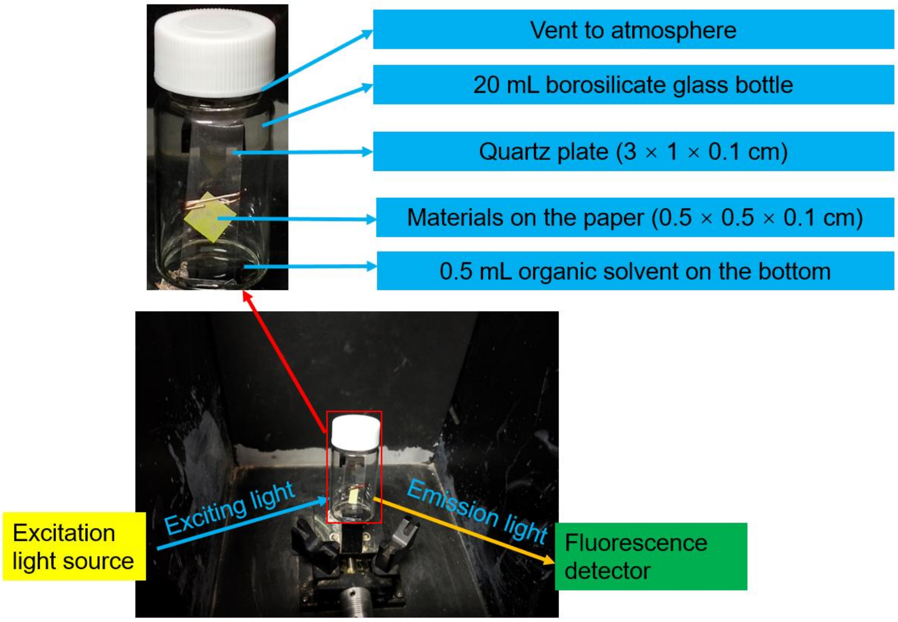

Supplementary Figure 15. In situ time-dependent emission spectra collection conditions for the materials formed from ***D*$_{4d}$-CDMB-8**, perylene, and various organic solvents (see main text for a listing) seen upon exposing to organic solvents in the form of their respective vapours.

As noted in the main text, when **C$_α$** was allowed to sit on the bench or in the air (298 K) for six days or subject to vacuum (2.0 kPa) for 5 hours conversion to **I$_α$** occurs. PXRD analyses revealed the crystalline structures of **C$_α$** and **I$_α$** are similar (Supplementary Figs. 16 and 17). The lower resolution seen for the PXRD spectrum of **I$_α$** may reflect the smaller particle size as well as time-dependent crystal weathering. Single crystal X-ray diffraction analyses of samples of **C$_α$** monitored under conditions used to produce **I$_α$** revealed that the sample retained essentially the same unit cell parameters when allowed sit for 24 h in the air or for in 1 hour in vacuum. However, longer treatment times under both conditions led to further crystal weathering. Moreover, a ^{1}H NMR spectral study of these samples revealed a reduction in the relative molar ratio of CH_3CN and THF as a function of time (Supplementary Figs. 18 and 19). Taken in concert, these findings lead us to suggest that the intermediates leading to **I$_α$** maintain a structural framework similar to **C$_α$** even as some solvent components are lost. Subjecting **C$_α$** or **I$_α$** to grinding led to an amorphous material (**A$_m$**) as established by a PXRD study (Supplementary Fig. 16b). ^{1}H NMR spectral analysis revealed that **A$_m$** only contains ***D*$_{4d}$-CDMB-8** and **Py** and is free of organic solvents (Supplementary Fig. 20). Treating **A$_m$** with THF/CH_3CN (1/1, *v*/*v*) vapour could be used to access **C$_α$** (Supplementary Fig. 16c).

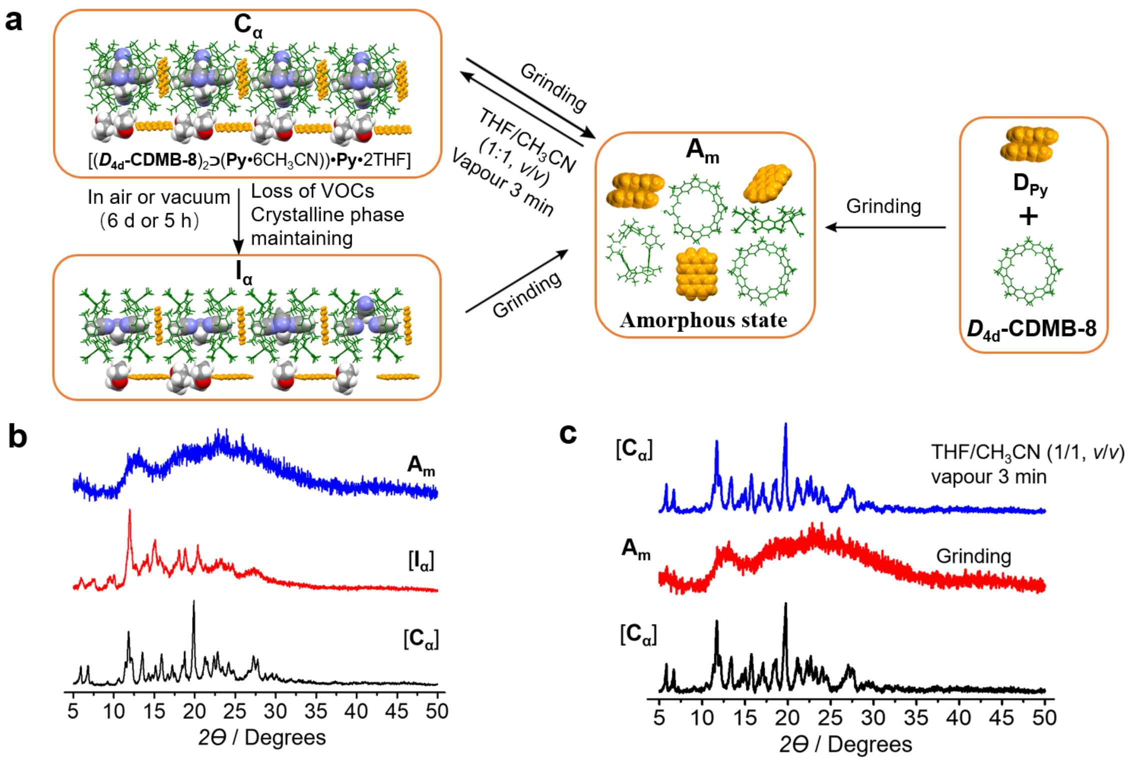

Supplementary Figure 16. **a**, Transformation between crystalline materials **C**$_{\alpha}$, **A**$_{m}$, and **I**$_{\alpha}$. **b**, Experimental PXRD patterns of **C**$_{\alpha}$, **I**$_{\alpha}$ and **A**$_{m}$. **c**, PXRD studies of the reversible transformation between co-crystalline **C**$_{\alpha}$ or **I**$_{\alpha}$ species and amorphous **A**$_{m}$.

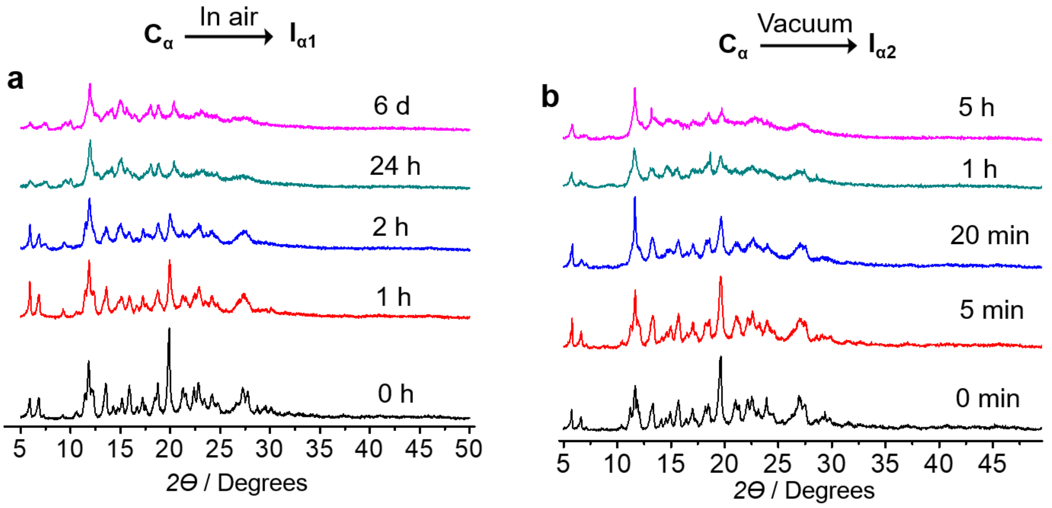

Supplementary Figure 17. Time-dependent experimental PXRD patterns corresponding to the conversion of **C**$_{\alpha}$ into **I**$_{\alpha1}$ seen upon letting stand in the air (**a**) or **I**$_{\alpha2}$ subjecting to vacuum (2.0 kPa) (**b**) at 298 K.

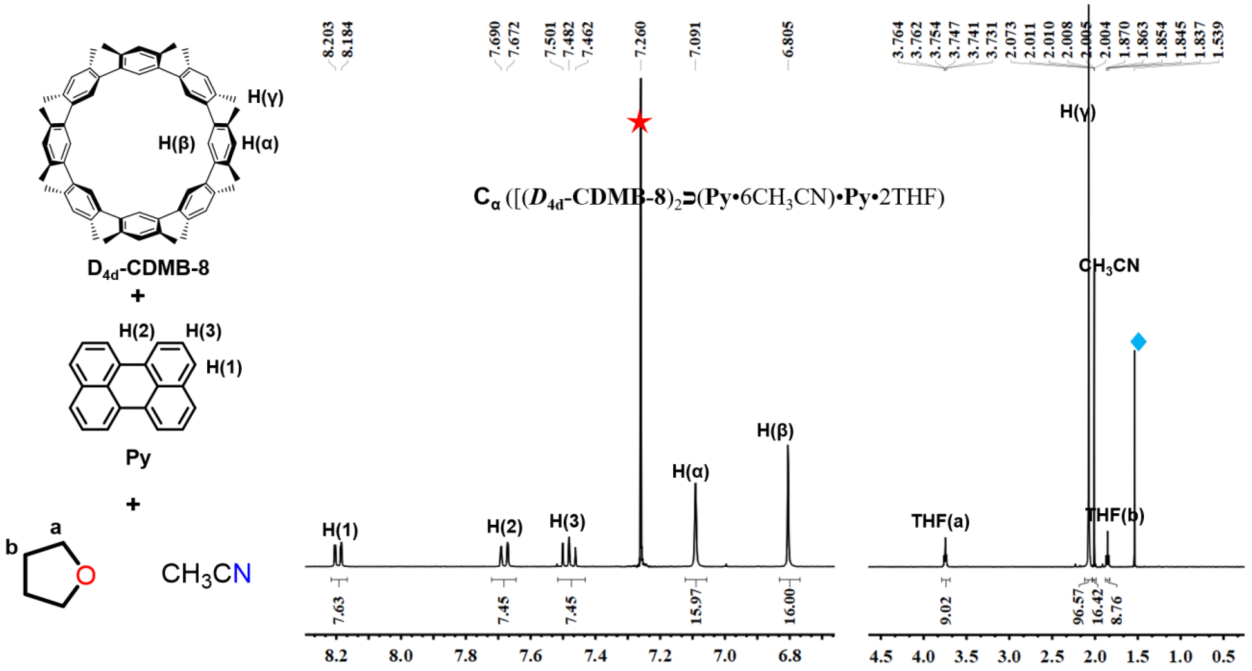

Supplementary Figure 18. ^{1}H NMR (400 MHz) spectrum of **C**$_{\alpha}$ recorded in $CDCl_3$ at 298 K (1 mg/ml) (red "★" represents residual $CHCl_3$, blue "♦" represents H_2O).

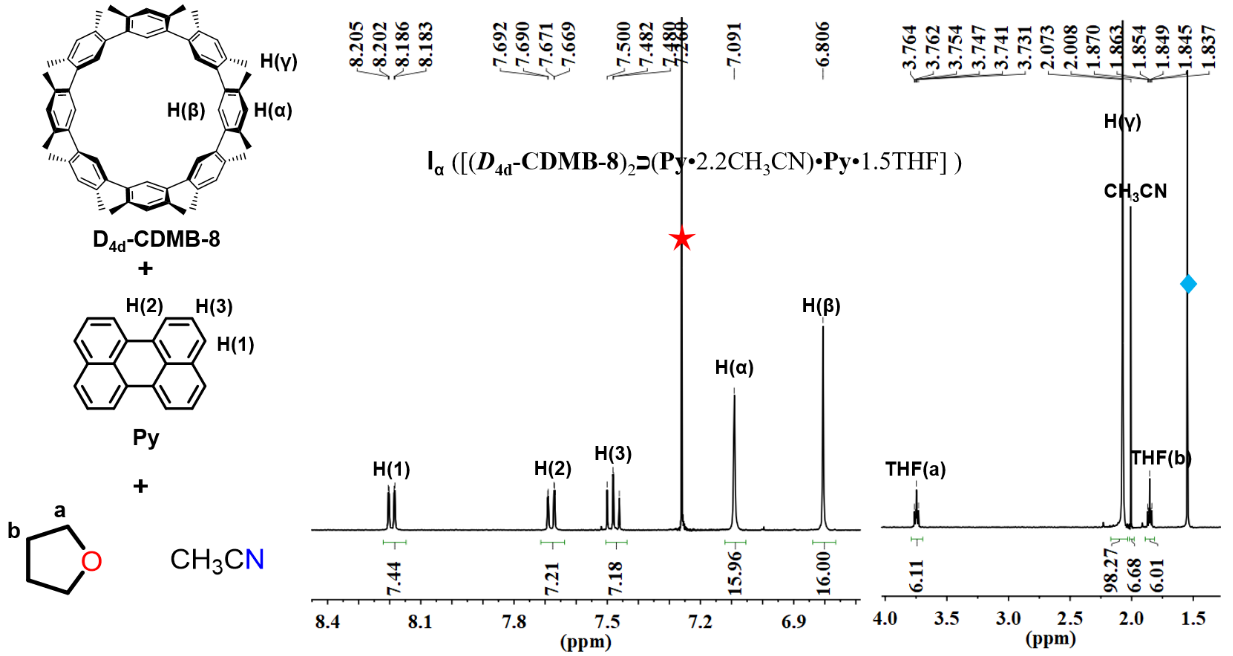

Supplementary Figure 19. ^{1}H NMR (400 MHz) spectrum of **I**$_{\alpha}$ recorded in $CDCl_3$ at 298 K (1 mg/ml) (red "★" represents residual $CHCl_3$, blue "♦" represents H_2O).

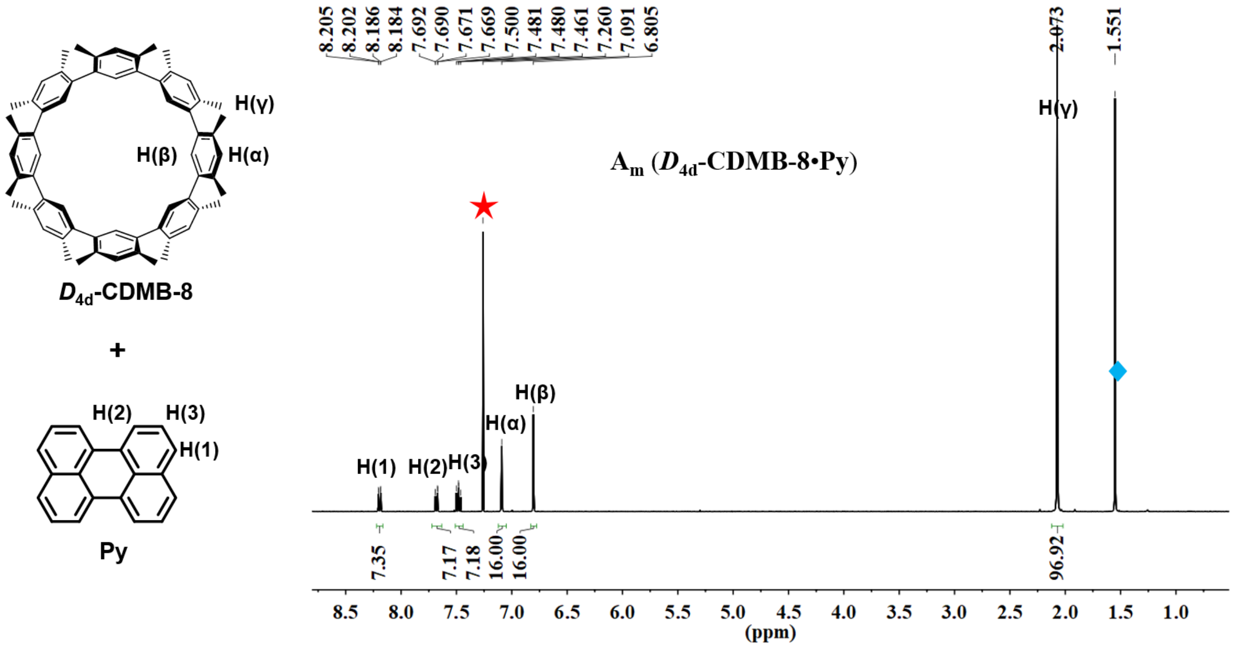

Supplementary Figure 20. ^{1}H NMR (400 MHz) spectrum of $\mathbf{A_m}$ recorded in $CDCl_3$ at 298 K (1 mg/ml) (red "★" represents residual $CHCl_3$, blue "♦" represents H_2O).

The emission spectrum of $\mathbf{C_\alpha}$ is similar to that produced by perylene in solution when studied at relatively high concentrations (e.g., 10 mM in THF/CH_3CN = 1/1, *v*/*v*) as shown in Supplementary Fig. 21.

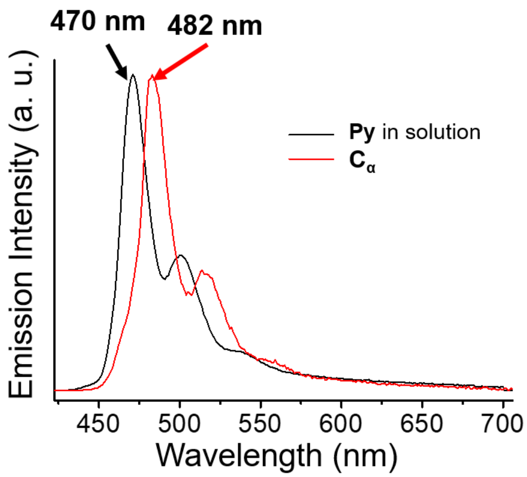

Supplementary Figure 21. Normalized emission spectra of $\mathbf{C_\alpha}$ (red curve) and **Py** (black curve, 2.00×10^{-2} M, THF/CH_3CN (1/1, *v*/*v*)) (λ_{ex} = 365 nm, voltage = 400 V, entrance slit width = 1 nm, exit slit width = 1 nm).

As $\mathbf{C_\alpha}$ is transformed to $\mathbf{I_\alpha}$ quenching of the emission feature at 482 nm is observed. The colour changes from green to yellow green. The amorphous material ($\mathbf{A_m}$) is characterized by a strong green yellow emission at 530 nm (Supplementary Fig. 22). Pictures were recorded under conditions of illumination using a commercially available ultraviolet lamp (365 nm)

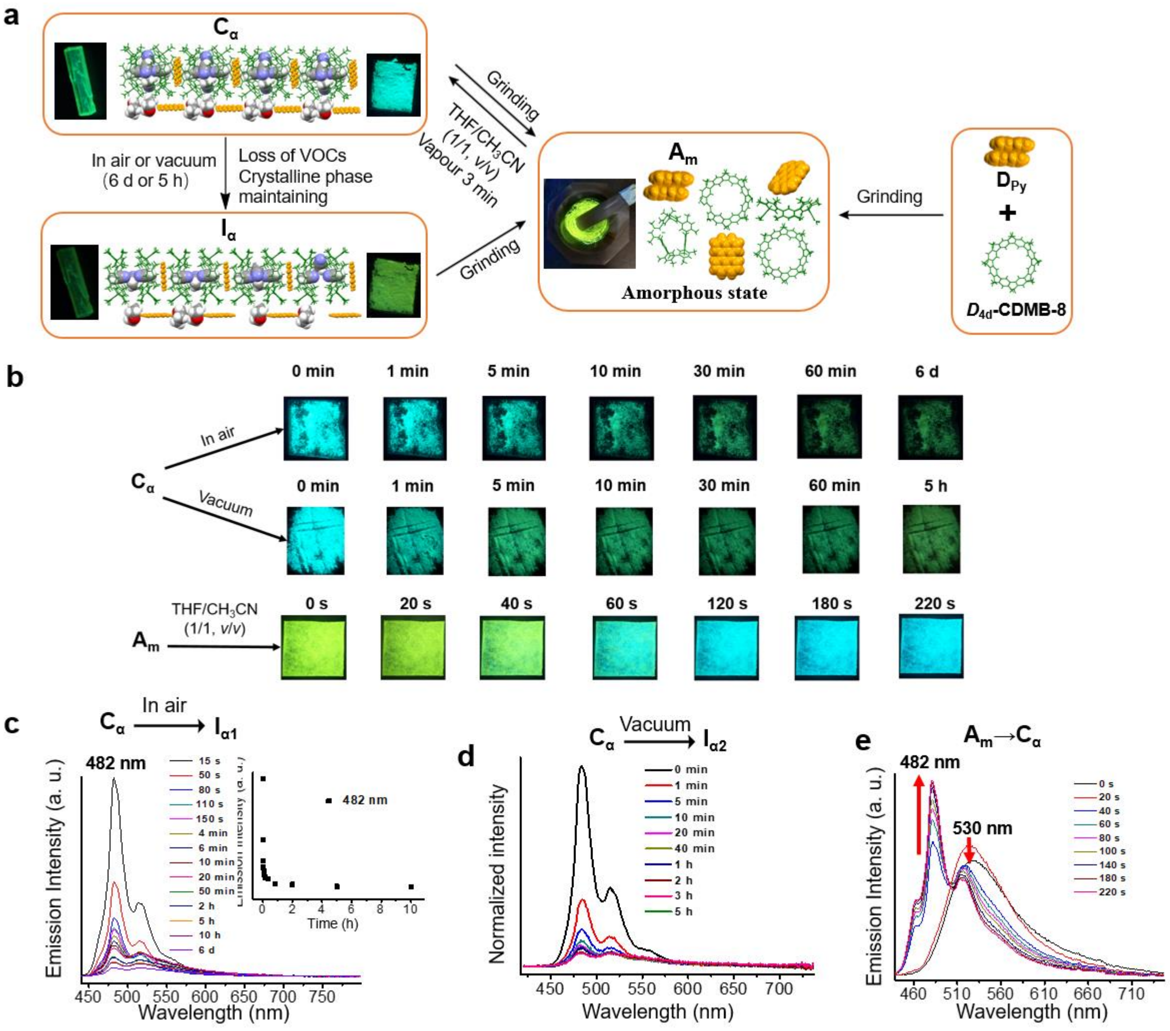

Supplementary Figure 22. **a**, Transformation between $\mathbf{C_\alpha}$, $\mathbf{A_m}$, and $\mathbf{I_\alpha}$. **b**, Photographs showing the colour changes seen for $\mathbf{C_\alpha}$ or $\mathbf{A_m}$ at different times when exposed to the air, vacuum (2.0 kPa), or THF/CH_3CN (1/1, *v*/*v*) vapour at 298 K with excitation provided by a commercial ultraviolet lamp (λ_{ex} = 365 nm). **c** and **d**, Time-dependent emission spectra corresponding to the conversion of $\mathbf{C_\alpha}$ into $\mathbf{I_\alpha}$ either in the air or under vacuum (2.0 kPa) at 298 K. **e**, Time-dependent emission spectra during the change process from $\mathbf{A_m}$ to $\mathbf{C_\alpha}$ in THF/CH_3CN (1/1, *v*/*v*) vapour (λ_{ex} = 365 nm, Voltage = 400 V, entrance slit width = 1 nm, exit slit width = 1 nm).

When $\mathbf{C}_\alpha$ was exposed to nitrobenzene (Nb) vapour, single crystal X-ray diffraction and PXRD analyses (Supplementary Fig. 23) revealed that $\mathbf{C}_\alpha$ maintains its structure over the course of 20 min. However, ^{1}H NMR spectral analysis revealed reduced levels of THF and CH_3CN and increasing (nearly 0.3 molar equiv.) levels of nitrobenzene in solution (Supplementary Fig. 26). These findings lead us to suggest that a new material ($\mathbf{I}_\beta$) is being produced as the result of nitrobenzene vapour covering the surface of $\mathbf{C}_\alpha$. Over longer time scales (from 20 min to 21 h), a crystalline transformation from $\mathbf{I}_\beta$ to mixed crystals ($\mathbf{M}_\alpha$) containing [***D*~4d~-CDMB-8**⊃(NB)$_2$•2NB] ($\mathbf{C}_\beta$) and dimeric perylene ($\mathbf{D}_{\mathbf{Py}}$)[2] is seen as inferred from ^{1}H NMR spectral measurements (Supplementary Fig. 27) combined with single crystal X-ray diffraction and PXRD analyses (Supplementary Figs. 23 and 24). $\mathbf{M}_\alpha$ could be transformed back to $\mathbf{I}_\beta$ and $\mathbf{C}_\alpha$ albeit with different dynamics (1 min vs. 24 h, respectively) via exposure to THF/CH_3CN (1/1, *v*/*v*) vapour (Supplementary Fig. 25).

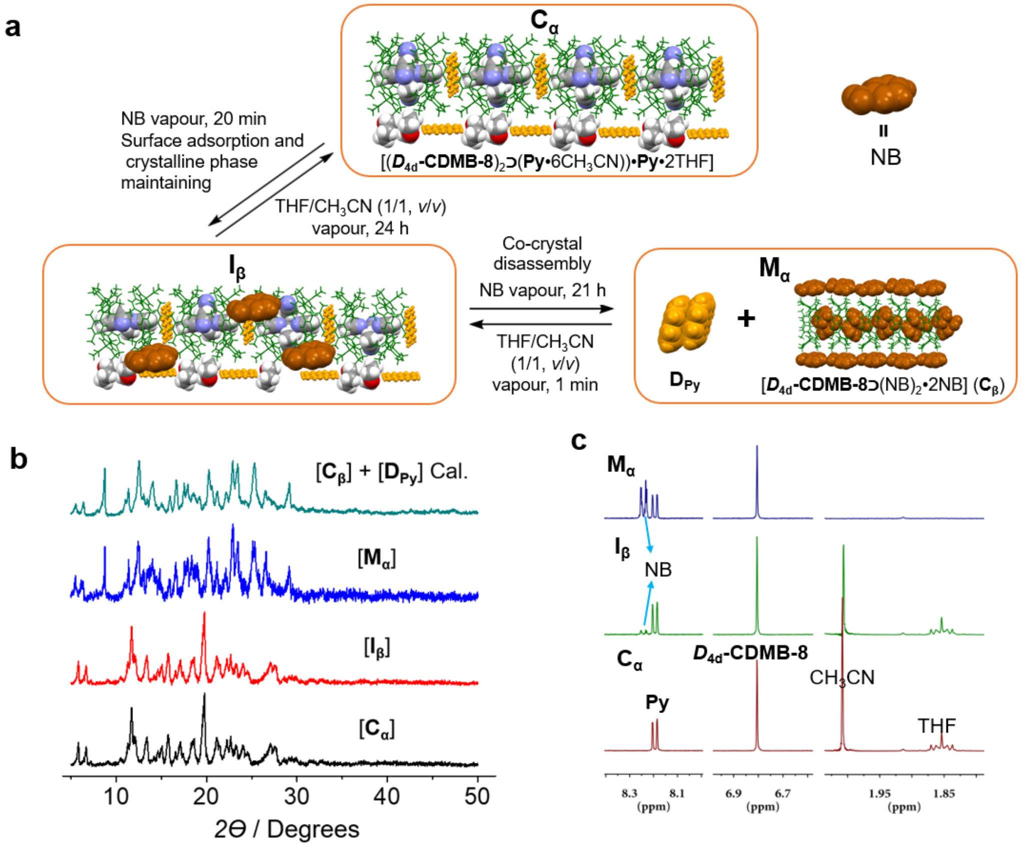

Supplementary Figure 23. a, Single crystal structures showing the transformation of co-crystalline $\mathbf{C}_\alpha$ to intermediate $\mathbf{I}_\beta$ and eventually the mixed crystal species $\mathbf{M}_\alpha$. **b**, Experimental PXRD patterns for $\mathbf{C}_\alpha$, $\mathbf{I}_\beta$, $\mathbf{M}_\alpha$, and simulated pattern of a mixture containing [***D*~4d~-CDMB-8⊃**

(NB)$_2$•2NB] and [$\mathbf{D_{Py}}$]. **c**, ^{1}H NMR spectral analyses (400 MHz) of the co-crystalline species $\mathbf{C_\alpha}$, $\mathbf{I_\beta}$, and mixed crystal form $\mathbf{M_\alpha}$ recorded in $CDCl_3$ at 298 K (1 mg/ml).

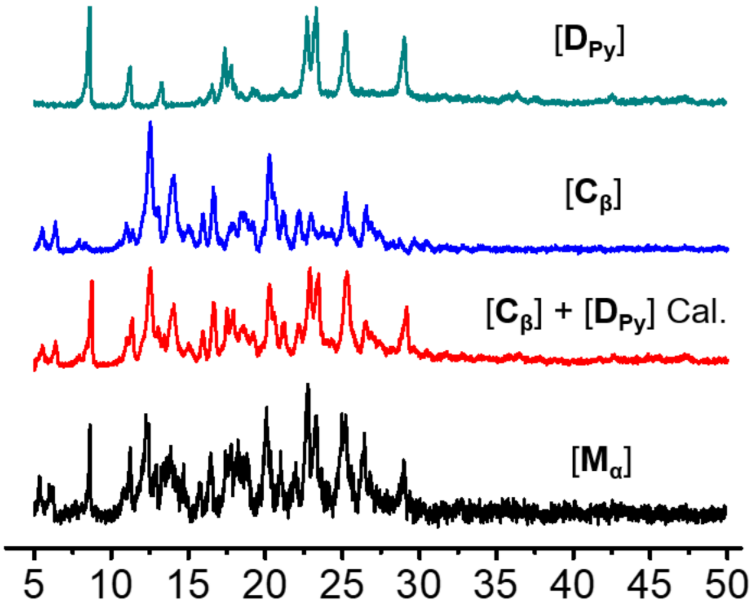

Supplementary Figure 24. Experimental PXRD patterns for $\mathbf{M_\alpha}$, $\mathbf{C_\beta}$, $\mathbf{D_{Py}}$, and simulated pattern of a mixture containing $\mathbf{C_\beta}$ and $\mathbf{D_{Py}}$.

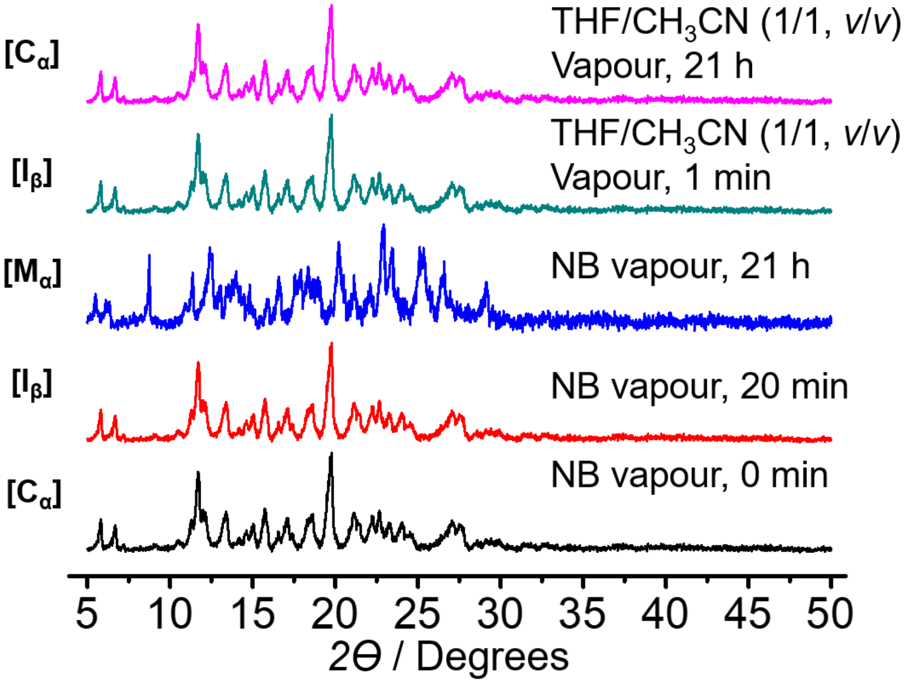

Supplementary Figure 25. PXRD studies of the reversible transformation between co-crystalline $\mathbf{C_\alpha}$, $\mathbf{I_\beta}$, and mixed crystal form $\mathbf{M_\alpha}$.

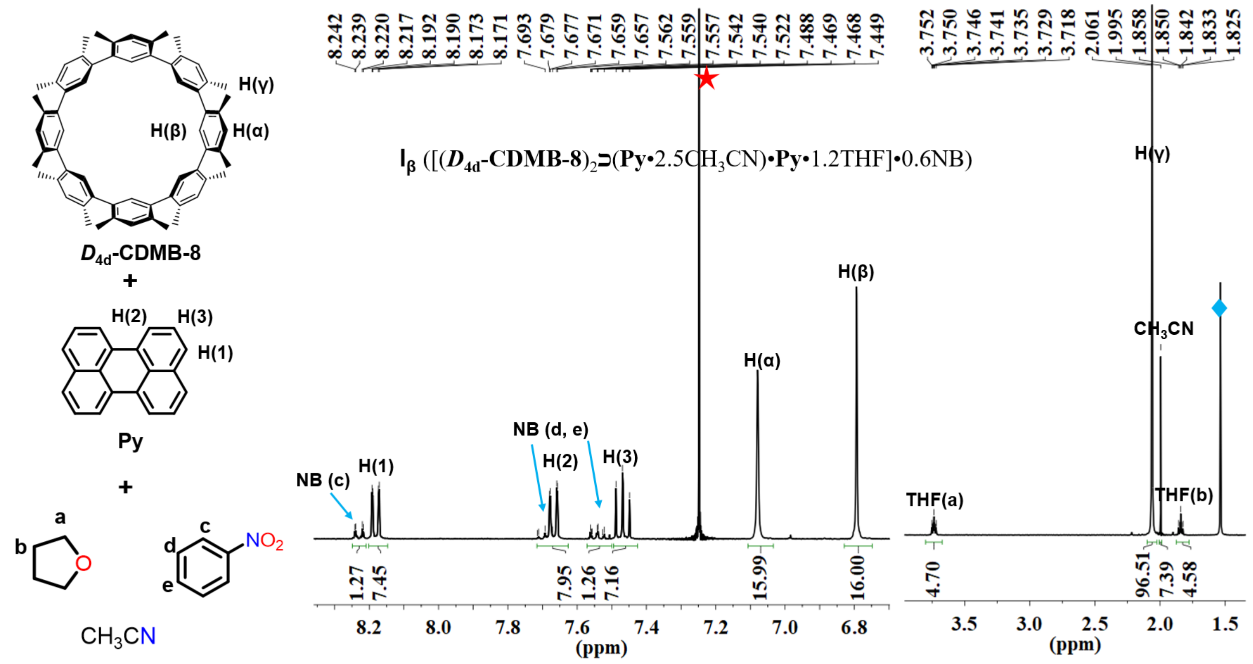

Supplementary Figure 26. ^{1}H NMR (400 MHz) spectrum of $\mathbf{I_\beta}$ recorded in $CDCl_3$ at 298 K (1 mg/ml) (red "★" represents residual $CHCl_3$, blue "♦" represents H_2O).

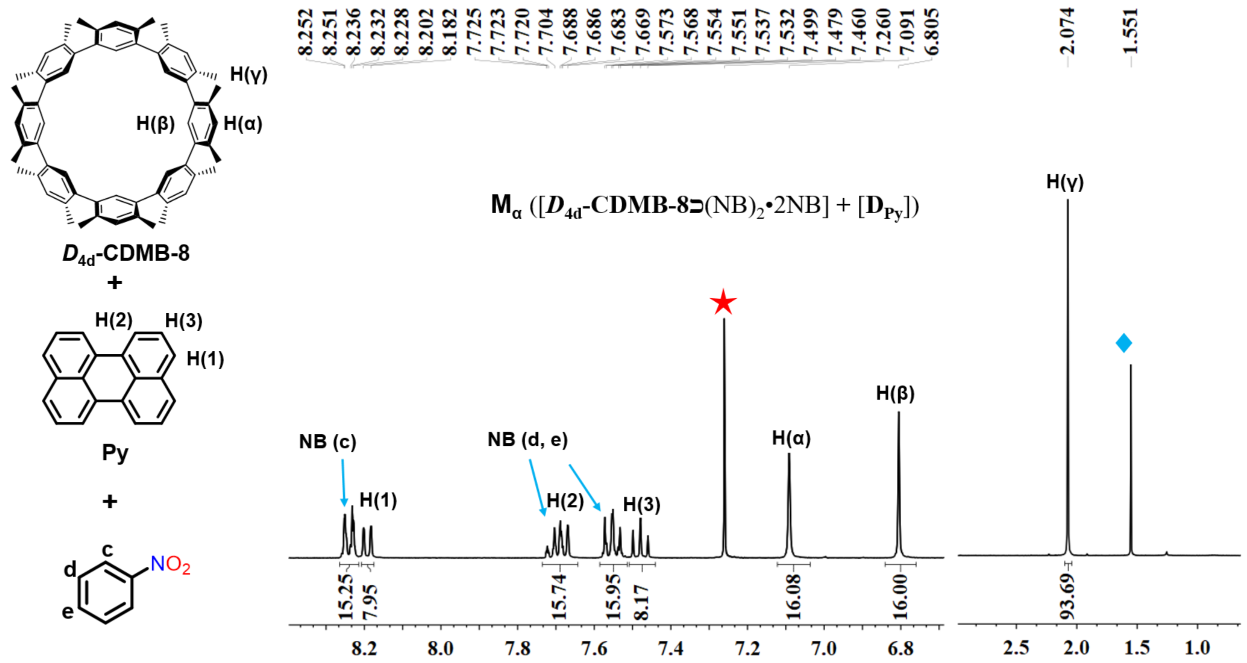

Supplementary Figure 27. ^{1}H NMR (400 MHz) spectrum of $\mathbf{M_\alpha}$ recorded in $CDCl_3$ at 298 K (1 mg/ml) (red "★" represents residual $CHCl_3$, blue "♦" represents H_2O).

When **C$_\alpha$** was transferred to **I$_\beta$** via nitrobenzene vapour treatment, the emission of **C$_\alpha$** at 482 nm was found to be almost completely quenched within 20 min. Over longer time scales (from 20 min to 21 h), a crystalline transformation from **I$_\beta$** to mixed crystalline material **M$_\alpha$** is seen that is accompanied by a rise in the emission intensity that is ascribed to the emission of **D$_{Py}$** (i.e., λ_{em} = 590 nm) (Supplementary Figs. 28-30). Upon exposure of **M$_\alpha$** to THF/CH_3CN (1/1, *v*/*v*) vapour, the intensity of the orange emission of **M$_\alpha$** at 590 nm was seen to undergo a reduction. However, an enhancement of the intensity at 482 nm, corresponding to the transformation of **M$_\alpha$** to **I$_\beta$**, and then to **C$_\alpha$** was seen over the course of 1 min and 24 h, respectively (Supplementary Fig. 30).

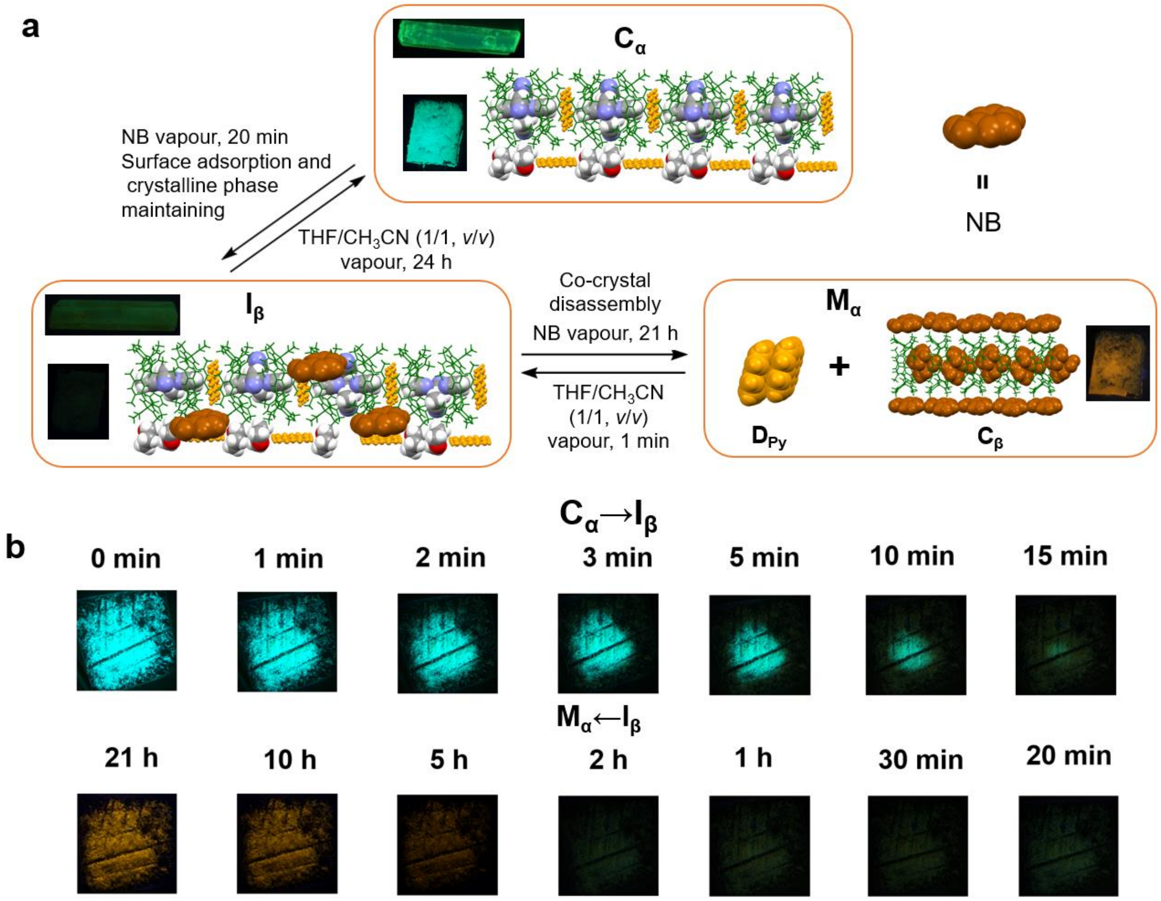

Supplementary Figure 28. **a**, Single crystal structures and photographs taken under illumination with an ultraviolet lamp purchased commercially (λ_{ex} = 365 nm) showing the transformation from co-crystalline **C$_\alpha$** to quenched intermediate **I$_\beta$**, and the mixed-crystalline species **M$_\alpha$**. **b**, Photographs of the nitrobenzene vapour-induced fluorescent colour changes for **C$_\alpha$** as seen at different times using a commercial ultraviolet lamp (λ_{ex} = 365 nm).

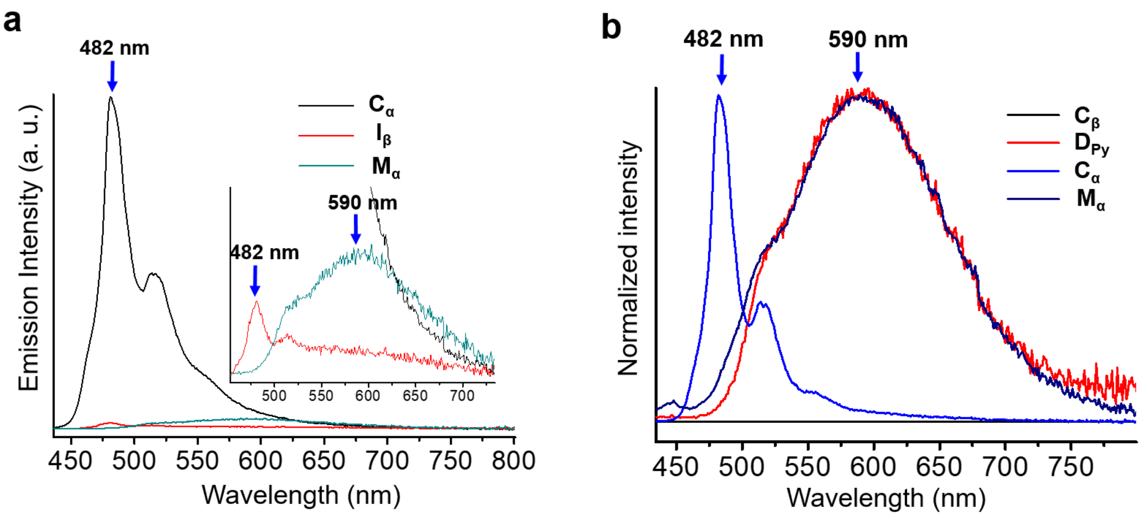

Supplementary Figure 29. **a**, Emission spectra of **C$_\alpha$**, **I$_\beta$**, and **M$_\alpha$**. **b**, Normalized emission spectra of **C$_\alpha$**, **M$_\alpha$**, **D$_{Py}$**, and **C$_\beta$** (λ_{ex} = 365 nm, voltage = 400 V, entrance slit width = 1 nm, exit slit width = 1 nm).

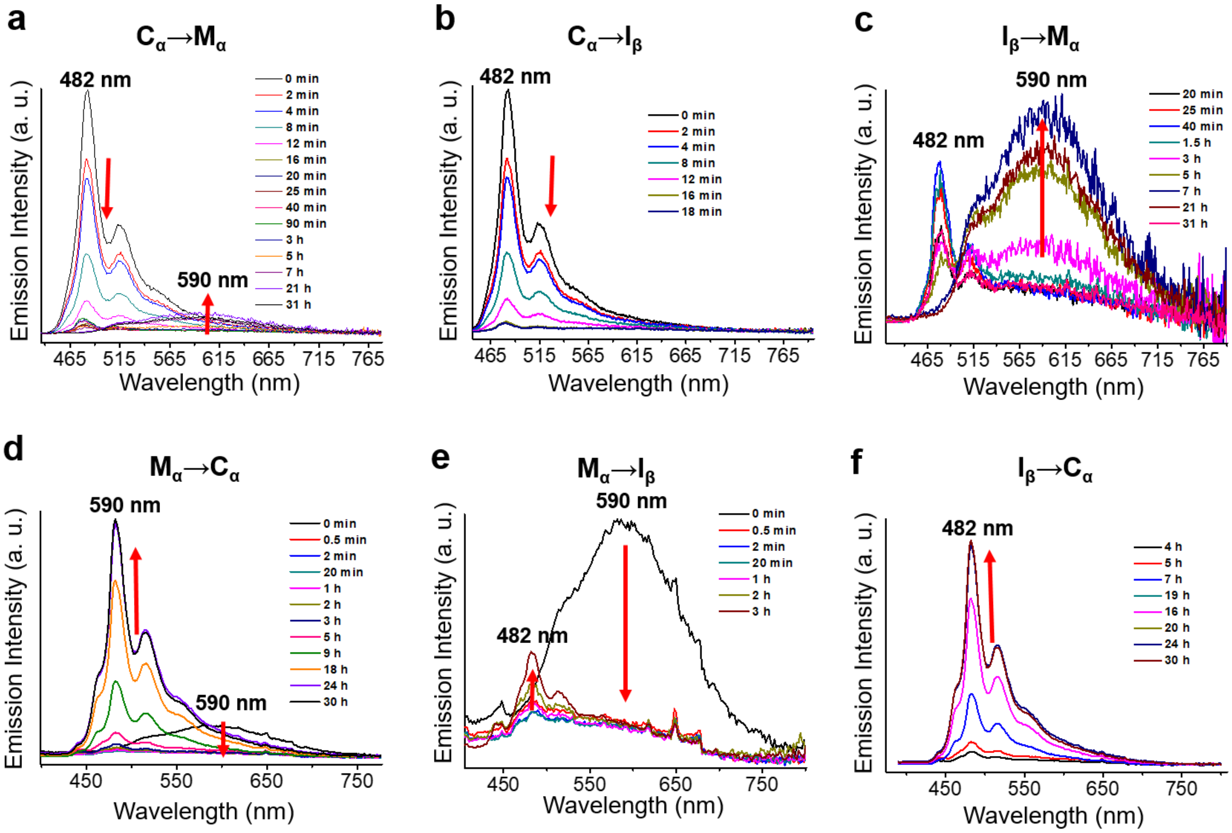

Supplementary Figure 30. **a-c**, Time-dependent emission spectra of **C$_\alpha$** to **I$_\beta$** and further to **M$_\alpha$** in nitrobenzene vapour. **d-f**, time-dependent emission spectra of **M$_\alpha$** to **I$_\beta$** and further to **C$_\alpha$** in

THF/CH_3CN (1/1, *v*/*v*) vapour (λ_{ex} = 365 nm, voltage = 400 V, entrance slit width = 1 nm, exit slit width = 1 nm).

In analogy to what was seen with nitrobenzene vapour, it was found that the co-crystalline material **C_α**, when exposed to THF vapour for 3 minutes, also generated a new mixed crystalline species, **M_β**, which would revert back to **C_α** via treatment with THF/CH_3CN (1/1, *v*/*v*) vapour for 30 minutes. X-ray diffraction details and ^{1}H NMR spectral analyses are summarised in Supplementary Figs. 31-33. Form **M_β** was found to contain single crystals of [***D*$_{4d}$-CDMB-8**⊃THF•THF][1] (**C_γ**) and **D_{Py}** as judged from X-ray diffraction analyses.

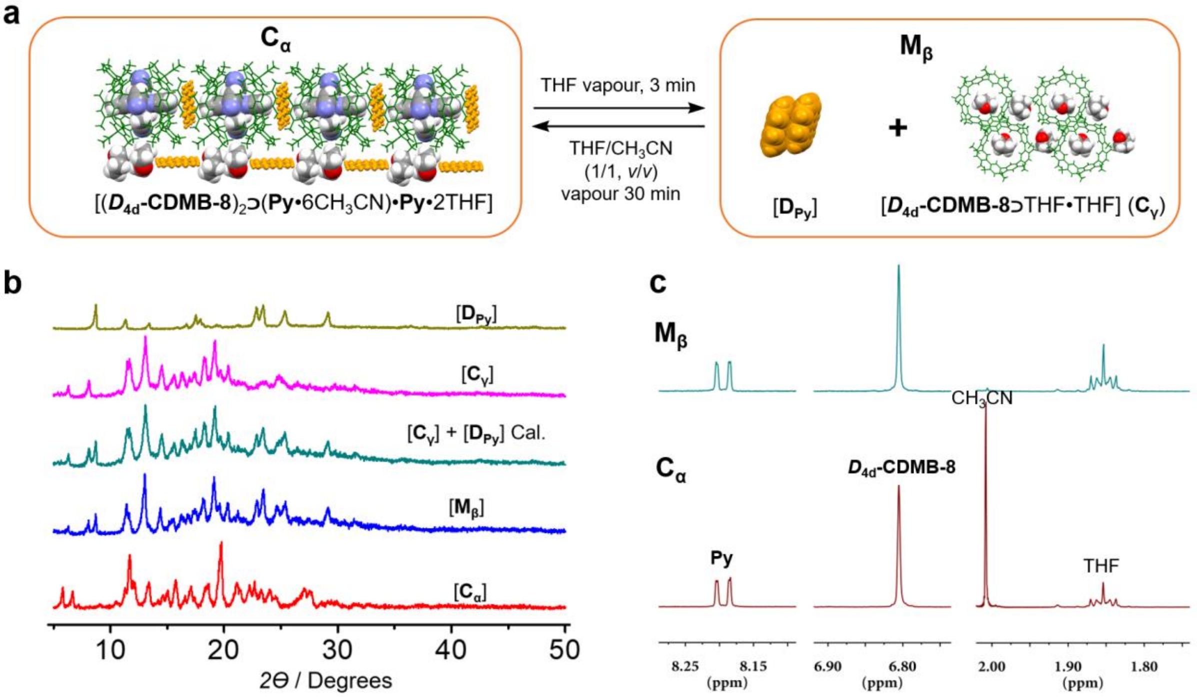

Supplementary Figure 31. **a**, Single crystal structures showing the transformation between the co-crystalline material **C_α** and a mixed crystalline species **M_β**. **b**, Experimental PXRD patterns of **C_α**, **M_β**, **C_γ**, **D_{Py}**, and the simulated PXRD pattern of the mixture containing **C_γ** and **D_{Py}**. **c**, ^{1}H NMR (400 MHz) spectra of **C_α** and **M_β** recorded in $CDCl_3$ at 298 K (1 mg/mL).

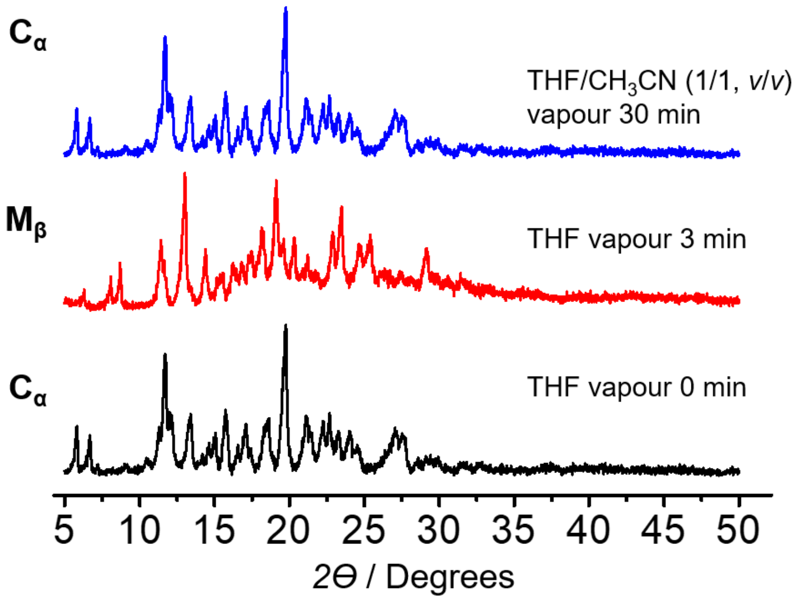

Supplementary Figure 32. PXRD studies of the reversible transformation between co-crystalline **C**$_{\alpha}$ and mixed crystalline **M**$_{\beta}$.

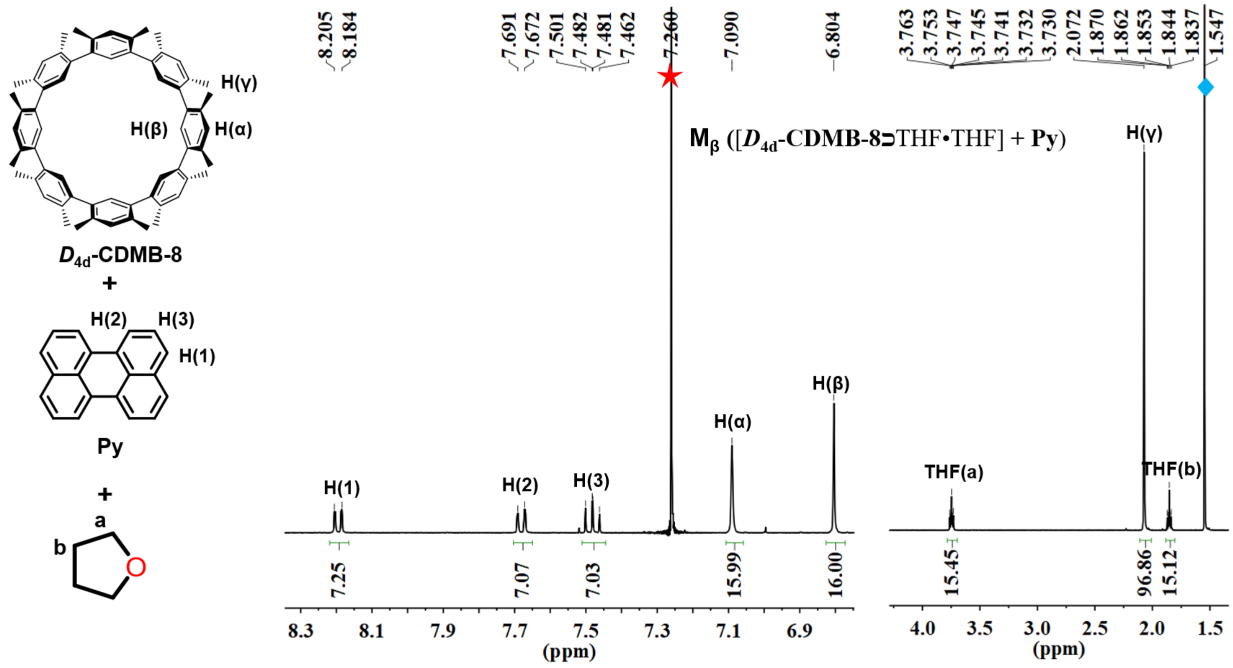

Supplementary Figure 33. ^{1}H NMR (400 MHz) spectrum of **M**$_{\beta}$ recorded in $CDCl_3$ at 298 K (1 mg/ml) (red "★" represents residual $CHCl_3$, blue "♦" represents H_2O).

The emission colour and the corresponding time-dependent change seen for **C**$_{\alpha}$ upon exposure to THF vapour are shown in Supplementary Fig. 34. Note the colour change from blue to orange.

A movie record of the transformation of $\mathbf{C_\alpha}$ into $\mathbf{M_\beta}$ in the presence of THF vapour is provided in Supplementary Movie 2. Pictures and movies are recorded under a commercial ultraviolet lamp (365 nm). The orange emission colour was found to revert back to the original blue after exposing $\mathbf{M_\beta}$ to THF/CH_3CN (1/1, *v*/*v*) vapour for 30 minutes (Supplementary Fig. 35).

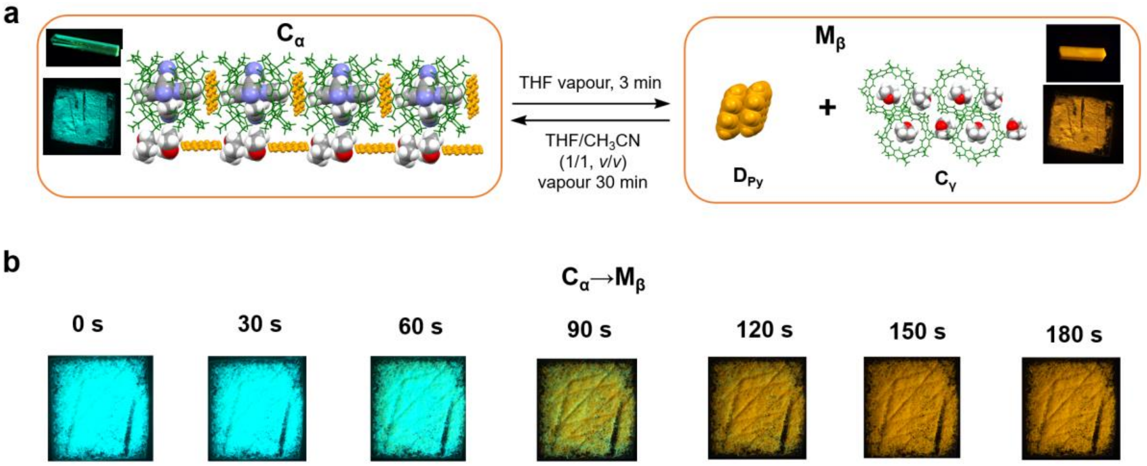

Supplementary Figure 34. **a**, Single crystal structures and photographs under a commercial ultraviolet lamp (λ_{ex} = 365 nm) showing the transformation of the co-crystalline material $\mathbf{C_\alpha}$ with fluorescence blue colour to a mixed crystalline species $\mathbf{M_\beta}$ with a characteristic fluorescence orange colour. **b**, Photographs of the THF vapour-induced fluorescent colour changes for $\mathbf{C_\alpha}$ as seen at different time scales with visualization provided by a commercial ultraviolet lamp (λ_{ex} = 365 nm).

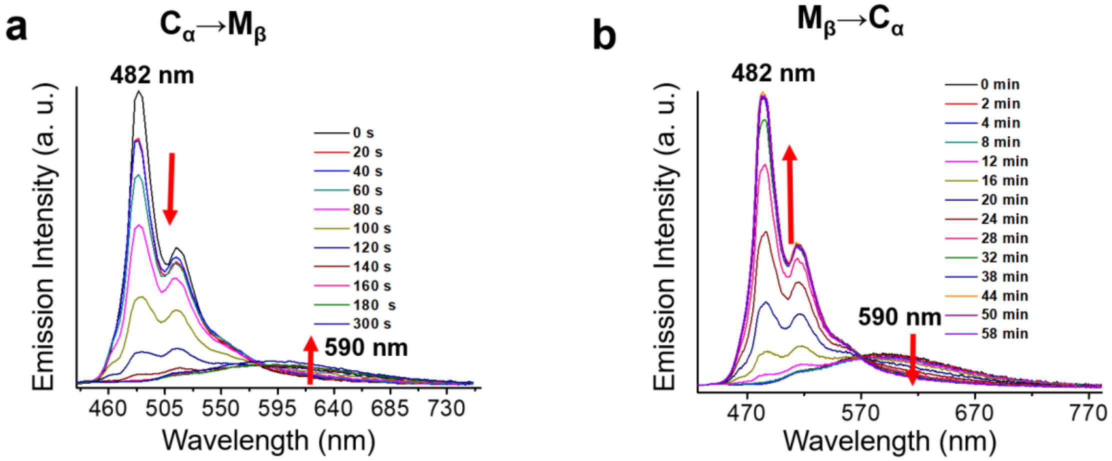

Supplementary Figure 35. **a**, Time-dependent emission spectra corresponding to the conversion of $\mathbf{C_\alpha}$ to $\mathbf{M_\beta}$ in THF vapour. **b**, Time-dependent emission spectra corresponding to the conversion of $\mathbf{M_\beta}$ to $\mathbf{C_\alpha}$ in THF/CH_3CN (1/1, *v*/*v*) vapour (λ_{ex} = 365 nm, voltage = 400 V, entrance slit width = 1 nm, exit slit width = 1 nm).

When **C$_\alpha$** was exposed in toluene vapour, a PXRD pattern for the toluene-treated material (**C$_\delta$**) is obtained that is completely different from that of the original **C$_\alpha$**. This finding leads support to the contention that a new complex is being created. The simulated PXRD pattern of **C$_\delta$** is in good accord with the single crystal data for [(***D*$_{4d}$-CDMB-8**)2⊃(**Py**)$_2$•3Tol] (Supplementary Fig. 36b). ^{1}H NMR spectral studies revealed that the components of ***C$_\delta$*** are the same as those of [(***D*$_{4d}$-CDMB**-8)$_2$⊃(**Py**)$_2$•3Tol] (Supplementary Fig. 38). Thus, in aggregate, these results serve to confirm that exposure to toluene vapour serves to transform the co-crystalline species **C$_\alpha$** into a different co-crystal, namely [(***D*$_{4d}$-CDMB-8**)$_2$⊃(**Py**)$_2$•3Tol] (**C$_\delta$**), and can act to regenerate to **C$_\alpha$** via treatment with THF/CH_3CN (1/1, *v*/*v*) vapour (Supplementary Fig. 37).

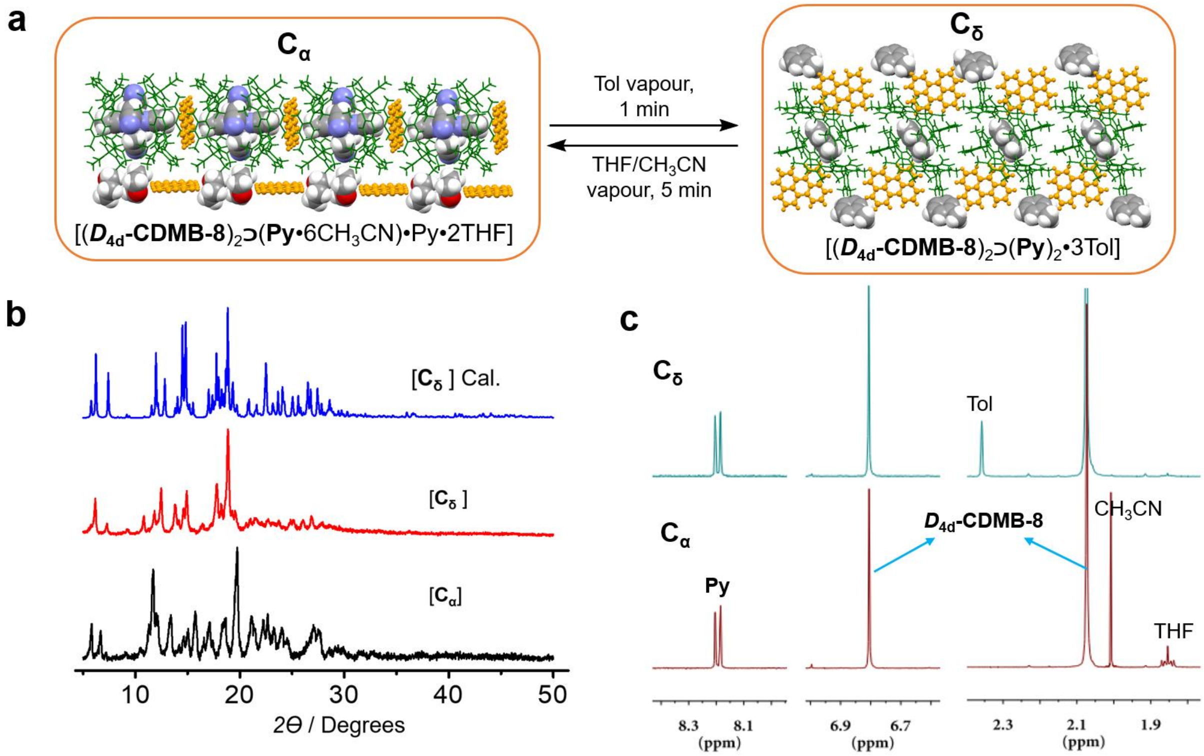

Supplementary Figure 36. **a**, Single crystal structures showing the transformation between co-crystalline materials **C$_\alpha$** and **C$_\delta$**. **b**, experimental PXRD patterns of **C$_\alpha$** and **C$_\delta$**, as well as the simulated PXRD pattern for [(***D*$_{4d}$-CDMB-8**)$_2$⊃(**Py**)$_2$•3Tol] (**C$_\delta$**). **c**, expanded ^{1}H NMR (400 MHz) spectra of **C$_\alpha$** and **C$_\delta$** recorded in $CDCl_3$ at 298 K (1 mg/ml).

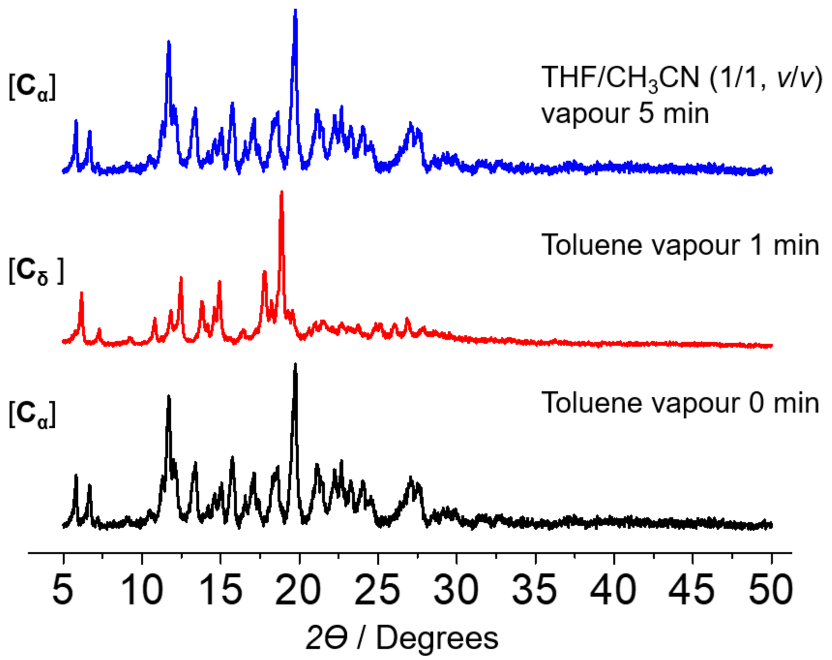

Supplementary Figure 37. PXRD studies of the reversible transformation between co-crystalline materials **C**$_{\alpha}$ and **C**$_{\delta}$.

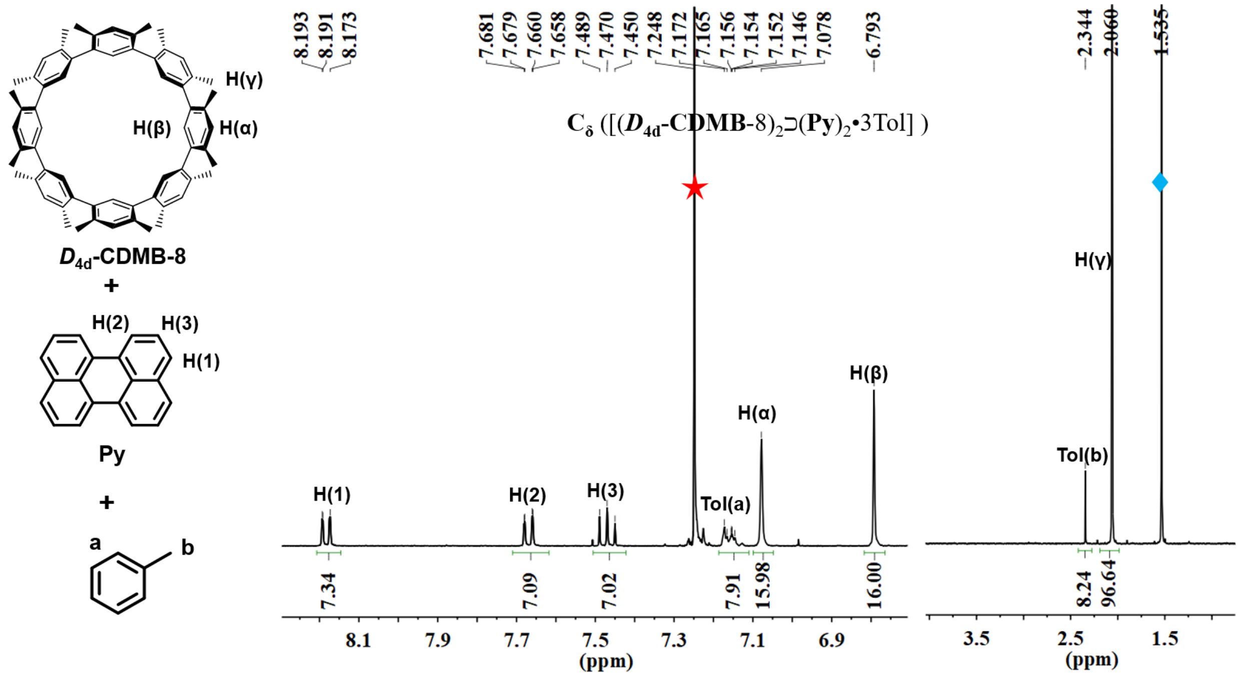

Supplementary Figure 38. ^{1}H NMR (400 MHz) spectrum of **C**$_{\delta}$ recorded in $CDCl_3$ at 298 K (1 mg/ml) (red "★" represents residual $CHCl_3$, blue "♦" represents H_2O) .

Only a small change in the emission features ($\lambda_{em,\ max}$ = 482 nm changing to $\lambda_{em,\ max}$ = 478 nm) was observed when **C$_\alpha$** was converted to **C$_\delta$** (Supplementary Fig. 39b). The emission spectrum of **C$_\delta$** proved concordant with that of [(***D*$_{4d}$-CDMB-8**)$_2$⊃(**Py**)$_2$•3Tol].

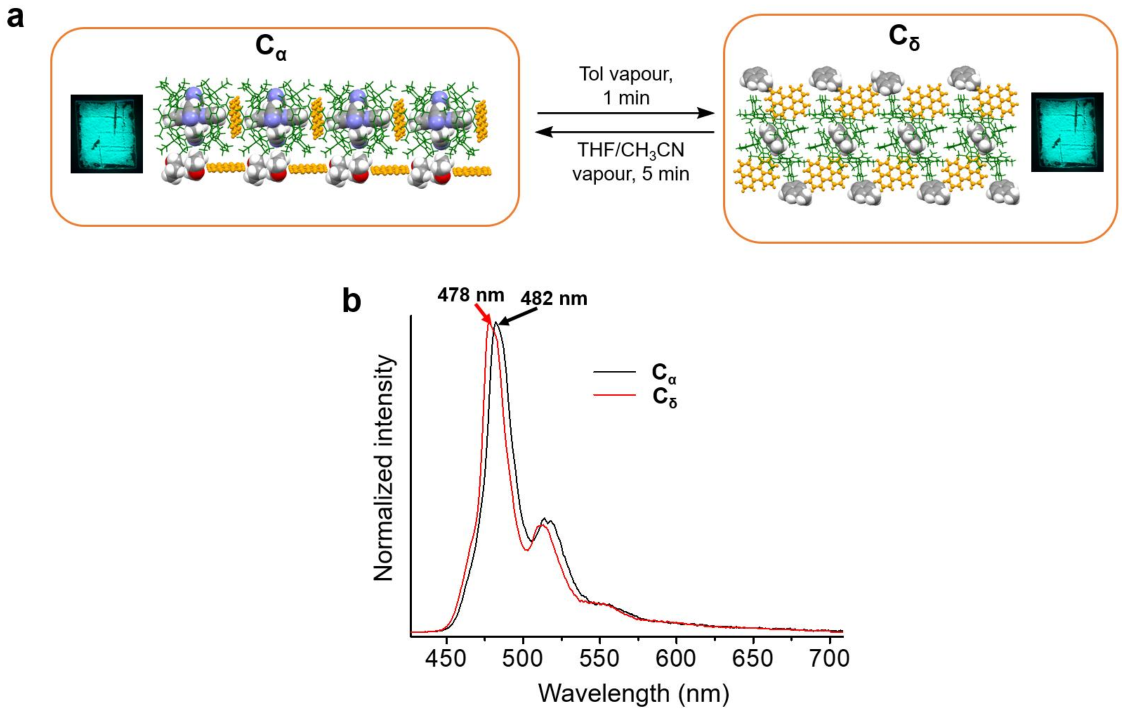

Supplementary Figure 39. **a**, Single crystal structures and photographs taken under a commercial ultraviolet lamp (λ_{ex} = 365 nm) showing the transformation between co-crystalline materials **C$_\alpha$** and **C$_\delta$**. **b**, Normalized emission spectra of **C$_\alpha$** and **C$_\delta$** (λ_{ex} = 365 nm, voltage = 400 V, entrance slit width = 1 nm, exit slit width = 1 nm).

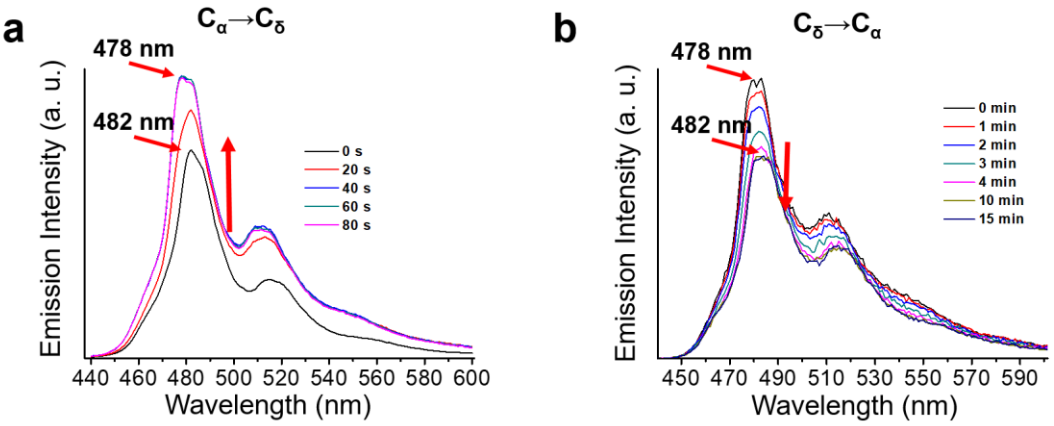

Supplementary Figure 40. **a**, Time-dependent emission spectra of **C$_\alpha$** to **C$_\delta$** in toluene vapour. **b**, Time-dependent emission spectra of **C$_\delta$** to **C$_\alpha$** in THF/CH_3CN (1/1, *v*/*v*) vapour (λ_{ex} = 365 nm, voltage = 400 V, entrance slit width = 1 nm, exit slit width = 1 nm).

Further studies demonstrating the reversible transformation between materials **C$_{\alpha}$**, **C$_{\delta}$**, **I$_{\beta}$**, **M$_{\alpha}$**, **M$_{\beta}$**, and **A$_{m}$** were carried out using solid fluorescent spectrometry (Supplementary Figs. 42-43) and summarised in Supplementary Fig. 41. As noted in the main text, treatment with different organic vapours or grinding can be used to induce the reversible transformation between these species. This can be followed by the fluorescence colour changes involved.

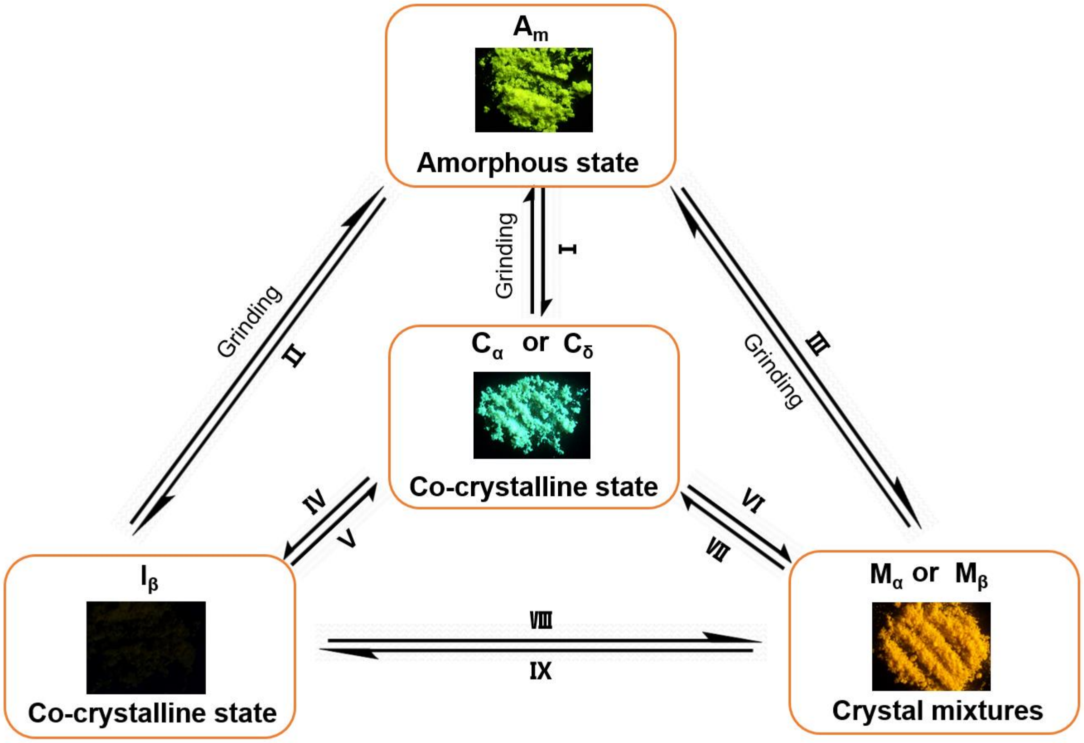

Supplementary Figure 41. Summary of the reversible transformations between **C$_{\alpha}$**, **C$_{\delta}$**, **I$_{\beta}$**, **M$_{\alpha}$**, **M$_{\beta}$**, and **A$_{m}$** via grinding or treatment with the vapour forms of various organic solvents. Vapour **I**: **A$_{m}$** to **C$_{\alpha}$**, THF/CH_3CN (1/1, *v*/*v*) (3 min), **A$_{m}$** to **C$_{\delta}$**, Tol (40 s). vapour **II**: **A$_{m}$** to **I$_{\beta}$**, NB/THF/CH_3CN (1/1/1, *v*/*v*/*v*) (1 min). vapour **III**: **A$_{m}$** to **M$_{\beta}$**, THF (1 min), **A$_{m}$** to **M$_{\alpha}$**, NB (4 h). vapour **IV**: **C$_{\alpha}$** to **I$_{\beta}$**, $PhNO_2$ (20 min), **C$_{\delta}$** to **I$_{\beta}$**, NB/THF/CH_3CN (1/1/1, *v*/*v*/*v*) (2 h). vapour **V**: **I$_{\beta}$** to **C$_{\alpha}$**, THF/CH_3CN (1/1, *v*/*v*) (24 h), **I$_{\beta}$** to **C$_{\delta}$**, Tol (8 h). vapour **VI**: **C$_{\alpha}$** to **M$_{\beta}$**, THF (3 min), **C$_{\alpha}$** to **M$_{\alpha}$**, NB (21 h), **C$_{\delta}$** to **M$_{\beta}$**, THF (10 min). vapour **VII**: **M$_{\beta}$** to **C$_{\alpha}$**, THF/CH_3CN (1/1, *v*/*v*) (30 min), **M$_{\alpha}$** to **C$_{\alpha}$**, THF/CH_3CN (1/1, *v*/*v*) (24 h), **M$_{\beta}$** to **C$_{\delta}$**, Tol (2 h), **M$_{\alpha}$** to **C$_{\delta}$**, Tol (12 h). vapour **VIII**: **I$_{\beta}$** to **M$_{\beta}$**, THF (5 min), **I$_{\beta}$** to **M$_{\alpha}$**, NB (21 h). vapour **IX**: **M$_{\beta}$** to **I$_{\beta}$**, NB/THF/CH_3CN (1/1/1, *v*/*v*/*v*) (4 h), **M$_{\alpha}$** to **I$_{\beta}$**, THF/CH_3CN (1/1, *v*/*v*) (1 min).

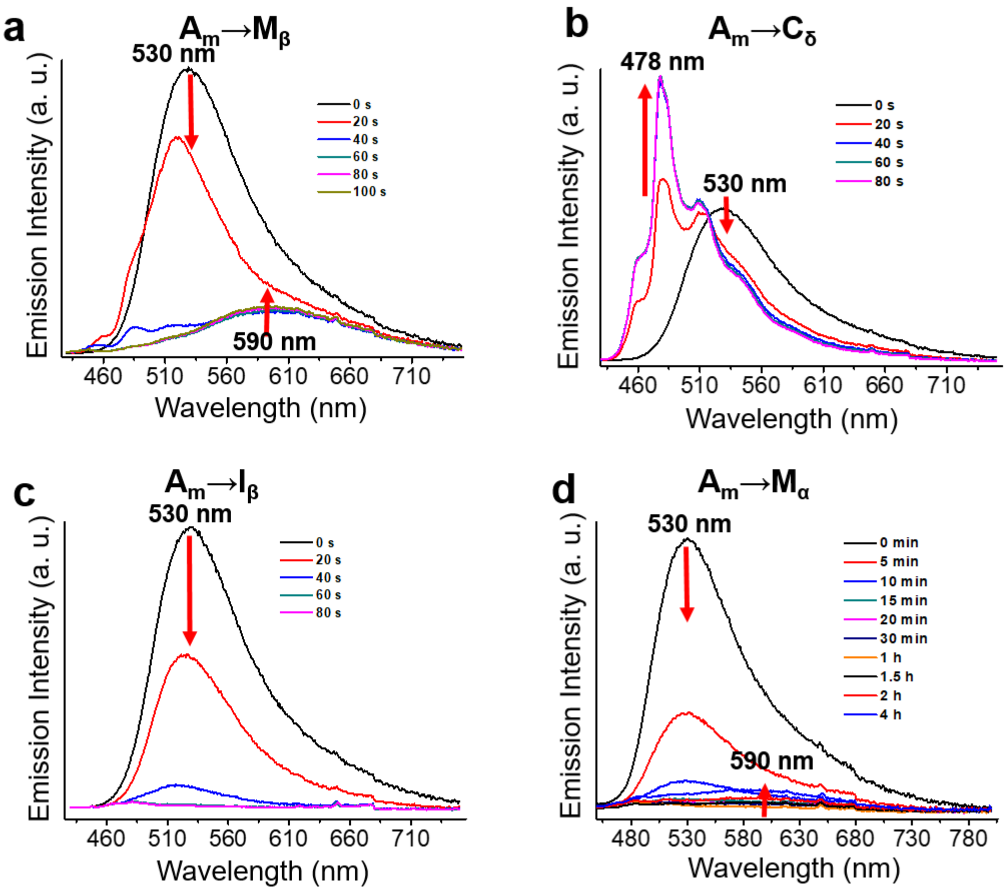

Supplementary Figure 42. Time-dependent emission spectra corresponding to the change from $\mathbf{A_m}$ to $\mathbf{M_\beta}$, $\mathbf{C_\delta}$, $\mathbf{I_\beta}$, or $\mathbf{M_\alpha}$ (λ_{ex} = 365 nm, voltage = 400 V, entrance slit width = 1 nm, exit slit width = 1 nm). **a**, $\mathbf{A_m}$ to $\mathbf{M_\beta}$, in THF vapour. **b**, $\mathbf{A_m}$ to $\mathbf{C_\delta}$, in toluene vapour. **c**, $\mathbf{A_m}$ to $\mathbf{I_\beta}$, in NB/THF/CH_3CN (1/1/1, *v*/*v*/*v*) vapour. **d**, $\mathbf{A_m}$ to $\mathbf{M_\alpha}$, in NB vapour.

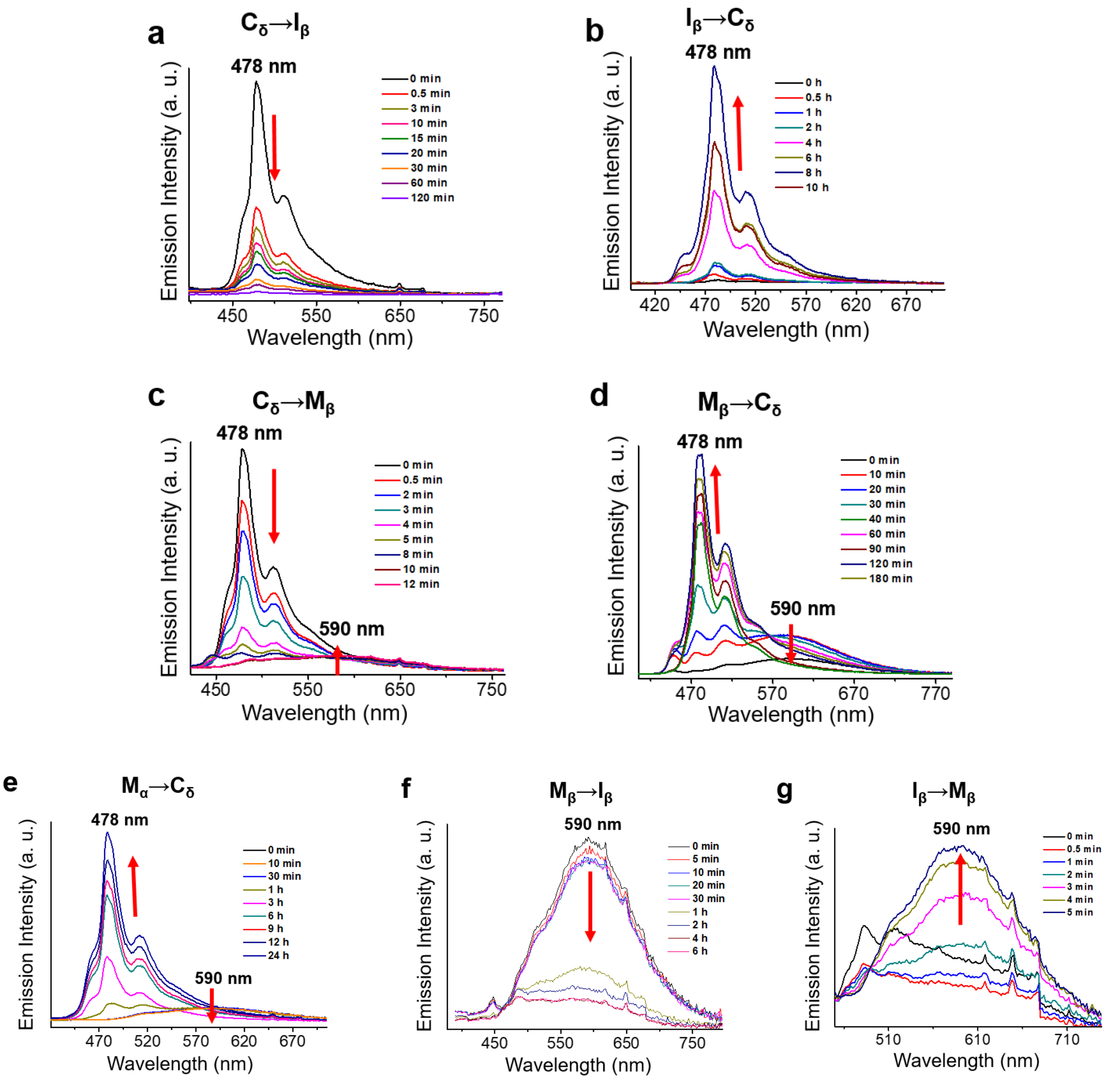

Supplementary Figure 43. Time-dependent emission spectra corresponding to the transformations between **C**$_{\delta}$, **I**$_{\beta}$, **M**$_{\beta}$, and **M**$_{\alpha}$ (λ_{ex} = 365 nm, voltage = 400 V, entrance slit width = 1 nm, exit slit width = 1 nm). **a**, **C**$_{\delta}$ to **I**$_{\beta}$, in NB/THF/CH_3CN (1/1/1, *v*/*v*/*v*) vapour. **b**, **I**$_{\beta}$ to **C**$_{\delta}$, in toluene vapour. **c**, **C**$_{\delta}$ to **M**$_{\beta}$, in THF vapour. **d**, **M**$_{\beta}$ to **C**$_{\delta}$, in toluene vapour. **e**, **M**$_{\alpha}$ to **C**$_{\delta}$, in toluene vapour. **f**, **M**$_{\beta}$ to **I**$_{\beta}$, in NB/THF/CH_3CN (1/1/1, *v*/*v*/*v*) vapour. **g**, **I**$_{\beta}$ to **M**$_{\beta}$, in THF vapour.

A summery of the photophysical properties and normalized emission spectra of **C**$_{\alpha}$, **C**$_{\beta}$, **C**$_{\gamma}$, **C**$_{\delta}$, **I**$_{\alpha}$, **A**$_{m}$, **I**$_{\beta}$, **M**$_{\alpha}$, **M**$_{\beta}$, and **D**$_{Py}$ is provided in the Supplementary Fig. 44, including the fluorescence

emission peak (λ_{em}), quantum yields (Φ_f), and fluorescent lifetime (τ_f). The experiments underlying the latter values are shown in Supplementary Fig. 45.

a

	Photos λ_{ex} = 365 nm	λ_{em} (nm)	Φ_f	τ_f (ns)
C_β or C_γ		0	0	0
C_α		482, 515, 560	0.39	2.0
C_δ		478, 513, 550	0.47	1.2
I_α		482, 515	0.09	2.4
A_m		530	0.44	2.0
I_β		482, 515	0.02	1.0
M_α		590	0.11	14
M_β		590	0.07	22
D_{Py}		590	0.12	28

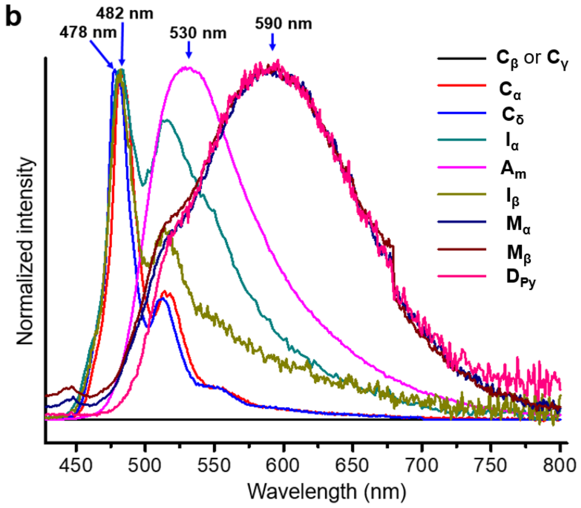

Supplementary Figure 44. Photophysical properties and normalized emission spectra of various solid forms considered in this study, namely **C_α**, **C_β**, **C_γ**, **C_δ**, **I_α**, **A_m**, **I_β**, **M_α**, **M_β**, and **D_{Py}**. **a,** Fluorescence images, emission peak (λ_{em}), quantum yields (Φ_f), and fluorescent lifetime (τ_f) determined using excitation at 365 nm. **b**, Normalized emission spectra (λ_{em} = 365 nm).

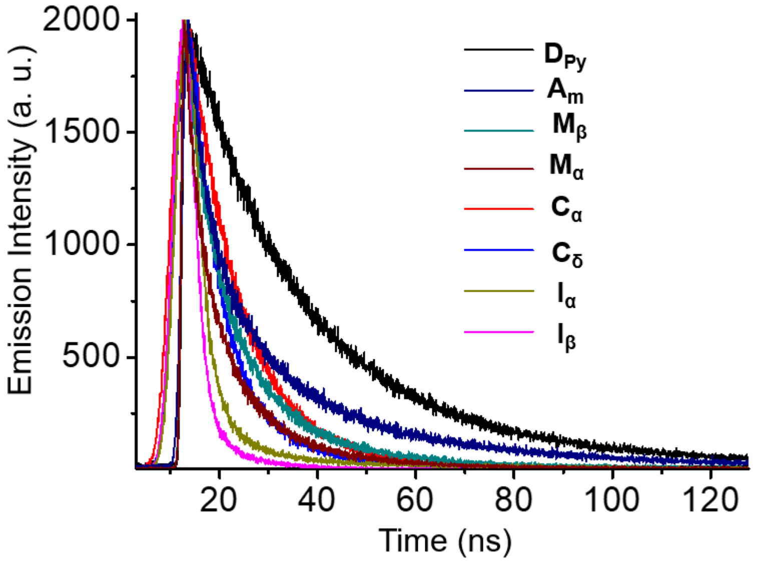

Supplementary Figure 45. Fluorescent lifetime (τ_f) determinations for **C_α**, **C_δ**, **I_α**, **A_m**, **I_β**, **M_α**, **M_β**, and **D_{Py}** (λ_{ex} = 365 nm, voltage = 400 V, entrance slit width = 1 nm, exit slit width = 1 nm).

A number of other organic solvent vapours (saturated in air at 298 K), including ethyl acetate (EA), acetone, *n*-hexane, *n*-octane, DMF, CH_3CH_2OH, cyclohexane, and DMSO, were found to

transform $\mathbf{A_m}$ into mixed crystalline $\mathbf{M_\beta}$ forms consisting of ***D*4d-CDMB-8⊐**solvents adducts co-crystallized with $\mathbf{D_{Py}}$. As above, the transformations that served to convert $\mathbf{A_m}$ into mixed crystalline species containing $\mathbf{D_{Py}}$ and ***D*4d-CDMB-8⊐**solvents were accompanied by a change in the emission maximum from λ_{em} = 530 nm to λ_{em} = 590 nm. Particularly noteworthy was that different time scales were required to effect equivalent levels of conversion (e.g., ethyl acetate (EA; 20 s), acetone and *n*-hexane (40 s), *n*-octane (60 s), DMF (80 s), CH_3CH_2OH (2 min), cyclohexane (3 min), DMSO (60 min)). Other co-crystalline species (i.e., ***D*4d-CDMB-8⊐Py•** solvent) produced by exposure of $\mathbf{A_m}$ to organic solvent vapours were found to produce a monomeric **Py** emission (around 480 nm) upon UV illumination. Again, a temporal effect was seen in response to specific organic solvent (e.g., chlorobenzene (1 min), DCM, anisole, dioxane, and bromobenzene (2 min), triethylamine (TEA; 10 min), and *m*-dibromobenzene (DB; 60 min)) under identical conditions of exposure (Supplementary Fig. 46).

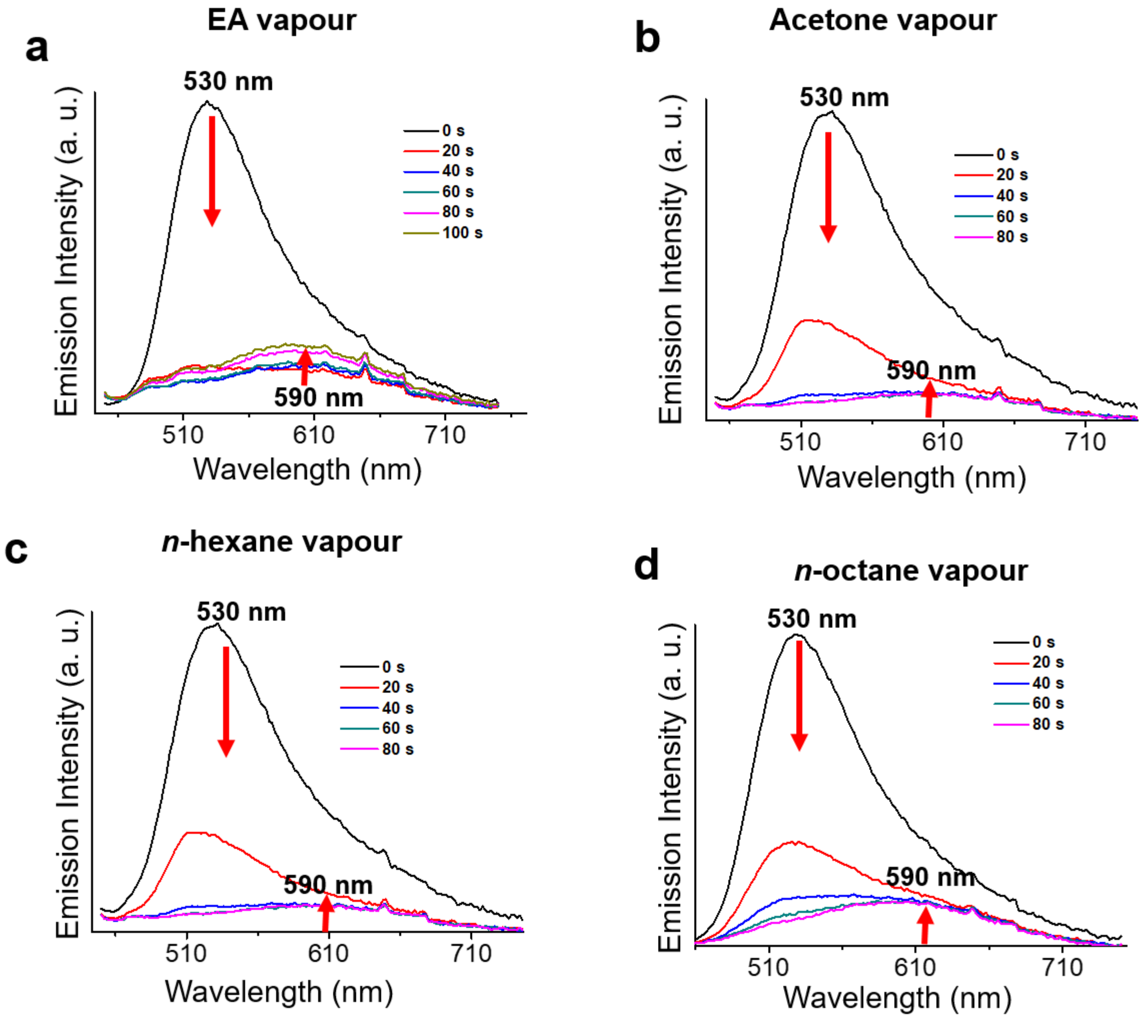

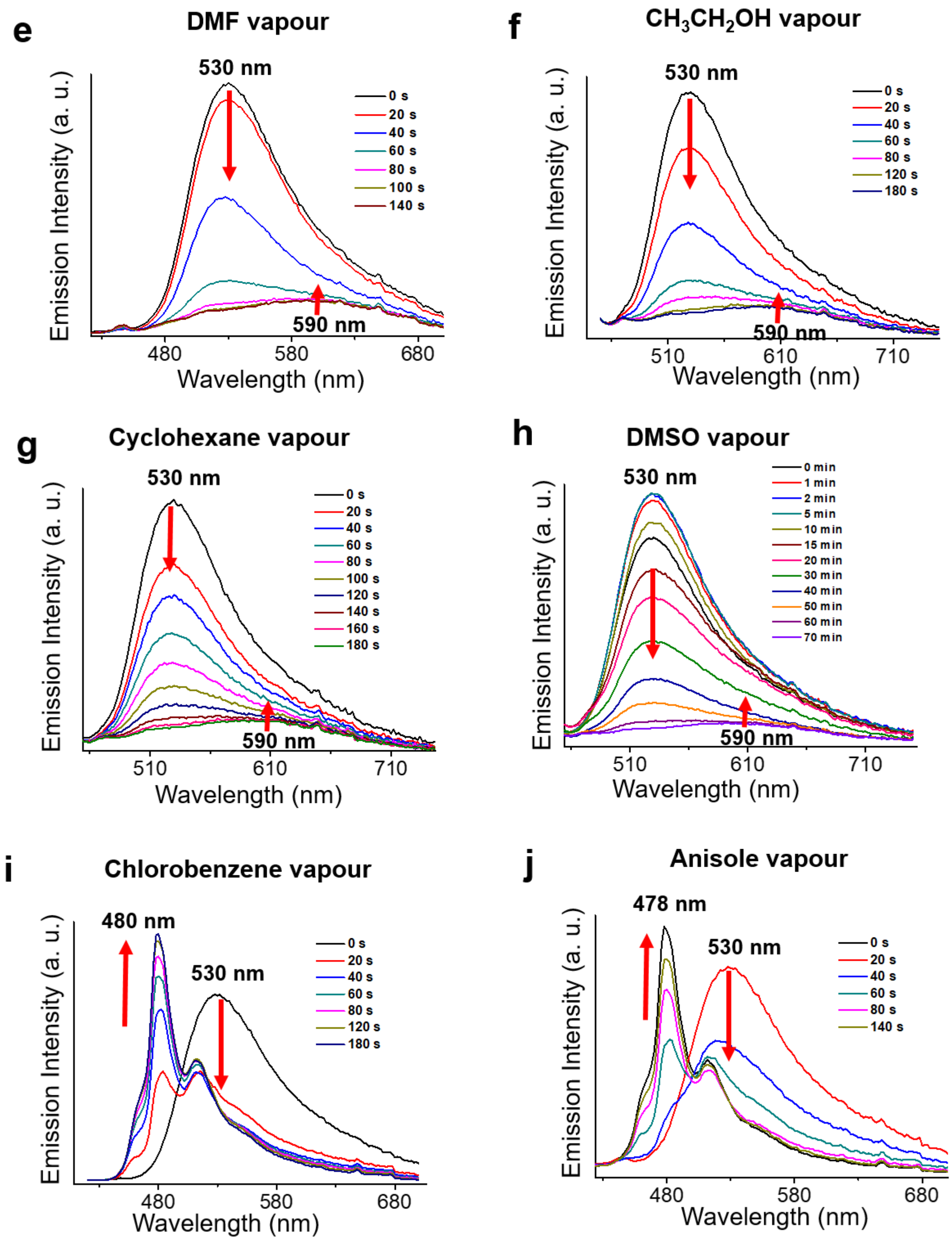
e
DMF vapour
530 nm
590 nm
0 s
20 s
40 s
60 s
80 s
100 s
140 s
Emission Intensity (a. u.)
Wavelength (nm)
480
580
680
f
CH_3CH_2OH vapour
530 nm
590 nm
0 s
20 s
40 s
60 s
80 s
120 s
180 s
510
610
710
g
Cyclohexane vapour
530 nm
590 nm
0 s
20 s
40 s
60 s
80 s
100 s
120 s
140 s
160 s
180 s
h
DMSO vapour
530 nm
590 nm
0 min
1 min
2 min
5 min
10 min
15 min
20 min
30 min
40 min
50 min
60 min
70 min
i
Chlorobenzene vapour
480 nm
530 nm
0 s
20 s
40 s
60 s
80 s
120 s
180 s
j
Anisole vapour
478 nm
530 nm
0 s
20 s
40 s
60 s
80 s
140 s

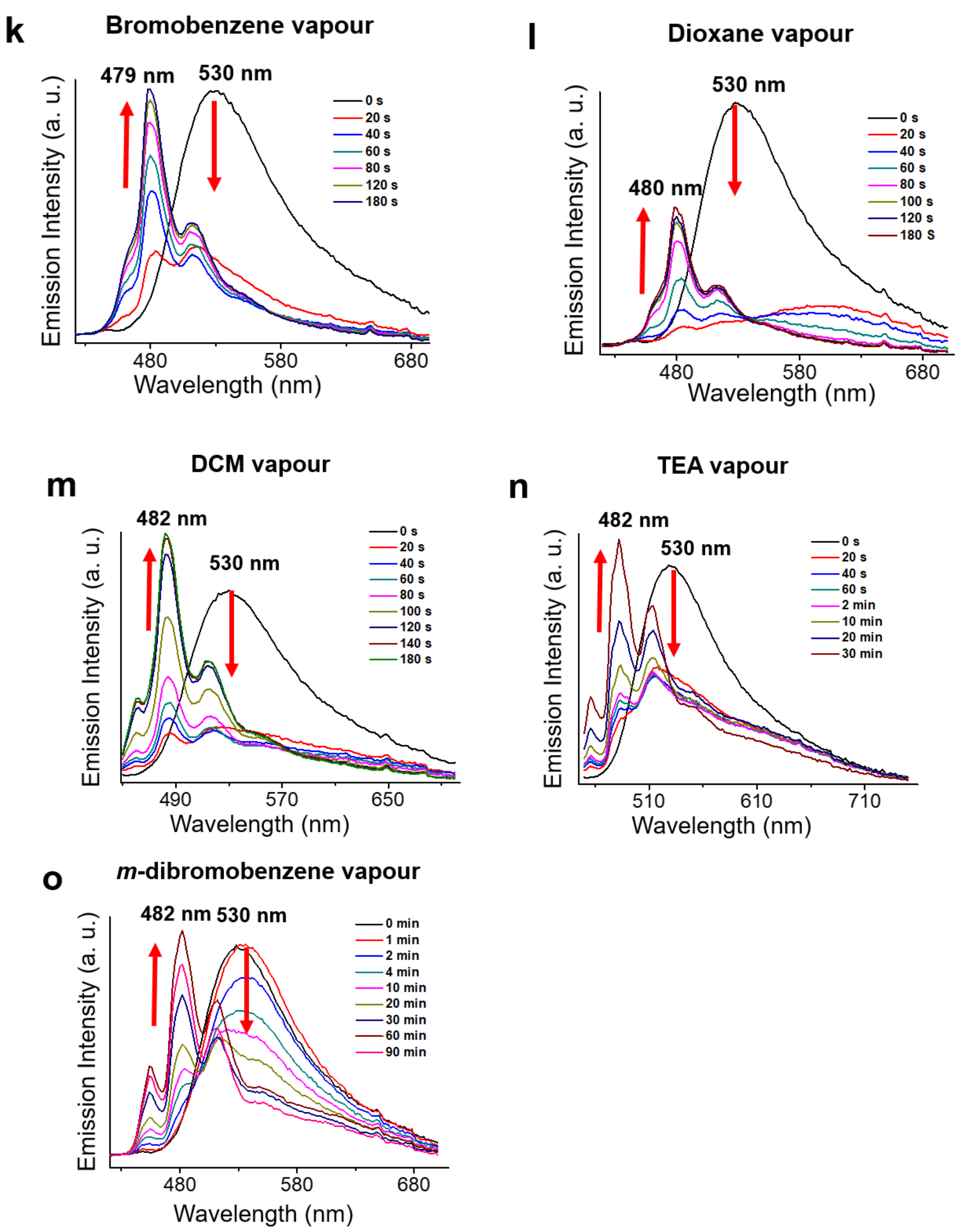

Supplementary Figure 46. Time-dependent emission spectra for **A**$_m$ recorded in the presence of various organic solvent vapours (e.g., ethyl acetate (**a**), acetone (**b**), *n*-hexane (**c**), *n*-octane (**d**), DMF (**e**), CH_3CH_2OH (**f**), cyclohexane (**g**), DMSO (**h**), chlorobenzene (**i**), anisole (**j**),

bromobenzene (**k**), dioxane (**l**), DCM (**m**), triethylamine (**n**), or *m*-dibromobenzene (**o**)). (λ_{ex} = 365 nm, voltage = 400 V, entrance slit width = 1 nm, exit slit width = 1 nm).

Section S5: Four dimensional information coding

In the main text, initial work showing the transformations from pattern **A** to **D** via THF/CH_3CN (1/1, *v*/*v*) vapour treatment, as well as the dynamic information storage in code **B** and **C** is presented (cf. Fig. 5). Further studies revealed the time dependent properties of the pattern transformations produced by different organic solvent vapours could be modified. For instance, by exposing code **A** to toluene or bromobenzene (PhBr) vapour, a series of dynamic patterns (namely $\mathbf{B_1}$-$\mathbf{D_1}$ or $\mathbf{B_2}$-$\mathbf{D_2}$) could be produced, albeit with different time scales (i.e., 1-30 min, 2-6 h, and greater than 12 h for toluene vs. 2-5 min, 15-60 min, and greater than 2 h for bromobenzene). It was also found that the same information (i.e., Info **I** and Info **II**) stored in codes **B** and **C** could also be hidden in other dynamic code pairs, such as i) $\mathbf{B_1}$ and $\mathbf{C_1}$ or ii) $\mathbf{B_2}$ and $\mathbf{C_2}$. This information (i.e., Info **I** and Info **II**) could then be produced and read out in a time-dependent manner in analogy to the experiments shown in main text Figure 5 through specific exposure to toluene or bromobenzene (Supplementary Fig. 47).

Alternatively, only a single information encoding response (i.e., only Info **I** or Info **II**, but not both) was produced in a time-dependent manner upon exposing code **A** to dioxane or *m*-dibromobenzene. This exposure allowed code **A** to be transformed into a designated information-storing code (i.e., code $\mathbf{B_3}$ or $\mathbf{C_3}$) as discussed in the main text (cf. Figure 5). Treatment with other organic solvent vapours (e.g., THF or NB/THF/CH_3CN (1/1/1, *v*/*v*/*v*)) also allowed code **A** to be transformed into other new patterns, namely **E**-**H**, and in a time-dependent manner (Supplementary Figs. 47 and 48).

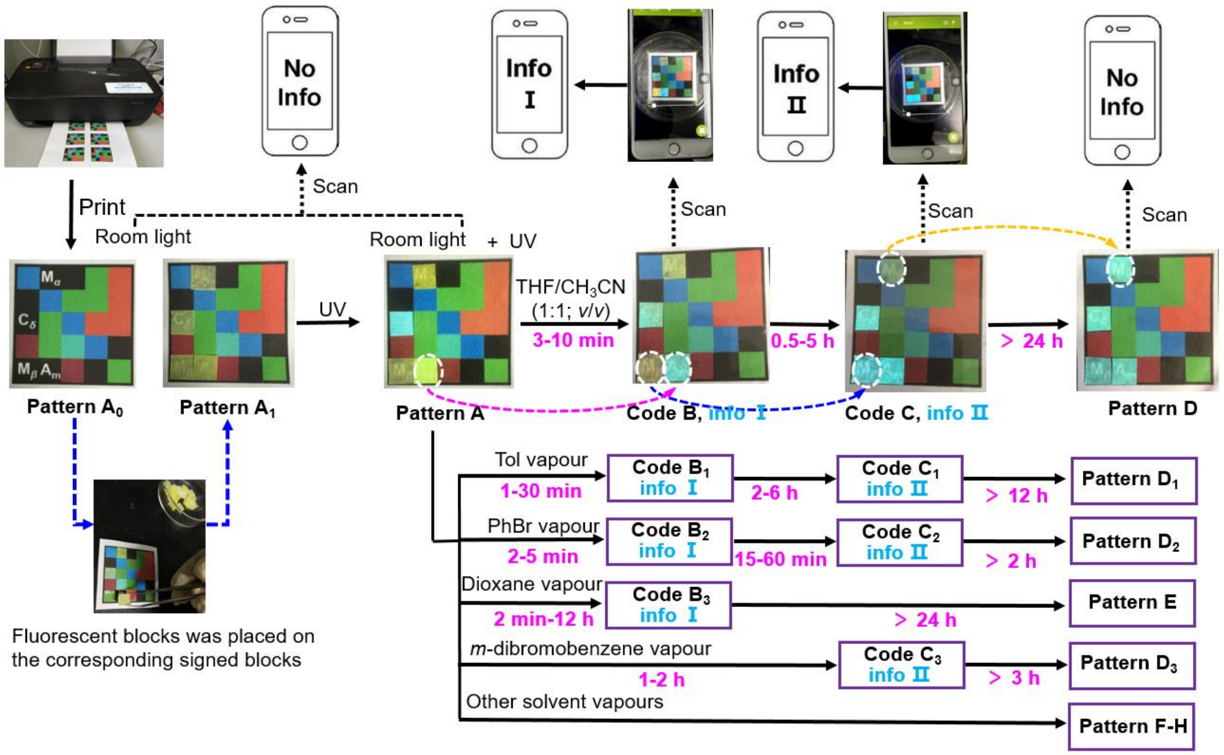

Supplementary Figure 47. Transformation and masking made possible by use of i) an original printed colour pattern, ii) natural and/or UV light, and iii) the time dependent organic vapour-induced changes in the luminescent features of the constituent code blocks, **A_m**, **M_α**, **M_β**, and **C_δ.**

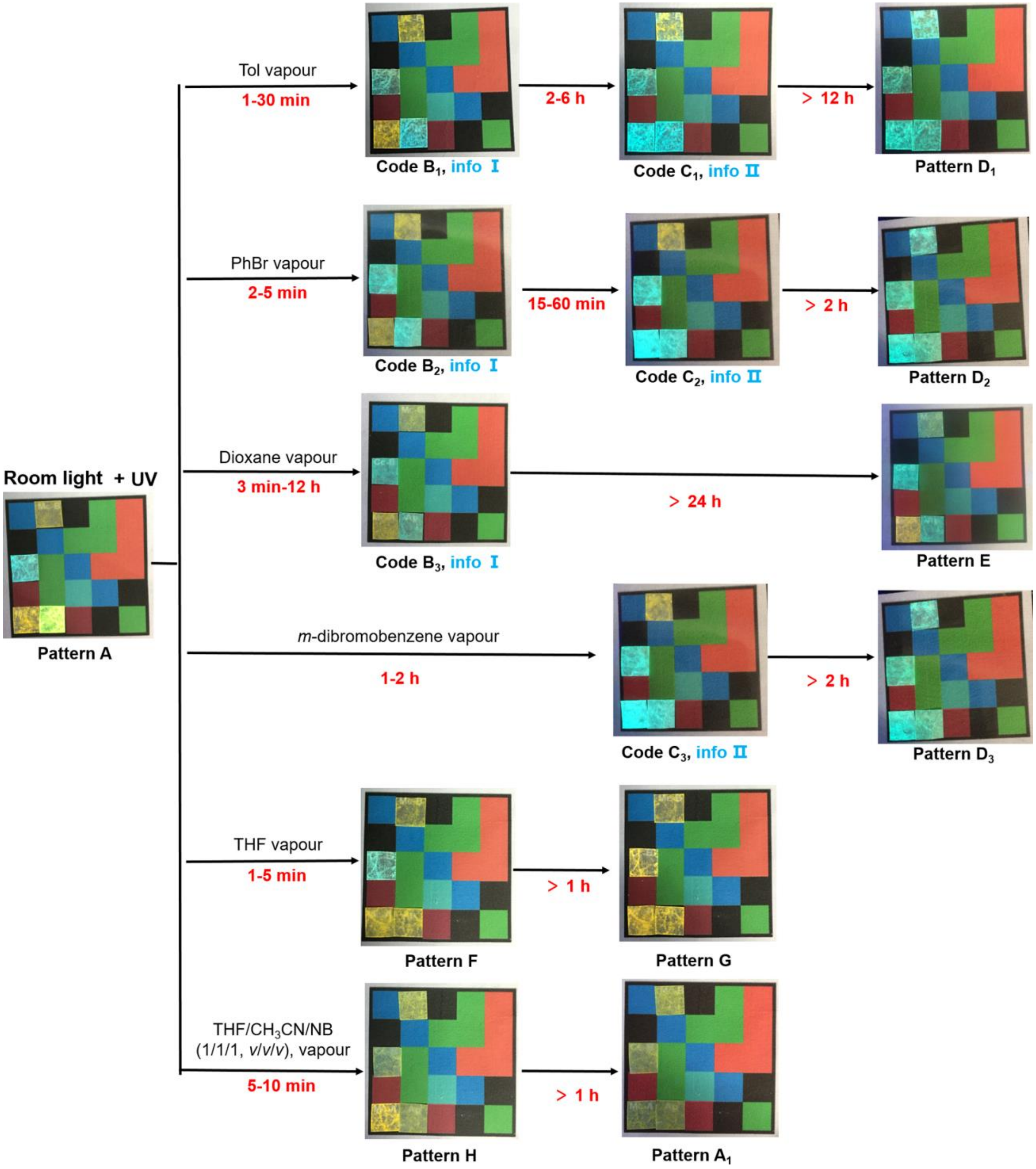

Supplementary Figure 48. Time-dependent transformations of pattern **A** in the presence of different organic solvent vapours under natural and UV light. The volume ratios of the mixed organic solvents were either 1:1 or 1:1:1.

Because of the differing stabilities of $\mathbf{C}_\alpha$ and the other materials considered in this study, time dependent dynamic 4D patterns with distinct timelines could be generated. For example, block $\mathbf{C}_\alpha$ could convert to block $\mathbf{I}_\beta$ in the air (298 K) as the result of solvent evaporation, a transformation that leads to an emission change form blue to green yellow in 1 hour. Thus, patterns containing $\mathbf{C}_\alpha$ block(s) (e.g., **B**-**D**) can be transformed into new patterns (e.g., **I** and **J**) by allowing the combined printed and block-containing array to sit in the air for 1 hour at 298 K (Supplementary Fig. 49). In contrast, other blocks, such as $\mathbf{A}_\mathbf{m}$, $\mathbf{C}_\delta$, $\mathbf{M}_\alpha$, and $\mathbf{M}_\beta$, display greater stability and retain their structure, emission features, and solvent vapour response characteristics even after allowing to stand on the bench for more than 24 h.

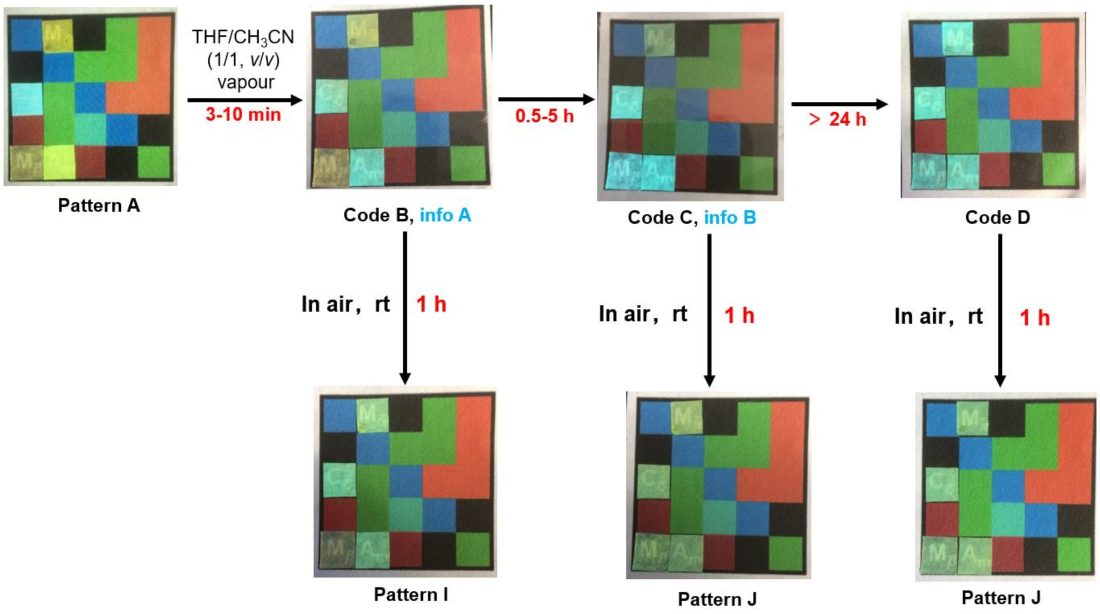

Supplementary Figure 49. Time-dependent transformation of codes **B**, **C**, and pattern **D** in air (298 K) as visualized under natural and UV light.

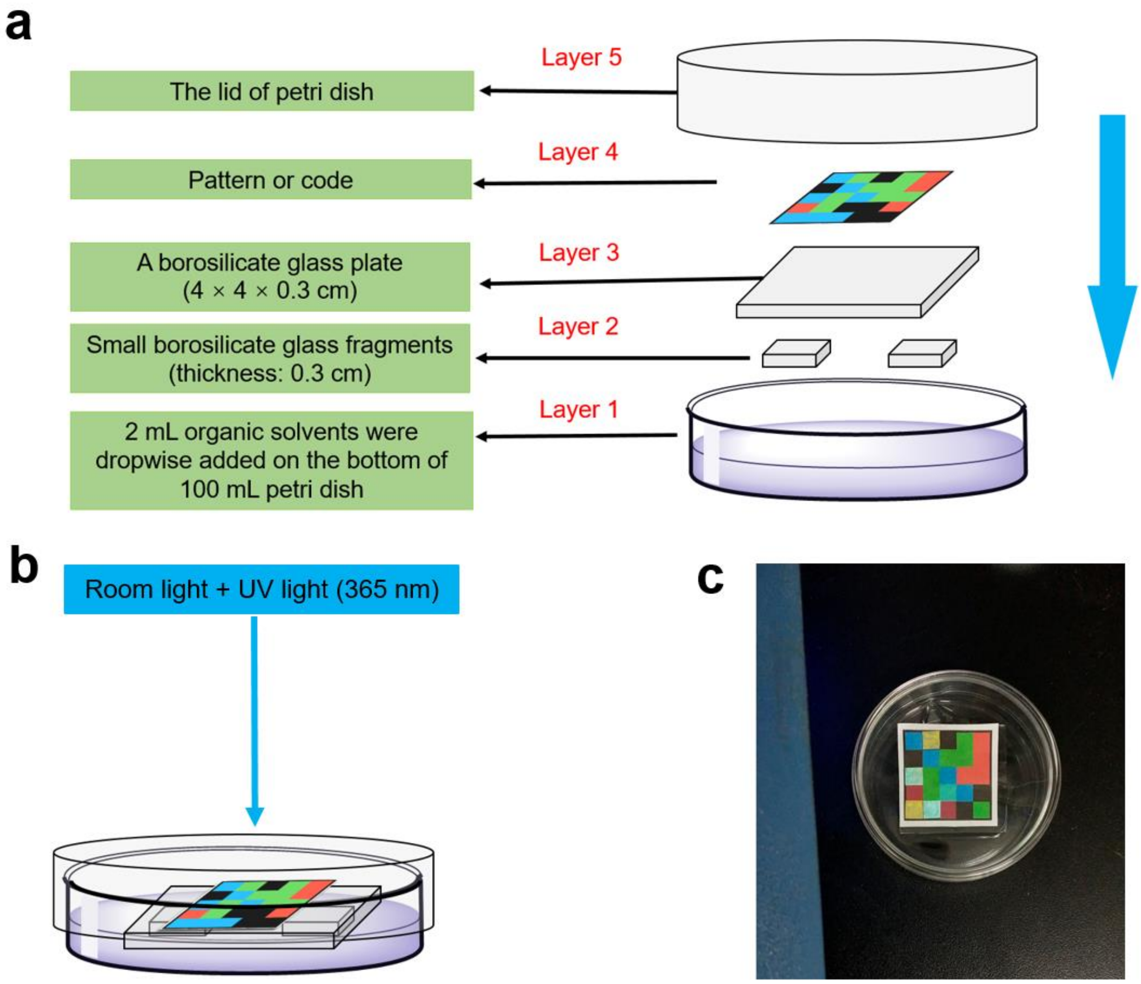

Supplementary Figure 50. **a**, **b**, Schematic representation and **c**, photograph of organic solvent vapours response studies showing the setup used.

Supplementary References

(1) Yang, Y.-D. & Gong, H.-Y. Thermally activated isomeric all-hydrocarbon molecular receptors for fullerene separation. *Chem. Commun.* **55**, 3701−3704 (2019).

(2) Hsieh, C.-T, Chen, C.-Y., Lin, H.-Y., Yang, C.-J. & Wang, C.-L. *et.al*. Polymorphic Behaviour of Perylene and Its Influences on OFET Performances. *J. Phys. Chem. C* **122**, 16242−16248 (2018).
