# Supplementary material for: Time-dependent solid-state molecular motion and colour tuning of host-guest systems by organic solvents
Source: Nat Commun. 2020 Jan 7;11:77. doi: 10.1038/s41467-019-13844-5 (PMC6946670; doi:10.1038/s41467-019-13844-5)
Supplement: Supplementary file 3 — Description of Additional Supplementary Files [file 41467_2019_13844_MOESM3_ESM.pdf]

Description of Additional Supplementary Files

File Name: Supplementary Movie 1
Description: Gram scale preparation of the co-crystalline material Cα.

File Name: Supplementary Movie 2
Description: Transformation of Cα into Mβ in the presence of THF vapour.

File Name: Supplementary Movie 3
Description: Transformation from pattern A0 to pattern A by adding fluorescent blocks or changing the light source.

File Name: Supplementary Movie 4
Description: Transformation from pattern A to pattern C with the treatment of THF/CH3CN (1/1, v/v) vapour
